# Evaluation of Dietary Patterns and All-Cause Mortality: A Systematic Review

**DOI:** 10.1001/jamanetworkopen.2021.22277

**Published:** 2021-08-31

**Authors:** Laural K. English, Jamy D. Ard, Regan L. Bailey, Marlana Bates, Lydia A. Bazzano, Carol J. Boushey, Clarissa Brown, Gisela Butera, Emily H. Callahan, Janet de Jesus, Richard D. Mattes, Elizabeth J. Mayer-Davis, Rachel Novotny, Julie E. Obbagy, Elizabeth B. Rahavi, Joan Sabate, Linda G. Snetselaar, Eve E. Stoody, Linda V. Van Horn, Sudha Venkatramanan, Steven B. Heymsfield

**Affiliations:** 1 Nutrition Evidence Systematic Review, Office of Nutrition Guidance and Analysis (ONGA), Center for Nutrition Policy and Promotion (CNPP), US Department of Agriculture (USDA) Food and Nutrition Service (FNS), Alexandria, Virginia; 2 Panum Group, Bethesda, Maryland; 3 Department of Epidemiology and Prevention, Wake Forest School of Medicine, Winston Salem, North Carolina; 4 Department of Nutrition Science, Purdue University, West Lafayette, Indiana; 5 Tulane University School of Public Health and Tropical Medicine, New Orleans, Louisiana; 6 Epidemiology Program, University of Hawai’i Cancer Center, Honolulu; 7 ONGA, CNPP, USDA FNS, Alexandria, Virginia; 8 Office of Disease Prevention and Health Promotion, Office of the Assistant Secretary for Health, US Department of Health and Human Services, Washington, DC; 9 Departments of Nutrition and Medicine, The University of North Carolina at Chapel Hill, Chapel Hill; 10 Nutritional Sciences, Human Nutrition, Food and Animal Sciences Department, College of Tropical Agriculture and Human Resources, University of Hawai’i at Mānoa, Honolulu; 11 Center for Nutrition, Healthy Lifestyles, and Disease Prevention, School of Public Health, Loma Linda University, Loma Linda, California; 12 Epidemiology, College of Public Health, University of Iowa, Iowa City; 13 Nutrition Division, Department of Preventive Medicine, Feinberg School of Medicine, Northwestern University, Chicago, Illinois; 14 Pennington Biomedical Research Center, Louisiana State University System, Baton Rouge

## Abstract

**Importance:**

The 2020 Dietary Guidelines Advisory Committee conducted a systematic review of existing research on diet and health to inform the current Dietary Guidelines for Americans. The committee answered this public health question: what is the association between dietary patterns consumed and all-cause mortality (ACM)?

**Objective:**

To ascertain the association between dietary patterns consumed and ACM.

**Evidence Review:**

Guided by an analytical framework and predefined inclusion and exclusion criteria developed by the committee, the US Department of Agriculture’s Nutrition Evidence Systematic Review (NESR) team searched PubMed, the Cochrane Central Register of Controlled Trials, and Embase and dual-screened the results to identify articles that were published between January 1, 2000, and October 4, 2019. These studies evaluated dietary patterns and ACM in participants aged 2 years and older. The NESR team extracted data from and assessed risk of bias in included studies. Committee members synthesized the evidence, developed conclusion statements, and graded the strength of the evidence supporting the conclusion statements.

**Findings:**

A total of 1 randomized clinical trial and 152 observational studies were included in the review. Studies enrolled adults and older adults (aged 17-84 years at baseline) from 28 countries with high or very high Human Development Index; 53 studies originated from the US. Most studies were well designed, used rigorous methods, and had low or moderate risks of bias. Precision, directness, and generalizability were demonstrated across the body of evidence. Results across studies were highly consistent. Evidence suggested that dietary patterns in adults and older adults that involved higher consumption of vegetables, fruits, legumes, nuts, whole grains, unsaturated vegetable oils, fish, and lean meat or poultry (when meat was included) were associated with a decreased risk of ACM. These healthy patterns were also relatively low in red and processed meat, high-fat dairy, and refined carbohydrates or sweets. Some of these dietary patterns also included intake of alcoholic beverages in moderation. Results based on additional analyses with confounding factors generally confirmed the robustness of main findings.

**Conclusions and Relevance:**

In this systematic review, consuming a nutrient-dense dietary pattern was associated with reduced risk of death from all causes.

## Key Points

**Question:**

What is the association between dietary patterns consumed and all-cause mortality?

**Findings:**

In this systematic review of 1 randomized clinical trial and 152 observational studies on dietary patterns and all-cause mortality, evidence demonstrated that dietary patterns characterized by increased consumption of vegetables, fruits, legumes, nuts, whole grains, unsaturated vegetable oils, fish, and lean meat or poultry (when meat was included) among adults and older adults were associated with decreased risk of all-cause mortality. These healthy patterns consisted of relatively low intake of red and processed meat, high-fat dairy, and refined carbohydrates or sweets.

**Meaning:**

This review found that a dietary pattern with nutrient-dense foods was associated with reduced risk of death from all causes.

## Teaser

This systematic review describes the combinations of foods, beverages, and food groups that were associated with beneficial outcomes, according to existing research on diet and nutrition.

## Introduction

Every 5 years, the US Department of Agriculture and Department of Health and Human Services convene the Dietary Guidelines Advisory Committee to review existing evidence on diet and health to inform the Dietary Guidelines for Americans. Over time, the committee’s focus has shifted away from single nutrients or foods and toward overall dietary patterns. Nutrient and food analyses cannot account for the interactions among or the degree of independent variation of coingested nutrients and food components.^1^ To advance nutrition research and inform the 2020-2025 Dietary Guidelines for Americans, understanding the role of dietary patterns in optimizing health and reducing the risk of chronic disease across the lifespan is a high priority.^2^

Dietary patterns are the quantities, proportions, variety, or combination of different foods, beverages, and nutrients in diets as well as the frequency with which they are habitually consumed.^3,4^ Different approaches can be used to study dietary patterns. A priori methods are based on scientific consensus or evidence-based approaches and use scores that reflect the degree of adherence.^5,6,7^ A posteriori methods identify which factors explain the variation in patterns or aggregate individuals into groups with nonoverlapping patterns.^8^ Other approaches can include hybrid methods, clinical trials that assign consumption to a specific pattern, and/or observational studies on food avoidance (eg, vegetarian diets).

The 2015 committee reviewed the associations between dietary patterns and multiple outcomes, including cause-specific mortality from cardiovascular disease (CVD) and dementia.^3^ Other literature reviews have examined specific diets or foods and cause-specific mortality.^9,10,11,12^ In line with these studies, the 2020 committee,^13^ with support from the Nutrition Evidence Systematic Review (NESR) team of the US Department of Agriculture, conducted a systematic review of the literature to ascertain the association between dietary patterns consumed and all-cause mortality (ACM).

## Methods

The systematic review question was, “what is the relationship between dietary patterns consumed and ACM?” It also addressed the topic of diets on the basis of macronutrient distribution. Presented in full detail elsewhere,^14^ the review used NESR systematic review methods, which are summarized herein. We followed the Preferred Reporting Items for Systematic Reviews and Meta-analyses (PRISMA) reporting guideline.

**Figure 1.**
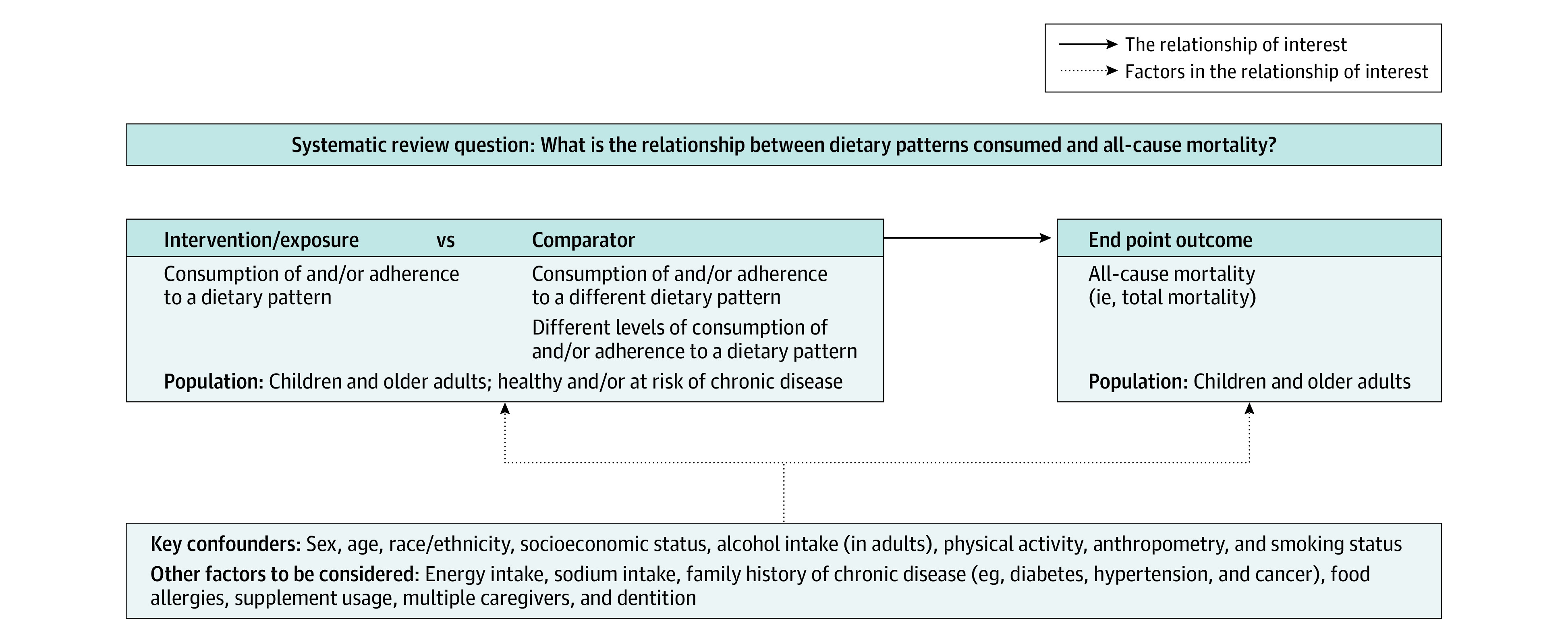
Analytic Framework for the Systematic Review Question This analytic framework visually represents the overall scope of the systematic review question and depicts the contributing elements that were examined and evaluated, including the target population, exposure, comparison, outcomes, and key confounders. Reproduced from the 2020 Dietary Guidelines Advisory Committee and Nutrition Evidence Systematic Review Team.^14^

The committee developed a protocol, including an analytical framework (Figure 1) and inclusion and exclusion criteria (Table). The protocol was made available for public comment and was discussed by the committee in public meetings before evidence synthesis.

**Table.** Inclusion and Exclusion Criteria for Relevant Articles That Examined Dietary Patterns and All-Cause Mortality^a^

Category	Inclusion criteria	Exclusion criteria
Study design	Randomized clinical trials Nonrandomized controlled trials, including quasi-experimental and controlled before-and-after studies Prospective cohort studies Retrospective cohort studies Nested case-control studies	Uncontrolled trials Case-control studies Cross-sectional studies Uncontrolled before-and-after studies Narrative reviews Systematic reviews Meta-analyses
Intervention/exposure	Studies that examined consumption of and/or adherence to a Dietary pattern (ie, the quantities, proportions, variety, or combination of different foods, drinks, and nutrients, when available in diets, and the frequency with which they are habitually consumed), including, at a minimum, a description of the foods and beverages in the pattern Dietary patterns may be measured or derived using a variety of approaches, such as adherence to a priori patterns (indices/scores), data-driven patterns (factor or cluster analysis), reduced rank regression, or other methods, including clinical trials and/or Diet based on macronutrient distribution outside of the AMDR^b^^,^^c^ including the macronutrient distribution of carbohydrate, fat, and protein of the diet, and at least 1 macronutrient outside of the AMDR	Studies that Did not provide a description of the dietary pattern, which at minimum, must include the foods and beverages in the pattern (ie, studies that examined labeled dietary patterns, but did not describe the foods and beverages consumed) Examined consumption of and/or adherence to a diet based on macronutrient proportion in which all macronutrients were within the AMDR Did not describe the entire macronutrient distribution of the diet (ie, studies that only examined a single macronutrient vs outcomes)
Comparator	Dietary patterns described by foods and beverages consumed Consumption of and/or adherence to a different dietary pattern Different levels of consumption of and/or adherence to a dietary pattern Diets described by macronutrient distribution Different macronutrient distribution of carbohydrate, fat, and protein	NA
Outcomes	Studies that reported ACM (ie, total mortality): the total number of deaths from all causes during a specific period	Studies that only reported cause-specific mortality (ie, total number of deaths from a specific disease, such as cardiovascular disease or cancer)
Date of publication	January 2000 to October 2019	Articles published before January 2000 or after October 2019
Publication status	Work that had been peer reviewed and published in peer-reviewed journals	Work that had not been peer reviewed and had not been published in peer-reviewed journals, including unpublished data, manuscripts, preprints, reports, abstracts, and conference proceedings
Language of publication	Published in English	Published in languages other than English
Country of origin^d^	Conducted in countries ranked as high or higher human development	Conducted in countries ranked as medium or lower human development
Study participants	Human participants Male participants Female participants	Nonhuman participants (ie, animals) Women during pregnancy and lactation
Age of study participants	Age at intervention or exposure: Children and adolescents (aged 2-18 y) Adults (aged 19-64 y) Older adults (aged 65 y or older) Age at outcome: Children and adolescents (aged 2-18 y) Adults (aged 19-64 y) Older adults (aged 65 y or older)	Age at intervention or exposure: Infants and toddlers (birth to 24 mo) Age at outcome: Infants and toddlers (birth to 24 mo)
Health status of study participants	Studies that enrolled participants who were healthy and/or at risk for chronic disease, including those with obesity Studies that enrolled some participants who were diagnosed with a disease	Studies that exclusively enrolled participants who were diagnosed with a disease or hospitalized with illness or injury. (For this criterion, studies that exclusively enrolled participants with obesity were included.)

Abbreviations: ACM, all-cause mortality; AMDR, acceptable macronutrient distribution range; NA, not applicable.

^a^ Adapted from the 2020 Dietary Guidelines Advisory Committee and Nutrition Evidence Systematic Review Team.^14^

^b^ Data from Trumbo et al.^15^

^c^ Macronutrient percentage of energy outside of the AMDR were as follows: (1) carbohydrate for all age groups: <45% or >65% of energy; (2) protein for children aged 1-3 y: <5% or >20% of energy, protein for children aged 4-18 y: <10% or >30% of energy, protein for adults aged ≥19 y: <10% or >35% of energy; and (3) fat for children aged 1-3 y: <30% or >40% of energy, fat for children aged 4-18 y: <25% or >35% of energy, fat for adults aged ≥19 y: <20% or >35% of energy.

^d^ The classification for countries was based on the Human Development Index (HDI) from the year the study intervention occurred or data were collected.^16^ If the study did not report the year in which the intervention occurred or data were collected, the HDI classification for the year of publication was applied. HDI values were available from 1980 and then from 1990 to present. If a study was conducted before 1990, the HDI classification from 1990 was applied. If a study was conducted in 2018 or 2019, the most current HDI classification was applied. When a country was not included in the HDI ranking, the current country classification from the World Bank^17^ was used instead.

The NESR librarians developed and implemented literature searches in PubMed, the Cochrane Central Register of Controlled Trials, and Embase to identify articles that were published from January 1, 2000, to October 4, 2019.^14^ These studies evaluated dietary patterns and ACM in participants aged 2 years and older. Two NESR analysts independently screened the search results to identify articles that met the predetermined criteria (Table), and then they manually searched the reference lists of the included articles. The NESR staff extracted data from and completed 2 independent risk-of-bias assessments of each included article.

The 2020 committee synthesized the evidence qualitatively. The evidence was synthesized not by diet type or label but rather by similarities and differences in the foods and beverages that made up the dietary patterns that were examined. This synthesis approach was taken because the results of this systematic review were intended to inform dietary guidance and to allow for conclusions to be drawn regarding which dietary pattern components were most associated with risk of ACM.

The results of the systematic review were organized according to a dietary pattern approach (a priori, a posteriori, and other). Based on the synthesized evidence, the committee developed conclusion statements to answer the systematic review question and graded the strength of evidence for the conclusions as follows: strong, moderate, limited, or grade not assignable. Grades were assigned using the NESR’s predefined criteria for grading elements (risks of bias, consistency, directness, precision, and generalizability) and approach, which takes study design into consideration. Future research recommendations for strengthening the body of evidence were based on the gaps and limitations that were identified during the systematic review process.

## Results

### Study Characteristics

**Figure 2.**
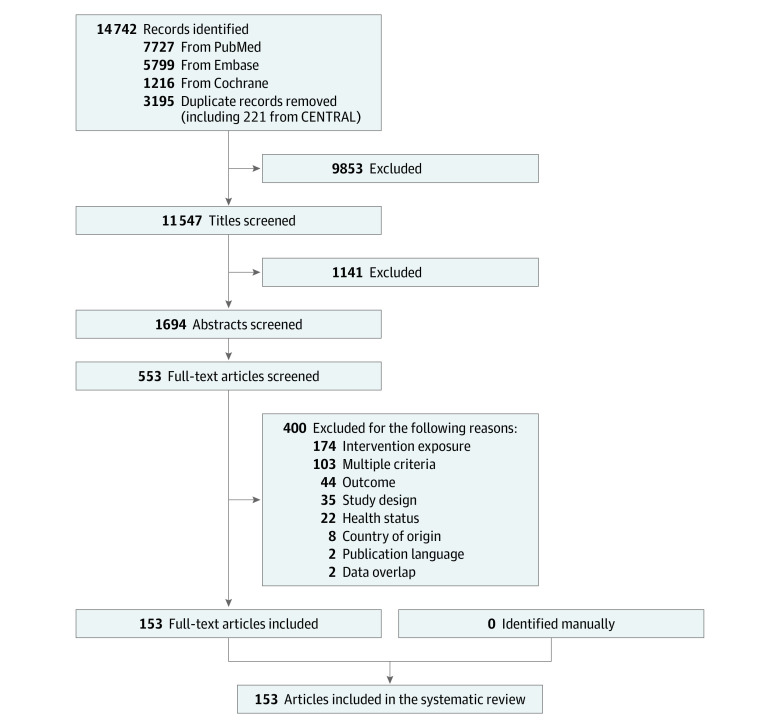
Literature Search and Screening Flowchart The literature search yielded 11 547 studies after the removal of duplicate articles. Dual screening resulted in the exclusion of 400 full-text articles or at least 1 reason, which may not reflect all possible reasons. The body of evidence included 153 articles.

After dual screening, 11 547 relevant studies were identified. The body of evidence included 153 articles (involving 6 550 664 individuals), of which 1 was from a randomized clinical trial (RCT)^18^ and 152 were from observational studies^19,20,21,22,23,24,25,26,27,28,29,30,31,32,33,34,35,36,37,38,39,40,41,42,43,44,45,46,47,48,49,50,51,52,53,54,55,56,57,58,59,60,61,62,63,64,65,66,67,68,69,70,71,72,73,74,75,76,77,78,79,80,81,82,83,84,85,86,87,88,89,90,91,92,93,94,95,96,97,98,99,100,101,102,103,104,105,106,107,108,109,110,111,112,113,114,115,116,117,118,119,120,121,122,123,124,125,126,127,128,129,130,131,132,133,134,135,136,137,138,139,140,141,142,143,144,145,146,147,148,149,150,151,152,153,154,155,156,157,158,159,160,161,162,163,164,165,166,167,168,169,170^(Figure 2). Multiple articles used data from the same study but used different methods or represented unique subsamples or dietary patterns. Studies enrolled adults and older adults (aged 17-84 years at baseline) from 28 countries with a high or very high Human Development Index^16^ classification (Australia, Belgium, Canada, Czech Republic, Croatia, Denmark, Finland, France, Germany, Greece, Hong Kong, Hungary, Iran, Italy, Japan, Korea, Norway, Netherlands, Poland, Portugal, Russia, Serbia, Singapore, Spain, Sweden, Switzerland, United Kingdom, and United States). Fifty-three studies^21,23,24,27,29,32,33,37,39,40,42,43,44,46,47,52,56,57,58,59,60,61,72,73,74,78,83,86,94,95,96,98,100,101,102,104,107,110,118,125,126,127,129,135,137,150,158,160,161,163,166^ originated from the US. Some studies exclusively enrolled female ^29,37,46,58,67,74,82,86,99,124,127,128,135,144,163^ or male^22,31,40,54,62,80,81,105,106,110,118,130,141,142,147,153^ participants.

The approaches for assessing dietary patterns included an RCT^18^; a priori methods, such as index or score analysis^19,20,21,22,23,24,25,26,27,28,29,30,31,32,33,34,35,36,37,38,39,40,41,42,43,44,45,46,47,48,49,50,51,52,53,54,55,56,57,58,59,60,61,62,63,64,65,66,67,68,69,70,71,72,73,74,75,76,77,78,79,80,81,82,83,84,85,86,87,88,89,90,91,92,93,94,95,96,97,98,99,100,101,102,103,104,105,106,107,108,109,110,111,112,113,114,115,116,117,118,119,120,121,122,123,124,125,126,127,128^; a posteriori methods, such as factor or cluster analysis^29,36,56,93,109,129,130,131,132,133,134,135,136,137,138,139,140,141,142,143,144,145,146,147,148^; and other methods,^136,149,150,151,152,153,154,155,156,157,158^ such as reduced rank regression and comparisons by animal product avoidance or ultraprocessed food consumption. eTable 1 in the Supplement describes the 185 dietary patterns that were examined. The ACM data were collected through validated methods (population-level registries, electronic databases, or medical or historical records) or active follow-up.

### Evidence Synthesis

#### A Priori Studies

In the RCT, participants at high-risk for CVD were randomized to a Mediterranean diet with extra-virgin olive oil or mixed nuts or to a control diet.^18^ Consumption of either intervention diet was associated with reduced ACM risk after a median follow-up of 4.8 years. Results were similar in subanalyses, which removed participants with protocol deviations and randomization issues. Most included studies of dietary patterns (110 of 153 [72%])^19,20,21,22,23,24,25,26,27,28,29,30,31,32,33,34,35,36,37,38,39^ used index or score analysis. Many different measures were reported, including variations of Mediterranean diet (n = 31), DASH (Dietary Approaches to Stop Hypertension) diet scores (n = 1), country-specific indices (n = 16), Healthy Eating Index or Dietary Guidelines for Americans scores (n = 7), and other indices or scales (n = 24) (eTable 1 in the Supplement). A complete description of these components and the scoring procedures has been published elsewhere.^14^

Findings were consistent across the studies, despite the variety of indices or scores used (eTable 2 in the Supplement). Nutrient-dense dietary patterns, regardless of pattern label or name, were associated with significantly lower ACM risk. For example, all 12 comparisons that examined DASH diet scores suggested that higher adherence was associated with lower ACM risk,^26,46,47,48,52,69,84,88,98,101,107,127^ and 54 of the 63 comparisons that were reported in 44 articles that examined a Mediterranean-type index or score suggested that higher adherence was associated with lower ACM risk.^24,25,30,32,34,35,46,47,48,49,50,51,52,53,63,64,68,69,71,76,79,81,83,88,97,98,104,106,107,108,111,112,113,114,115,116,117,122,123,124,125,127,128,141^

A few articles reported nonsignificant results with similar patterns.^21,31,33,36,39,42,62,93,102,110^ Common elements of these dietary indices or scores were (1) relatively higher intake of vegetables (with or without potatoes); legumes; fruit; nuts; either whole grains specifically, cereals unspecified, or nonrefined grains; fish and/or seafood; lean meat or poultry, when included (Dietary Guidelines for Americans or Healthy Eating Index scores reflect total protein foods and/or ratio of white to red meat); and unsaturated fats vs saturated fats; and (2) relatively lower intake of red and processed meat and/or meat and meat products; refined grains; added sugars and/or sugar-sweetened beverages; solid fats, saturated fats, and/or trans fat; and excessive sodium.

Many of the indices considered alcoholic beverage intake in low to moderate amounts or within a threshold (eg, 10-25 g/d; 0.5-1.5 drinks/d for women and 0.5-2.0 drinks/d for men) as a positive component within the context of the entire dietary pattern. However, measurement and scoring procedures varied between indices.

The associations reported between a priori–derived dietary patterns and ACM remained in sensitivity or subgroup analyses after combining the dietary patterns with anthropometry, physical activity, and/or smoking status^24,35,37,44,53,118^; performing stratification or additional adjustment for anthropometry, sex, age, educational level, race/ethnicity, and/or smoking status^25,34,37,46,47,52,76,83,85,86,87,95,96,98,99,104,111,113,115,119,125,126^; excluding early deaths or the first few years of follow-up results^25,35,49,57,58,63,65,72,76,82,96,99,111,112,113,119,125,128^; accounting for chronic disease status (eg, diabetes or CVD^28,30,34,37,51,58,64,65,71,72,80,96,111,125^); or underreporting or misreporting chronic disease.^28,34,106,112^ When adherence to dietary patterns was combined with other healthier lifestyle factors (eg, not smoking and meeting recommended physical activity levels), stronger associations were typically observed. For example, participants who adhered to a Mediterranean diet met recommended physical activity recommendations, were long-term nonsmokers, and had a lower mortality risk (relative risk [RR], 0.65; 95% CI, 0.63-0.68) compared to those that only followed the Mediterranean diet (RR, 0.86; 95% CI, 0.83-0.88).^24^

#### A Posteriori Studies

Most of the 25 articles (19 [76%]) that examined dietary patterns by using factor or cluster analysis reported significantly lower ACM risk when comparing higher with lower adherence to the same derived pattern^29,56,93,130,131,134,135,139,140,141,142,143,144,145,146,147,148^ or to different patterns^109,138^ (eTable 2 in the Supplement). For example, participants with higher vs lower adherence to the Prudent pattern had lower mortality risk (Quintile 1: reference; Q2: RR, 0.85 [95% CI, 0.78-0.92; Q3: RR, 0.84 [95% CI, 0.78-0.91]; Q4: RR, 0.81 [95% CI, 0.74-0.88]; Q5: RR, 0.83 [95% CI, 0.76-0.90]; *P* for trend <.001).^135^ Participants with higher vs lower adherence to the Western pattern had higher mortality risk (Q1: reference; Q2: RR, 1.00 [95% CI, 0.92-1.08]; Q3: RR, 1.10 [95% CI, 1.02-1.20]; Q4: RR, 1.16 [95% CI, 1.06-1.26]; Q5: RR, 1.21 [95% CI, 1.12-1.32]; *P* for trend <.001).^135^ Dietary patterns were given different labels, such as healthy, prudent, and Mediterraneanlike, but were similarly characterized by higher intake of vegetables, fruits, fish or other seafood, legumes and/or whole grains, nuts, vegetable or olive oils, and/or poultry (eg, white meat, such as chicken or turkey^140^).

Several articles (5 of 25 [20%]) reported that the dietary patterns associated with significantly higher ACM risk (and/or shorter survival^129,130,133,135,148^) emphasized the following commonalities: higher intake of (1) meat and meat products such as beef, pork, sausage^133^; red meat and meat products^130^; red meat and processed meats^135^; fresh and processed meats and seafood^148^; (2) high-fat dairy products such as ice cream, cheese, and whole milk^129^; and/or (3) refined grains^130,135^ or flour-based foods such as pastries^133^ and/or sweets and desserts^133,135,148^ such as cake, cookies, chocolate, and candy^129^; as well as lower intake of (4) low-fat dairy products, rice and pasta, fruits, fish and other seafood, and dark green vegetables.^129^

Results were similar whether stratified by country,^131^ after excluding participants with CVD or cancer at baseline,^147^ or in models that accounted for co-twin pairs.^133^ Stratification by sex was inconsistent, with no significant associations in women only but significant associations in pooled analyses.^138^ For example, Krieger et al^138^ reported lower risk of ACM at mean follow-up of 25 years in overall analyses, with men and women pooled and when comparing fish (hazard ratio [HR], 0.87; 95% CI, 0.78-0.97) and traditional (HR, 0.89; 95% CI, 0.80-0.98) dietary patterns to the sausage and vegetables dietary pattern (reference; HR, 1.0). However, in the Krieger et al^138^ analyses, which were stratified by sex, the results in women only were not statistically significant: sausage and vegetables pattern (reference; HR, 1.0) was compared with meat and salad (HR, 0.93; 95% CI, 0.80-1.08), fish (HR, 0.98; 95% CI, 0.83-1.15), traditional (HR, 1.02; 95% CI, 0.87-1.19), and high-fiber foods (HR, 0.91; 95% CI, 0.79-1.05) patterns.

Nonsignificant associations were reported between a posteriori–derived dietary patterns and ACM, although the direction generally aligned with the significant results we have described, and may be attributed to smaller sample sizes,^137^ the sample evaluated,^138^ or the gradient between exposure comparisons.^135^

#### Other Studies

Dietary patterns that were derived from reduced rank regression showed inconsistent results (eTable 2 in the Supplement).^136,150,153^ Five articles examined the dietary patterns based on avoidance of animal-based products (eg, vegetarian diets).^149,151,154,155,158^ Vegetarian or plant-based patterns were associated with significantly lower ACM risk in 2 studies,^155,158^ but no significant associations were found in other exposure groups (eg, vegetarian vs pescovegetarian, and meat eaters vs vegetarian or vegan).^149,151,154^

Three studies reported that the dietary pattern of higher vs lower consumption of ultraprocessed foods was associated with higher ACM risk^152,156,157^ as defined by the fourth level of the NOVA Food Classification System. These patterns differed by the foods comprising the pattern (eg, some of which included highly palatable foods, such as ice cream and processed meats, and others included artificial flavors and texturizing agents), reducing the generalizability of these patterns and thus the results.

### Assessment of Evidence

Most studies were conducted with rigorous methods and at low or moderate risks of bias across domains.^14^ Most studies accounted for potential confounders except for race/ethnicity, which was often homogenous or not reported. In addition, most studies assessed dietary intake once and were, therefore, at risk of bias because of potential changes in dietary patterns among participants over time. However, these studies used validated methods, and their results aligned with findings of studies that assessed diet over time. Several studies did not account for missing data, primarily diet or exposure data at baseline. Participants with implausible energy intake, incomplete dietary data, and/or a history or presence of chronic diseases or medical conditions at baseline were typically removed from the studies or analyses.

Dietary patterns that were examined with different approaches showed consistent direction and magnitude of associations with ACM. Precision was indicated by relatively narrow CIs between studies. Most studies did not report power analyses or sample size calculations but reported analytic sample sizes that were large enough to investigate the association, ranging from 161 participants^68^ to 451 256 participants.^69^ Total number of deaths ranged from 53 with approximately 4 years of total follow-up^38^ to 51 702 with approximately 13 to 18 years of follow-up.^69^ Although the incident number of deaths differed between studies, the number of events reported within groups confirmed precision across the body of evidence. The evidence base had directness and was generalizable to the US population. Results may be less generalizable to younger or less healthy populations. Results are likely generalizable to adults of various underreported racial/ethnic backgrounds, although it is difficult to ascertain exactly how race and/or ethnicity was involved in the association between dietary patterns and ACM because many studies did not report that information.

The 2020 committee used multiple databases to obtain publications from a large, comprehensive search. Most of the studies identified had large prospective cohorts, but smaller sample sizes were also included. Therefore, risk of publication bias was low. Details regarding how the NESR accounted for publication bias have been described elsewhere.^13^ Primary and secondary analyses were included from the PREDIMED (Prevención con Dieta Mediterránea) trial, which reported randomization issues.^171^ However, PREDIMED investigators republished the data after reanalysis and reconfirmed the initial findings.^18^

## Discussion

Despite the different approaches, study designs, dietary assessment methods, geographical regions, and dietary pattern labels, the evidence demonstrated that dietary patterns associated with lower ACM risk were consistently characterized by higher intake of vegetables; legumes; fruits; nuts; either whole grains, cereals, or nonrefined grains; fish; and unsaturated vegetable oils. These patterns were also characterized by lower or no consumption of animal products (red and processed meat, meat and meat products, and high-fat dairy products), refined grains, and sweets (ie, higher in added sugars). Labels that were assigned to the dietary patterns varied widely (eg, Mediterranean, prudent, Healthy Eating Index, DASH, and plant-based), highlighting that high-quality diets with nutrient-dense foods are associated with better health, regardless of diet type or dietary pattern name. Although we believe this systematic review is the most comprehensive examination of US dietary patterns and ACM that is currently available, its findings align with the results of previous meta-analyses that focused on select diet types and/or cause-specific mortality.^9,10,11,12,172^

This systematic review included the most suitable study designs available to answer the question. Given the nature of the outcome (ACM), expense, duration, and power necessary for an experimental study design to explore this question, it is unlikely that many RCTs or non-RCTs would be available (funded, conducted, or published) for consideration. Studies that were included for this review were well designed and conducted using rigorous methods. Despite the preponderance of evidence from observational studies, this review presented a conclusion statement that was assigned a grade of strong, which was in accordance with studies at generally low or moderate risks of bias and with high consistency in direction and magnitude of findings, precision, directness, and generalizability. The methods used for grading the evidence underlying this conclusion statement align with other grading approaches^173,174,175^ and ensure that strengths and weaknesses in study design as well as each grading element were considered.^176^

The findings of this review are further supported by a central tenet behind other works of the 2020 committee: a nutrient-dense dietary pattern can minimize the risk of multiple diet-related chronic diseases, such as CVD, obesity, diabetes, and some cancers, and can support bone and neurocognitive health.^176,177^ For women who are pregnant, a similar healthful dietary pattern has been associated with lower risk of poor maternal-fetal outcomes.^178^ Achieving a healthy dietary pattern at each life stage should also support health in subsequent life stages. The core elements of these dietary patterns across the committee’s reviews reflect higher diet quality, are appropriate to consume across the lifespan, and have the potential to substantially minimize chronic disease risk and mortality risk.

Studies in this review suggested that dietary patterns containing nutrient-dense foods and alcoholic beverages that were within a given threshold or at low to moderate levels were associated with lower risk of ACM. However, the preponderance of this evidence varied in the methodological assessment of alcohol intake and scoring procedures within the context of the dietary patterns that were assessed. The committee conducted a separate systematic review^179^ to answer the question, what is the association between alcoholic beverages and ACM? Briefly, the focused analysis on alcoholic beverage consumption suggested that alcohol may increase ACM risk, but only minimally at low levels of intake. The committee concluded that moderating alcohol consumption to lower levels is recommended to better protect health. Other recommendations that were incorporated into the *Dietary Guidelines for Americans*, *2020-2025* were for individuals to not start drinking, avoid heavy drinking, and proceed with caution regarding alcoholic beverage consumption because it can contribute to increased rates of liver disease^180^ and excess energy intake, which is not advisable.

Dietary patterns provide a meaningful and interpretable database on foods and food groups. This review found that multiple food choices can be made toward a healthy diet that promotes beneficial outcomes. Dietary pattern approaches are advantageous because they place an emphasis on the combination of foods and beverages that meet total energy needs and are associated with health, instead of the selection of specific nutrients or foods alone, which may be more difficult for consumers to translate into a total diet. We believe such an approach provides flexibility, allowing consumers the freedom to tailor food and food group combinations that were identified as healthful to their preferences (eg, cultural acceptability and taste) and needs (eg, cost).

### Limitations

This study has several limitations. Observing study participants over time provides valuable insights regarding risk for mortality, although many included studies examined diet at only 1 time point (ie, baseline), which may or may not reflect usual dietary patterns. Most of the available evidence on dietary patterns was derived from studies that were conducted in adults. Data on dietary patterns earlier in the life course and ACM were unavailable. However, this lack of data was likely associated with the challenge and expense of undertaking long-term RCTs and/or studies with repeated measures over time, particularly starting in childhood, that examine mortality as an outcome. As a result, longitudinal data, particularly information on dietary patterns from childhood and continuing throughout the life course, are still greatly needed. Further exploration is also needed on the factors that modify or mediate the association between dietary patterns and ACM as well as the role that selected food groups, such as meat, plays in this association. In addition, the available evidence does not identify the inadequate or excessive intake or quantify the types and amounts of foods and food groups consumed in the context of dietary patterns.

Active ongoing cohorts informed most of the findings reported herein, which showed consistency across the US and more than 20 other middle- to high-income countries. Results indicated good external validity, but future work is needed within population subgroups that could not be analyzed in this review because of the lack of reported details on individuals from racial/ethnic minority groups. The available evidence was not sufficient to assess how race/ethnicity affects the association between dietary patterns and ACM. In addition, this systematic review was conducted to identify the nature and direction of the association between dietary patterns and ACM, with an intention to consider dietary patterns regardless of label, name, or type. Because of this comprehensive approach, the methodological heterogeneity of the dietary patterns included in this review was best suited for qualitative synthesis of quantitative data. However, in the future, it may be worth exploring whether and how the magnitude of these findings could be quantified through a meta-analysis.

## Conclusions

Evidence from this systematic review by the 2020 Dietary Guidelines Advisory Committee demonstrated that nutrient-dense dietary patterns, which were characterized by higher intake of vegetables, fruits, legumes, nuts, whole grains, unsaturated vegetable oils, fish, and lean meat or poultry, when included, were associated with decreased ACM risk in broadly generalizable populations of adults and older adults. These dietary patterns included relatively lower intake of red and processed meat, high-fat dairy, and refined carbohydrates or sweets. Some of these dietary patterns also included moderate intake of alcoholic beverages. Results based on additional analyses with confounding factors generally confirmed the robustness of the main findings.

## Notes

Supplement. **eTable 1.** Description of All Dietary Patterns Examined in Relation to All-Cause Mortality Outcomes in All Included Articles **eTable 2.** Results by All Included Articles for Dietary Pattern and All-Cause Mortality (ACM) Analyses
eReferences.
