# Supplementary material for: Evaluation of Dietary Patterns and All-Cause Mortality: A Systematic Review
Source: JAMA Netw Open. 2021 Aug 31;4(8):e2122277. doi: 10.1001/jamanetworkopen.2021.22277 (PMC8408672; doi:10.1001/jamanetworkopen.2021.22277)
Supplement: Supplement. — eTable 1. Description of All Dietary Patterns Examined in Relation to All-Cause Mortality Outcomes in All Included Articles eTable 2. Results by All Included Articles for Dietary Pattern and All-Cause Mortality (ACM) Analyses eReferences. [file jamanetwopen-e2122277-s001.pdf]

Supplemental Online Content

English LK, Ard JD, Bailey RL. Evaluation of dietary patterns and all-cause mortality: a systematic review. *JAMA Netw Open*. 2021;4(8):e2122277.
doi:10.1001/jamanetworkopen.2021.22277

eTable 1. Description of All Dietary Patterns Examined in Relation to All-Cause Mortality Outcomes in All Included Articles

eTable 2. Results by All Included Articles for Dietary Pattern and All-Cause Mortality (ACM) Analyses

eReferences.

This supplemental material has been provided by the authors to give readers additional information about their work.

© 2021 English LK et al. *JAMA Network Open.*

eTable 1. Description of All Dietary Patterns Examined in Relation to All-Cause Mortality Outcomes in All Included Articles[a]

**Source (country of origin)**	**Dietary pattern approach**	**Dietary pattern examined[b]**	**Foods, food groups, or other indicators of the dietary pattern examined[c]**
Estruch et al,[1] 2018 (Spain)	A priori (RCT)	Control	Advice to reduce dietary fat
		Mediterranean diet + EVOO	Abundant olive oil, vegetables, fresh fruits and juices, legumes, fish or seafood, nuts, seeds; select white meat instead of red or processed meats; cook regularly with tomato, garlic, and onion; wine preferred (if consuming alcohol); ad libitum nuts, eggs, fish, seafood, low-fat cheese, chocolate, whole-grain cereals; add 15 L EVOO
		Mediterranean diet + nuts	Abundant olive oil, vegetables, fresh fruits and juices, legumes, fish or seafood, nuts, seeds; select white meat instead of red or processed meats; cook regularly with tomato, garlic, and onion; wine preferred (if consuming alcohol); ad libitum nuts, eggs, fish, seafood, low-fat cheese, chocolate, whole-grain cereals; add 15 g/d walnuts, 7.5 g/d almonds, and 7.5 g/d hazelnuts
Abe et al,[2] 2020 (Japan)	A priori	Japanese Diet Index	Positive: seaweeds, pickles, and green and yellow vegetables (green vegetables, carrot, pumpkin, and tomato), rice, fish (rawfish, fish boiled with soy, roast fish, boiled fish paste, and dried fish), beef and pork (beef, pork, ham, and sausage), miso soup, green tea, coffee
Akbaraly et al,[3] 2011 (United Kingdom)	A priori	AHEI 2010	Positive: vegetables (not potatoes, french fries), fruits, nuts and soy protein, cereal fiber; white to red meat ratio; PUFA to SFA ratio; negative: trans UFA; neutral: alcohol
Al Rifai et al,[4] 2018 (US)	A priori	MDS	Positive: vegetables, legumes, fruits, nuts, cereals, fish, MUFA/SFA; negative: red and processed meats, dairy products; neutral: alcohol
Atkins et al,[5] 2014 (UK)	A priori	Elderly Dietary Index	Positive: vegetables, legumes, fruits, whole grain bread, cereals (including whole and refined grains), fish, dairy, and olive oil; negative: wine; neutral: meat
Baden et al,[6] 2019 (US)	A priori	hPDI	Positive: vegetables, legumes, fruits, whole grains, sugar-sweetened and artificially sweetened beverages, sweets and desserts, tea and coffee;

© 2021 English LK et al. *JAMA Network Open.*

			negative: potatoes, refined grains, fish or seafood, meats, fruit juices, eggs, miscellaneous animal foods, dairy, animal fat
		uPDI (reverse of hPDI)	Positive: vegetables, legumes, fruits, whole grains, sugar-sweetened and artificially sweetened beverages, sweets and desserts, tea and coffee; negative: potatoes, refined grains, fish or seafood, meats, fruit juices, eggs, miscellaneous animal foods, dairy, animal fat
		PDI	Positive: vegetables, potatoes, legumes, fruits, fruit juices, nuts, whole grains, refined grains, tea and coffee, sugar-sweetened and artificially sweetened beverages, sweets and desserts; negative: fish or seafood, meats, eggs, miscellaneous animal foods, dairy, animal fat
Behrens et al,[7] 2013 (US)	A priori	aMED	Positive: vegetables (not potatoes), legumes, fruits, nuts, whole grains, fish, MUFA/SFA; negative: red and processed meat. Removed alcohol as a positive component in moderation
Bellavia et al,[8] 2016 (Sweden)	A priori	mMDS	Positive: vegetables and fruits (not potatoes and fruit juice), legumes and nuts, nonrefined high-fiber grains (whole meal bread, crisp bread, oatmeal, and bran of wheat), fish, fermented dairy products (cultured milk, yogurt, and cheese), olive oil, rapeseed oil; negative: red and processed meat; neutral: alcohol
Biesbroek et al,[9] 2017 (the Netherlands)	A priori	DASH score	Positive: vegetables (not potatoes and legumes), nuts and legumes, fruit and fruit juice, whole grains, low-fat dairy; negative: red and processed meat, sweetened beverages, sodium
		DHD15-index	Positive: vegetables, legumes, fruits, nuts, whole grains, fish, margarines, oils (replace butter, hard fats), tea, filtered coffee; negative: replace refined with whole-grain products, red meat, processed meat, alcohol, sodium; neutral: dairy products
Bittoni et al,[10] 2015 (US)	A priori	Healthy Eating Index	Positive: vegetables, fruits, grains 6-11 servings/d = 10, variety; negative: meat, milk, total fat, SFA, cholesterol; neutral: sodium
Bo et al,[11] 2016 (Italy)	A priori	MDS	Positive: vegetables, legumes, fruits, nuts, cereals, fish, MUFA/SFA; negative: red and processed meat, dairy products; neutral: alcohol
Boggs et al,[12] 2015 (US)	A priori	DASH score	Positive: vegetables (not potatoes and legumes), nuts and legumes, fruit and fruit juice, whole grains, low-fat dairy; negative: red and processed meat, sweetened beverages, sodium
	A posteriori	Prudent[d]	High intake of vegetables and fruits

© 2021 English LK et al. *JAMA Network Open.*

		Western[d]	High intake of red and processed meat and fried foods
Bonaccio et al,[13] 2018 (Italy)	A priori	MDS	Positive: vegetables, legumes, fruits, nuts, cereals, fish, MUFA/SFA; negative: red and processed meat, dairy products; neutral: alcohol
Bongard et al,[14] 2016 (France)	A priori	Programme National Nutrition Santé Guideline Score	Positive: vegetables and fruits, seafood, vegetable fat; negative: sweetened foods, soda (drink water), added fat, salt; neutral: bread, cereals, potatoes, legumes, meat and poultry, seafood, eggs, milk and dairy products, alcohol
Booth et al,[15] 2016 (US)	A priori	MDS	Positive: vegetables, legumes, fruits, nuts, cereals, fish, MUFA/SFA; negative: red and processed meat, dairy products; neutral: alcohol
Brown et al,[16] 2016 (US)	A priori	HEI	Positive: vegetables, fruits, grains 6-11 servings/d = 10, variety; negative: meat, milk, total fat, SFA, cholesterol; neutral: sodium
Buckland et al,[17] 2011 (Spain)	A priori	arMED	Positive: vegetables (not potatoes), legumes, fruits, nuts and seeds (not juices), whole grains, refined flour, pasta, rice, bread, grains, fish, olive oil; negative: total and processed meat; neutral: alcohol
Cárdenas-Fuentes et al,[18] 2019 (Spain)	A priori	MEDAS	Positive: vegetables, dishes with tomato sauce (tomato, garlic, onion, leek, and olive oil), pulses, fruits, nuts, fish, white meat over red meat, olive oil (olive oil as principal cooking fat), red wine; negative: commercial pastries, red meat or sausages, animal fat, sugar-sweetened beverages
Chan et al,[19] 2019 (Hong Kong, China)	A priori	DQI-I	Positive: vegetables, fruits, cereals, PUFA/SFA, protein, calcium, iron, vitamin C; negative component: total fat, SFA, cholesterol, sodium, empty-energy foods; neutral: carbohydrate to protein to fat ratio
		MDS	Positive: vegetables, legumes, fruits, nuts, cereals, fish, MUFA/SFA; negative: red and processed meat, dairy products; neutral: alcohol
		MIND diet	Positive: green leafy vegetables, vegetables, beans, berries, nuts, whole grains, seafood, poultry, olive oil, wine; negative: red meat, cheese, pastries and sweets, butter and stick margarine, fried/fast food
		Okinawan Diet Score	Positive: legumes, sweet potatoes, rice, wheat, barley, other grains; negative: potatoes, fruits, nuts and seeds, fish, meat (including poultry), eggs, dairy, sugars, oils, flavors and alcohol; neutral: other vegetables, pickled vegetables
	A posteriori	Vegetable-fruits pattern[d]	Data NR

© 2021 English LK et al. *JAMA Network Open.*

		Snacks-drinks-milk products' pattern[d]	Data NR
		Meat-fish pattern[d]	Data NR
Cheng et al,[20] 2018 (US)	A priori	aMED and mMDS	Positive: vegetables, legumes, fruits, nuts 1-5 by quintiles, whole grains, fish, MUFA/SFA; negative: red and processed meat; neutral: alcohol
		Evolutionary-concordance diet score	Positive: vegetables, fruit and vegetable diversity, fruits, nuts, fish, lean meat, calcium (from nondairy foods); negative: grains and starches, baked goods, red and processed meat, dairy foods, alcohol, sodium
Chrysohoou et al,[21] 2016 (Greece)	A priori	MedDietScore	Positive: vegetables, potatoes, legumes, fruits, whole grains, fish, olive oil; negative: red and processed meat, poultry, full-fat dairy, alcohol
Cuenca-García et al,[22] 2014 (US)	A priori	MDS	Positive: vegetables, legumes, fruits, nuts, cereals, fish, MUFA/SFA; negative: red and processed meat, dairy products; neutral: alcohol
		Ideal Diet Index	Positive: vegetables and fruits, legumes, nuts, seeds, whole grains, fish; negative: processed meat, added sugar, SFA, sodium
		DQI	Positive: vegetables and fruits, breads, cereal, legumes, calcium; negative: total fat, SFA, cholesterol, protein, sodium
Dai et al,[23] 2016 (US)	A priori	MQHD	Positive: vegetables, potatoes, fruits, grains, fish and shellfish, poultry, red meat, eggs, dairy products, UFA to SFA ratio, fried foods, non-fried foods, alcohol
Drake et al,[24] 2013 (Sweden)	A priori	DQI-SNR	Positive: vegetables and fruits, fish and shellfish; negative: sucrose, SFA; neutral: PUFA, dietary fiber
Ford et al,[25] 2011 (US)	A priori	HEI	Positive: vegetables, fruits, grains 6-11 servings/d = 10, variety; negative: meat, milk, total fat, SFA, cholesterol; neutral: sodium
Ford et al,[26] 2012 (US)	A priori	HEI	Positive: vegetables, fruits, grains 6-11 servings/d = 10, variety; negative: meat, milk, total fat, SFA, cholesterol; neutral: sodium
Ford et al,[27] 2014 (US)	A priori	DST based on HEI 2005	Positive: vegetables, whole fruit and juice, total and whole grains, lean proteins (chicken or turkey; fish or seafood, not fried), dietary supplement use; negative: added fats, sugars, and sweets (including alcoholic beverages), processed meats (cold cuts, hot dogs, lunch/deli meats, bacon or sausage); neutral: dairy (milk, cheese, yogurt)

© 2021 English LK et al. *JAMA Network Open.*

Fresán et al,[28] 2019 (Spain)	A priori	Modified 2015 Dietary Guidelines for Americans Index	Positive: dark green vegetables, red/orange vegetables, starchy vegetables, other vegetables, variety of vegetables and fruits, legumes, fruits, whole grains, cereals, fish and seafood, meat and eggs, low-fat dairy, lean meat products, dairy products, dietary fiber density; neutral: total fat, SFA, trans fatty acids, cholesterol, sodium; negative: added sugar, dietary fiber density, alcohol
George et al,[29] 2014 (US)	A priori	AHEI 2010	Positive: vegetables (not potatoes, french fries), fruits, legumes and nuts, whole grains, long-chain fats (EPA + DHA), PUFA; negative: red and processed meat, sugar-sweetened beverages and fruit juices, trans fatty acids, sodium; neutral: alcohol
		aMED	Positive: vegetables (not potatoes), legumes, fruits, nuts, whole grains, fish, MUFA/SFA; negative: red and processed meat; neutral: alcohol
		DASH score	Positive: vegetables (not potatoes and legumes), nuts and legumes, fruit and fruit juice, whole grains, low-fat dairy; negative: red and processed meat, sweetened beverages, sodium
		HEI 2010	Positive: total vegetables, greens and beans, total fruit, whole fruit, whole grains, seafood and plant proteins, total protein foods, dairy, fatty acids; negative: refined grains, added sugars in "empty calories," solid fats in empty calories, sodium
Harmon et al,[30] 2015 (US)	A priori	aMED	Positive: vegetables (not potatoes), legumes, fruits, nuts, whole grains, fish, MUFA/SFA; negative: red and processed meat; neutral: alcohol
		AHEI 2010	Positive: vegetables (not potatoes, french fries), fruits, legumes and nuts, whole grains, long-chain fats (EPA + DHA), PUFA; negative: red and processed meat, sugar-sweetened beverages and fruit juices, trans fatty acids, sodium; neutral: alcohol
		DASH score	Positive: vegetables (not potatoes and legumes), nuts and legumes, fruit and fruit juice, whole grains, low-fat dairy; negative: red and processed meat, sweetened beverages, sodium
		HEI 2010	Positive: total vegetables, greens and beans, total fruit, whole fruit, whole grains, seafood and plant proteins, total protein foods, dairy, fatty acids; negative: refined grains, added sugars in "empty calories," solid fats in empty calories, sodium

© 2021 English LK et al. *JAMA Network Open.*

Hashemian et al,[31] 2019 (Iran)	A priori	AHEI 2010	Positive: vegetables (not potatoes, french fries), fruits, legumes and nuts, whole grains, long-chain fats (EPA + DHA), PUFA; negative: red and processed meat, sugar-sweetened beverages and fruit juices, trans fatty acids, sodium; neutral: alcohol
		aMED	Positive: vegetables (not potatoes), legumes, fruits, nuts, whole grains, fish, MUFA/SFA; negative: red and processed meat; neutral: alcohol
		DASH score	Positive: vegetables (not potatoes and legumes), nuts and legumes, fruit and fruit juice, whole grains, low-fat dairy; negative: red and processed meat, sweetened beverages, sodium
		HEI 2015	Positive: total vegetables, greens and beans, total fruit, whole fruit, whole grains, seafood and plant proteins, total protein foods, dairy, PUFA+MUFA/SFA; negative: refined grains, added sugars, SFA, sodium
		WCRF/AICR (diet only) score	Positive: vegetables and fruits, dietary fiber; negative: red and processed meat, sugary drinks, alcohol, sodium, energy-dense foods
Haveman-Nies et al,[32] 2002 (Belgium, Denmark, Italy, the Netherlands, Portugal, Spain, and Switzerland)	A priori	Adjusted MedDietScore	Positive: vegetables and fruits, legumes, nuts, seeds, grains, MUFA/SFA; negative: red and processed meat, dairy products; neutral: alcohol
Hodge et al,[33] 2011 (Australia)	A priori	MDS	Positive: vegetables, legumes, fruits, nuts, cereals, fish, olive oil; negative: red and processed meat, dairy products; neutral: alcohol
Hodge et al,[34] 2018 (Australia)	A priori	MDS, using olive oil instead of MUFA/SFA ratio	Positive: vegetables, legumes, fruits, nuts, cereals, fish, olive oil; negative: red and processed meat, dairy products; neutral: alcohol
Hu et al,[35] 2020 (US)	A priori	AHEI 2010	Positive: vegetables (not potatoes, french fries), fruits, legumes and nuts, whole grains, long-chain fats (EPA + DHA), PUFA; negative: red and processed meat, sugar-sweetened beverages and fruit juices, trans fatty acids, sodium; neutral: alcohol
		aMED	Positive: vegetables (not potatoes), legumes, fruits, nuts, whole grains, fish, MUFA/SFA; negative: red and processed meat; neutral: alcohol
		DASH score	Positive: vegetables (not potatoes and legumes), nuts and legumes, fruit and fruit juice, whole grains, low-fat dairy; negative: red and processed meat, sweetened beverages, sodium

© 2021 English LK et al. *JAMA Network Open.*

		HEI 2015	Positive: total vegetables, greens and beans, total fruit, whole fruit, whole grains, seafood and plant proteins, total protein foods, dairy, PUFA+MUFA/SFA; negative: refined grains, added sugars, SFA, sodium
Hulsegge et al,[36] 2016 (the Netherlands)	A priori	mMDS	Positive: vegetables, legumes, fruits, cereals, fish, MUFA+PUFA/SFA; negative: meat, dairy products; neutral: alcohol
Kaluza et al,[37] 2009 (Sweden)	A priori	NRFS	Negative: white bread, sweets (combined buns/cakes and biscuits/wafers/rusks and gateau/pastries), pork, beef and veal, minced meat, hamburgers, meatballs, sausage (as main dish), meat or sausage (on sandwiches), liver and kidney, blood pudding, liver pate, cheese (28% fat), butter (80% fat), cream or crème fraiche, potato chips, popcorn, fried potatoes or french fries, mayonnaise, ice cream
		RFS	Positive: tomatoes, broccoli, spinach, mustard, turnip, collard greens, carrots or mixed vegetables and carrots, green salad, sweet potatoes, yams, other potatoes, dried beans, apples or pears, oranges, cantaloupe, orange or grapefruit juice, grapefruit, other fruit juices, dark breads (whole wheat, rye, pumpernickel), cornbread, tortillas and grits, high-fiber cereals, cooked cereals, fish (baked or broiled), chicken or turkey (baked or stewed), milk (skim, 1%, 2%)
Kaluza et al,[38] 2019 (Australia)	A priori	AIDI	Positive: vegetables and fruits, nuts, whole grain bread, breakfast cereal, low-fat cheese, olive and canola oil, red wine, tea, chocolate; negative: chips, processed meat, unprocessed meat, offal, soft drinks
Kant et al,[39] 2000 (US)	A priori	RFS	Positive: tomatoes, broccoli, spinach, mustard, turnip, collard greens, carrots or mixed vegetables and carrots, green salad, sweet potatoes, yams, other potatoes, dried beans, apples or pears, oranges, cantaloupe, orange or grapefruit juice, grapefruit, other fruit juices, dark breads (whole wheat, rye, pumpernickel), cornbread, tortillas and grits, high-fiber cereals, cooked cereals, fish (baked or broiled), chicken or turkey (baked or stewed), milk (skim, 1%, 2%)
Kant et al,[40] 2004 (US)	A priori	RFBS, a modified RFS	Positive: tomatoes, broccoli, spinach, mustard, turnip, collard greens, carrots or mixed vegetables and carrots, green salad, sweet potatoes, baked or boiled potatoes, dried beans, all fruits (apples or apple sauce, oranges, grapefruits, cantaloupes), orange or grapefruit juice, other fruit

© 2021 English LK et al. *JAMA Network Open.*

			juices, whole grains (cooked cereals such as oatmeal), high-fiber cereals; dark breads (whole wheat, rye, pumpernickel), corn tortillas and breads, fish (baked or broiled), chicken or turkey, dry beans, nuts, low-fat or non-fat dairy (skim, 1%, 2%) milk, removal of chicken skin or fat on red meat
	A posteriori	Fruit, vegetable, whole grain[d]	Emphasized fruit, vegetable, and whole grain
		Ethnic[d]	Emphasized beans, corn bread/tortillas, and mustard greens loaded on this factor
		Low-fat[d]	Emphasized skim milk and behavior-related items
		Cluster 1[d]	Less likely to mention whole grains and low-fat or skim milk, and to remove fat from meat and poultry
		Cluster 2[d]	Less likely to mention most fruits and vegetables
		Cluster 3[d]	Less likely to mention most fruits and high-fiber cereals
		Cluster 4[d]	Highest proportion reporting weekly use of most items
Kant et al,[41] 2009 (US)	A priori	DBS	Positive: vegetables, fruits, whole grains, lean meat, low-fat dairy; negative: added solid fat
Kappeler et al,[42] 2013 (US)	A priori	HEI	Positive: vegetables, fruits, grains 6-11 servings/d = 10, variety; negative: meat, milk, total fat, SFA, cholesterol; neutral: sodium
Kim et al,[43] 2013 (Korea)	A priori	Healthy diet score	Positive: vegetables and fruits, brown rice, fish; negative: sugar-sweetened beverages (coffee and soft drinks), sodium
Kim et al,[44] 2018 (US)	A priori	hPDI	Positive: vegetables, legumes, fruits, whole grains, sugar-sweetened and artificially sweetened beverages, sweets and desserts, tea and coffee
		uPDI	Negative: potatoes, refined grains, fish or seafood, meat, fruit juices, egg, miscellaneous animal foods, dairy, animal fat
		PDI	Positive: vegetables, potatoes, legumes, fruits, fruit juices, nuts, whole grains, refined grains, tea and coffee, sugar-sweetened and artificially sweetened beverages, sweets and desserts; negative: fish or seafood, meat, eggs, miscellaneous animal foods, dairy, animal fat
Kim et al,[45] 2019 (US)	A priori	hPDI	Positive: vegetables, legumes, fruits, whole grains, sugar-sweetened and artificially sweetened beverages, sweets and desserts, tea and coffee
		uPDI	Negative: potatoes, refined grains, fish or seafood, meat, fruit juices, egg, miscellaneous animal foods, dairy, animal fat

© 2021 English LK et al. *JAMA Network Open.*

		Provegetarian food pattern; Provegetarian Diet Index[d]	Positive: vegetables, potatoes, legumes, fruits, nuts, cereals, olive oil; negative: fish and other seafood, meats and meat products, eggs, dairy products, animal fats
		PDI	Positive: vegetables, potatoes, legumes, fruits, fruit juices, nuts, whole grains, refined grains, tea and coffee, sugar-sweetened and artificially sweetened beverages, sweets and desserts; negative: fish or seafood, meat, eggs, miscellaneous animal foods, dairy, animal fat
Knoops et al,[46] 2004 (Belgium, Denmark, Finland, France, Greece, Hungary, Italy, the Netherlands, Portugal, Spain, and Switzerland)	A priori	mMDS	Positive: vegetables and potatoes, legumes, fruits, nuts and seeds, grains, fish, MUFA/SFA; negative: meat and meat products, dairy products
Knoops et al,[47] 2006 (Belgium, Denmark, Finland, France, Greece, Italy, the Netherlands, Portugal, Spain, and Switzerland)	A priori	mMDS	Positive: vegetables, legumes, nuts and seeds, fruits, cereals, fish, MUFA/SFA; negative: meat and poultry, dairy products; neutral: alcohol
		MAI	Positive: vegetables, potatoes, legumes, fruits, cereals, MUFA, wine; negative: meat and poultry, eggs, milk and milk products, sugar, SFA
Kurotani et al,[48] 2016 (Japan)	A priori	Japanese Food Guide score	Positive: vegetable dishes, fruits, grain dishes, fish and meat dishes, milk; negative: alcohol and snacks, total energy intake
		Modified Japanese Food Guide score	Positive: vegetable dishes, fruits, grain dishes, fish and meat dishes; white: red meat, milk; negative: alcohol and snacks, total energy intake
Kurotani et al,[49] 2019 (Japan)	A priori	Japanese Food Guide, according to Japanese ADI	Positive: vegetable dishes, fruits, grain dishes, fish and meat dishes, milk; negative: alcohol and snacks, total energy intake
Lagiou et al,[50] 2006 (Sweden)	A priori	MDS	Positive: vegetables, legumes, fruits, nuts, cereals, fish, MUFA/SFA; negative: red and processed meat, dairy products; neutral: alcohol
Lasheras et al,[51] 2000 (Spain)	A priori	mMDS	Positive: vegetables, legumes, fruits, cereals (including breads, potatoes), MUFA/SFA; negative: meat and meat products, milk and dairy products; neutral: alcohol

© 2021 English LK et al. *JAMA Network Open.*

Lassale et al,[52] 2016 (Denmark, France, Germany, Greece, Italy, the Netherlands, Norway, Spain, Sweden, and UK)	A priori	DASH score	Positive: vegetables (not potatoes and legumes), nuts and legumes, fruit and fruit juice, whole grains, low-fat dairy; negative: red and processed meat, sweetened beverages, sodium
		MDS	Positive: vegetables, legumes, fruits, nuts, cereals, fish, MUFA/SFA; negative: red and processed meat, dairy products; neutral: alcohol
		rMED	Positive: vegetables (not potatoes), legumes, fruits, nuts, and Seeds (not juice), whole grains, refined flour, pasta, rice, bread, grains, fish, olive oil; negative: total and processed meat; neutral: alcohol
		MSDPS	Positive: vegetables, potatoes and other starchy foods, legumes, olives, nuts, fruits, whole grains, fish and other seafood, poultry, eggs, meat, dairy, sweets, olive oil, wine
		DQI-I	Positive: vegetables, fruits, cereals, PUFA/SFA, protein, calcium, iron, vitamin C; negative component: total fat, SFA, cholesterol, sodium, empty-energy foods; neutral: carbohydrate to protein to fat ratio
		HEI 2010	Positive: total vegetables, greens and beans, total fruit, whole fruit, whole grains, seafood and plant proteins, total protein foods, dairy, fatty acids; negative: refined grains, added sugars in “empty calories,” solid fats in empty calories, sodium
		HNFI	Positive: cabbage, root vegetables, apples and pears, rye bread, oatmeal, fish
		HLI-Diet	Positive: vegetables, fruits, fatty fish, PUFA/SFA; negative: margarine
Lim et al,[53] 2018 (Korea)	A priori	DQI-K	Positive: vegetables, fruits, whole grain; negative: sweetened beverages, total fat, SFA, cholesterol, protein, sodium
Limongi et al,[54] 2017 (Italy)	A priori	MDS	Positive: vegetables, legumes, fruits, whole grain products, fish and seafood (not breaded), poultry (not breaded), olive oil; negative: red and processed meat, eggs, sweets; neutral: milk and dairy products
Liu et al,[55] 2019 (US)	A priori	DST based on HEI 2005	Positive: vegetables, whole fruit and juice, total and whole grains, lean proteins (chicken or turkey; fish or seafood, not fried), dietary supplement use; negative: added fats, sugars, and sweets (including alcoholic beverages), processed meats (cold cuts, hot dogs, lunch/deli meats, bacon or sausage); neutral: dairy (milk, cheese, yogurt)
Loprinzi et al,[56] 2018 (US)	A priori	AHEI 2005	Positive: total vegetables, dark green/orange vegetables, legumes, total fruit, whole fruit, whole grains, total grains, meat and beans, milk,

© 2021 English LK et al. *JAMA Network Open.*

			yogurt, cheese, soy beverages, healthy oils; negative: SFA, solid fats, alcohol, added sugars, sodium
Mai et al,[57] 2005 (US)	A priori	RFS	Positive: tomatoes, broccoli, spinach, mustard, turnip, collard greens, carrots or mixed vegetables and carrots, green salad, sweet potatoes, yams, other potatoes, dried beans, apples or pears, oranges, cantaloupe, orange or grapefruit juice, grapefruit, other fruit juices, dark breads (whole wheat, rye, pumpernickel), cornbread, tortillas and grits, high-fiber cereals, cooked cereals, fish (baked or broiled), chicken or turkey (baked or stewed), milk (skim, 1%, 2%)
Martínez-Gómez et al,[58] 2013 (Spain)	A priori	Healthy diet score	Positive: vegetables, fruits, whole grains, fish, vegetable fats; negative: red and processed meat, animal fats
Martínez-González et al,[59] 2012 (Spain)	A priori	MDS	Positive: vegetables, legumes, fruits, nuts, cereals, fish, MUFA/SFA; negative: red and processed meat, dairy products; neutral: alcohol
Martínez-González et al,[60] 2014 (Spain)	A priori	Provegetarian food pattern	Positive: vegetables, potatoes, legumes, fruits, nuts, cereal, olive oil; negative: fish and other seafood, meats and meat products, eggs, dairy products, animal fat
McCullough et al,[61] 2011 (US)	A priori	Healthy diet score	Positive: vegetables and fruits, fruit and vegetable variety, fruit, whole grains; negative: red and processed meat
McNaughton et al,[62] 2012 (UK)	A priori	MDS	Positive: vegetables, legumes, fruits, nuts, cereals, fish, MUFA/SFA; negative: red and processed meat, dairy products; neutral: alcohol
		RFS	Positive: tomatoes, broccoli, spinach, mustard, turnip, collard greens, carrots or mixed vegetables and carrots, green salad, sweet potatoes, yams, other potatoes, dried beans, apples or pears, oranges, cantaloupe, orange or grapefruit juice, grapefruit, other fruit juices, dark breads (whole wheat, rye, pumpernickel), cornbread, tortillas and grits, high-fiber cereals, cooked cereals, fish (baked or broiled), chicken or turkey (baked or stewed), milk (skim, 1%, 2%)
		Healthy diet score	Positive: vegetables and fruits, pulses and nuts, fish, dietary fiber, calcium; negative: red and meat products, total nonmilk extrinsic sugars, SFA, cholesterol; neutral: PUFA, carbohydrates, protein
Menotti et al,[63] 2012 (Italy)	A priori	MAI	Positive: vegetables, legumes, fruit (fresh and dry), cereals, fish, virgin olive oil, wine; negative: cakes, pies, cookies, sugar, meat and poultry, eggs, milk, cheese, sweet beverages, animal fats and margarines

© 2021 English LK et al. *JAMA Network Open.*

Menotti et al,[64] 2017 (Croatia, Finland, Greece, Italy, Japan, the Netherlands, Serbia, and US)	A priori	MAI; wine was modified to all alcoholic beverages	Positive: vegetables, potatoes, legumes, fruits, cereals, MUFA, alcohol; negative: meat and poultry, eggs, milk and milk products; sugar, SFA
Michels and Wolk,[65] 2002 (Sweden)	A priori	NRFS	Negative: white bread, sweets (combined buns/cakes and biscuits/wafers/rusks and gateau/pastries), pork, beef and veal, minced meat, hamburgers, meatballs, sausage (as main dish), meat or sausage (on sandwiches), liver and kidney, blood pudding, liver pate, cheese (28% fat), butter (80% fat), cream or crème fraiche, potato chips, popcorn, fried potatoes or french fries, mayonnaise, ice cream
		RFS	Positive: tomatoes, broccoli, spinach, mustard, turnip, collard greens, carrots or mixed vegetables and carrots, green salad, sweet potatoes, yams, other potatoes, dried beans, apples or pears, oranges, cantaloupe, orange or grapefruit juice, grapefruit, other fruit juices, dark breads (whole wheat, rye, pumpernickel), cornbread, tortillas and grits, high-fiber cereals, cooked cereals, fish (baked or broiled), chicken or turkey (baked or stewed), milk (skim, 1%, 2%)
Mitrou et al,[66] 2007 (US)	A priori	MDS	Positive: vegetables, legumes, fruits, nuts, cereals, fish, MUFA/SFA; negative: red and processed meat, dairy products; neutral: alcohol
		tMED	Positive: vegetables (not potatoes), legumes, fruits, nuts, whole grains, fish, MUFA/SFA; negative: red and processed meat, dairy products; neutral: alcohol
Mokhtari et al,[67] 2019 (Iran)	A priori	DASH score	Positive: vegetables (not potatoes and legumes), nuts and legumes, fruit and fruit juice, whole grains, low-fat dairy; negative: red and processed meat, sweetened beverages, sodium
Muller et al,[68] 2016 (Denmark, France, Germany, Greece, Italy, the Netherlands, Norway, Spain, Sweden, and UK)	A priori	WCRF/AICR score (diet only)	Positive: vegetables and fruits, dietary fiber; negative: red and processed meat, sugary drinks, alcohol, sodium, energy-dense foods
Mursu et al,[69] 2013 (US)		AHEI 2010	Positive: vegetables (not potatoes, french fries), fruits, legumes and nuts, whole grains, long-chain fats (EPA + DHA), PUFA; negative: red and

© 2021 English LK et al. *JAMA Network Open.*

			processed meat, sugar-sweetened beverages and fruit juices, trans fatty acids, sodium; neutral: alcohol
	A priori	Diet quality score	Positive: green vegetables, other vegetables, tomatoes, legumes, beans, soy products, fruits, nuts, seeds, whole grains, fish, poultry, low-fat dairy, oil, tea, coffee, beer, wine, liquor; negative: fried potatoes, red meat, liver, processed meat, butter, whole-fat dairy, soft drinks, sweets, salty snacks, fried foods; neutral: potatoes, fruit juices, chocolate, diet soft drinks, eggs, margarine
Nakamura et al,[70] 2009 (Japan)	A priori	Reduced-salt Japanese diet score	Positive: tsukemono (pickled vegetables), fish; negative: noodles, eggs, meat; neutral: occasional drinking
Neelakantan et al,[71] 2018 (Singapore)	A priori	aMED	Positive: vegetables (not potatoes), legumes, fruits, nuts, whole grains, fish, MUFA/SFA; negative: red and processed meat; neutral: alcohol
		DASH score	Positive: vegetables (not potatoes and legumes), nuts and legumes, fruit and fruit juice, whole grains, low-fat dairy; negative: red and processed meat, sweetened beverages, sodium
		AHEI	Positive: vegetables (not potatoes, french fries), fruits, legumes and nuts, whole grains, long-chain fats (EPA + DHA), PUFA; negative: red and processed meat, sugar-sweetened beverages and fruit juices, trans fatty acids, sodium; neutral: alcohol
Nilsson et al,[72] 2012 (Sweden)	A priori	Traditional Sami diet score	Positive: berries, fatty fish, red meat, total fat; negative: bread, fiber
Oba et al,[73] 2009 (Japan)	A priori	Japanese Food Guide Spinning Top score	Positive: vegetable dishes, fruit, grain dishes, fish and meat dishes, milk, alcohol, energy from snacks
Okada et al,[74] 2018 (Japan)	A priori	Japan food score	Positive: vegetables (spinach or garland chrysanthemum, carrots or pumpkin, tomatoes, cabbage or head lettuce and Chinese cabbage), Japanese pickles, fungi, seaweeds, beans and bean products (boiled beans and tofu), fruits, fish (fresh)
Olsen et al,[75] 2011 (Denmark)	A priori	HNFI	Positive: cabbage, root vegetables, apples and pears, rye bread, oatmeal, fish

© 2021 English LK et al. *JAMA Network Open.*

Osler et al,[76] 2001 (Denmark)	A priori	Healthy food index[d]	Positive: Not consuming butter, lard, or margarine daily; consuming raw or boiled vegetables at least once daily; consuming either coarse white or coarse rye bread at least once daily and/or fruit at least once daily
	A posteriori	Prudent[d]	Wholemeal bread (and inversely with other types), pasta, rice, oatmeal products, fruits, vegetables, and fish
		Western[d]	High intake of meats, sausages, potatoes, butter, and white bread
Panizza et al,[77] 2018 (US)	A priori	HEI 2015	Positive: total vegetables, greens and beans, total fruit, whole fruit, whole grains, seafood and plant proteins, total protein foods, dairy, PUFA+MUFA/SFA; negative: refined grains, added sugars, SFA, sodium
Park, Steck, Fung, et al,[78] 2016 (US)	A priori	MedDietScore	Positive: vegetables, potatoes, legumes, fruits, whole grains, fish, olive oil; negative: red and processed meat, poultry, full-fat dairy, alcohol
Park, Fung, Steck, et al,[79] 2016 (US)	A priori	HEI	Positive: vegetables, fruits, grains 6-11 servings/d = 10, variety; negative: meat, milk, total fat, SFA, cholesterol; neutral: sodium
Prinelli et al,[80] 2015 (Italy)	A priori	MedDietScore	Positive: vegetables, potatoes, legumes, fruits, whole grains, fish, olive oil; negative: red and processed meat, poultry, full-fat dairy, alcohol
		MDS	Positive: vegetables, legumes, fruits, nuts, cereals, fish, MUFA/SFA; negative: red and processed meat, dairy products; neutral: alcohol
Reedy et al,[81] 2014 (US)	A priori	AHEI 2010	Positive: vegetables (not potatoes, french fries), fruits, legumes and nuts, whole grains, long-chain fats (EPA + DHA), PUFA; negative: red and processed meat, sugar-sweetened beverages and fruit juices, trans fatty acids, sodium; neutral: alcohol
		aMED	Positive: vegetables (not potatoes), legumes, fruits, nuts, whole grains, fish, MUFA/SFA; negative: red and processed meat; neutral: alcohol
		DASH score	Positive: vegetables (not potatoes and legumes), nuts and legumes, fruit and fruit juice, whole grains, low-fat dairy; negative: red and processed meat, sweetened beverages, sodium
		HEI 2010	Positive: total vegetables, greens and beans, total fruit, whole fruit, whole grains, seafood and plant proteins, total protein foods, dairy, fatty acids; negative: refined grains, added sugars in “empty calories,” solid fats in empty calories, sodium
Roswall et al,[82] 2015 (Sweden)	A priori	HNFI	Positive: cabbage, root vegetables, apples and pears, rye bread, oatmeal, fish

© 2021 English LK et al. *JAMA Network Open.*

Seymour et al,[83] 2003 (US)	A priori	DQI	Positive: vegetables and fruits, breads, cereals, legumes, calcium; negative: total fat, SFA, cholesterol, protein, sodium
Shah et al,[84] 2018 (US)	A priori	MDS	Positive: vegetables, legumes, fruits, nuts, cereals, fish, MUFA/SFA; negative: red and processed meat, dairy products; neutral: alcohol
		DASH score	Positive: vegetables (not potatoes and legumes), nuts and legumes, fruit and fruit juice, whole grains, low-fat dairy; negative: red and processed meat, sweetened beverages, sodium
Shahar et al,[85] 2009 (US)	A priori	HEI	Positive: vegetables, fruits, grains 6-11 servings/d = 10, variety; negative: meat, milk, total fat, SFA, cholesterol; neutral: sodium
Shivappa et al,[86] 2017 (UK)		AHEI 2010	Positive: vegetables (not potatoes, french fries), fruits, legumes and nuts, whole grains, long-chain fats (EPA + DHA), PUFA; negative: red and processed meat, sugar-sweetened beverages and fruit juices, trans fatty acids, sodium; neutral: alcohol
Shvetsov et al,[87] 2016 (US)		aMED, Q5 vs Q1	Positive: vegetables (not potatoes), legumes, fruits, nuts, whole grains, fish, MUFA/SFA; negative: red and processed meat; neutral: alcohol
Sijtsma et al,[88] 2015 (the Netherlands)	A priori	DHNaFS	Positive: vegetables, potatoes, legumes (protein-rich plant foods), fruit, whole grains, fish, lean meat, eggs, low-fat milk and yogurt, vegetable oils and soft margarines, noncaloric drinks (tea, coffee, water)
		DUNaFS	Positive: processed vegetables, refined grains, high-fat meat, processed meat, full-fat milk, cheese, fruit juice and sugar-sweetened beverages, butter and hard margarines, ready meals and soups, spreads and snacks
Sjögren et al,[89] 2010 (Sweden)	A priori	mMDS	Positive: vegetables and legumes, fruits, cereals and potatoes, fish, PUFA/SFA, alcohol; negative: meat and meat products, milk and milk products
Sotos-Prieto et al,[90] 2017 (US)	A priori	aMED	Positive: vegetables (not potatoes), legumes, fruits, nuts, whole grains, fish, MUFA/SFA; negative: red and processed meat; neutral: alcohol
		DASH score	Positive: vegetables (not potatoes and legumes), nuts and legumes, fruit and fruit juice, whole grains, low-fat dairy; negative: red and processed meat, sweetened beverages, sodium
		AHEI	Positive: vegetables (not potatoes, french fries), fruits, legumes and nuts, whole grains, long-chain fats (EPA + DHA), PUFA; negative: red and processed meat, sugar-sweetened beverages and fruit juices, trans fatty acids, sodium; neutral: alcohol

© 2021 English LK et al. *JAMA Network Open.*

Stefler et al,[91] 2017 (Poland, Russian Federation, and Czech Republic)	A priori	Revised MDS	Positive: vegetables, legumes, fruits, nuts, cereals, fish, olive oil; negative: meat and meat products, dairy products; neutral: alcohol
		mMDS	Positive: vegetables, legumes, fruits, cereals, fish, MUFA+PUFA/SFA; negative: meat, dairy products; neutral: alcohol
Struijk et al,[92] 2014 (the Netherlands)	A priori	mMDS adherence	Positive: vegetables, legumes and nuts, fruits, grains, fish and seafood, MUFA+PUFA to SFA ratio; negative: meat, dairy; neutral: alcohol
		DHD-I	Scored from 0 to 10: vegetables, fruit, fiber, fish, SFA, trans fatty acids, salt, alcohol
	A posteriori	Prudent[d]	High intake of fish and shellfish, raw vegetables, wine, and high-fiber cereals
		Western[d]	High intake of french fries, fast food, low-fiber products, alcoholic drinks (except wine), and sugar-sweetened drinks
Thorpe et al,[93] 2013 (US)	A priori	HEI	Positive: vegetables, fruits, grains 6-11 servings/d = 10, variety; negative: meat, milk, total fat, SFA, cholesterol; neutral: sodium
Tognon et al,[94] 2011 (Sweden)	A priori	mMDS (refined MDS)	Positive: vegetables and potatoes, legumes, nuts, seeds, fruit and fresh juices, whole grain cereals, fish and fish products, MUFA+PUFA/SFA, alcohol; negative: meat, meat products, eggs, dairy products
		mMDS, aMED (HALE)	Positive: vegetables and potatoes, legumes, nuts, seeds, fruit and fresh juices, cereals, fish and fish products, MUFA/SFA; negative: meat, meat products, eggs, dairy products
Tognon et al,[95] 2012 (Sweden)	A priori	mMDS, refined	Positive: vegetables and potatoes, legumes, nuts, seeds, fruit and fresh juices, whole grain cereals, fish and fish products, MUFA+PUFA/SFA, alcohol; negative: meat, meat products, eggs, dairy products
Tognon et al,[96] 2014 (Denmark)	A priori	mMDS	Positive: vegetables, fruits, cereals, fish and fish products, MUFA+PUFA/SFA, alcohol; negative: meat, meat products, eggs, dairy products
Tong et al,[97] 2016 (UK)	A priori	Literature-based MDS	Positive: vegetables, legumes, fruits, nuts, cereals, fish, olive oil; negative: meat, dairy products; neutral: alcohol
		mMDS	Positive: vegetables (not potatoes), legumes, fruits, nuts, seeds (not juice), whole grains, refined flour, pasta, rice, bread, grains, fish, olive oil; negative: total and processed meat; neutral: alcohol

© 2021 English LK et al. *JAMA Network Open.*

		PyrMDS	Positive: vegetables, legumes, fruits, nuts 1-2/d or 0/d, cereals, fish, white meat, eggs, dairy, olive oil; negative: potatoes, red meat, processed meat, sweets, alcohol
		Tertiles of the MDS	Positive: vegetables, legumes, fruits, cereals, fish, olive oil; negative: meat, dairy products; neutral: alcohol
Trichopoulou et al,[98] 2003 (Greece)	A priori	MDS	Positive: vegetables, legumes, fruits, nuts, cereals, fish, MUFA/SFA; negative: red and processed meat, dairy products; neutral: alcohol
Trichopoulou et al,[99] 2005 (Denmark, France, Germany, Greece, Italy, the Netherlands, Spain, Sweden, and UK)	A priori	mMDS	Positive: vegetables (not potatoes), legumes, fruits, nuts, and Seeds (not juice), whole grains, refined flour, pasta, rice, bread, grains, fish, olive oil; negative: total and processed meat; neutral: alcohol
Trichopoulou et al,[100] 2009 (Greece)	A priori	MDS	Positive: vegetables, legumes, fruits, nuts, cereals, fish, MUFA/SFA; negative: red and processed meat, dairy products; neutral: alcohol
van Dam et al,[101] 2008 (US)	A priori	AHEI 2010	Positive: total vegetables, greens and beans, total fruit, whole fruit, whole grains, seafood and plant proteins, total protein foods, dairy, fatty acids (0-10); negative: refined grains, added sugars in "empty calories," solid fats in empty calories, sodium
van den Brandt,[102] 2011 (the Netherlands)	A priori	aMED	Positive: vegetables (not potatoes), legumes, fruits, nuts, whole grains, fish, MUFA/SFA; negative: red and processed meat; neutral: alcohol
van Lee et al,[103] 2016 (the Netherlands)	A priori	DHD-I	Positive: vegetables, fruits, fish, dietary fiber; negative: SFA, trans fatty acids, alcohol, sodium
Voortman et al,[104] 2017 (the Netherlands)	A priori	Dutch Dietary Guidelines score	Positive: vegetables, legumes, fruits, nuts, whole grains, fish, dairy products, UFA and oils, tea; negative: replace refined with whole-grain products, red meat, processed meat, alcohol, sodium
Vormund et al,[105] 2015 (Switzerland)	A priori	mMDS[d]	Positive: vegetables, salad, fruits, nuts, whole grains, fish, MUFA/SFA; negative: red and processed meat, dairy products; neutral: alcohol
		aMED, mMDS	Positive: vegetables, salad, fruits, nuts, whole grains, fish, MUFA/SFA, dairy products; negative: red and processed meat; neutral: alcohol
		mMDS	Positive: vegetables, salad, fruits, nuts, whole grains, fish, MUFA/SFA; negative: red and processed meat; neutral: alcohol

© 2021 English LK et al. *JAMA Network Open.*

Wahlqvist et al,[106] 2005 (Australia, Greece, Japan, and Sweden)	A priori	MDS	Positive: vegetables, legumes, fruits, nuts, cereals, fish, MUFA/SFA; negative: red and processed meat, dairy products; neutral: alcohol
Warensjö Lemming et al,[107] 2018 (Sweden)	A priori	aMED	Positive: vegetables and fruits, legumes and nuts, nonrefined, high-fiber grains, fish, fermented dairy products, olive oil, rapeseed oil; negative: red and processed meat; neutral: alcohol
		HNFI	Positive: cabbage, root vegetables, apples and pears, rye bread, oatmeal, fish
Whalen et al,[108] 2017 (US)	A priori	MedDietScore	Positive: vegetables, fruits, nuts (1-5 by quintiles), fish, lean meat (poultry, lean beef); negative: red and processed meat, sodium; neutral: grains and starches, dairy foods, alcohol
		Paleolithic diet score	Positive: vegetables, fruit and vegetable diversity; fruits, nuts, fish, lean meat, calcium (from nondairy foods; negative: grains and starches, baked goods, red and processed meat, dairy foods, alcohol, sodium
Yu et al,[109] 2015 (US)	A priori	HEI 2010	Positive: total vegetables, greens and beans, total fruit, whole fruit, whole grains, seafood and plant proteins, total protein foods, dairy, fatty acids (0-10); negative: refined grains, added sugars in "empty calories," solid fats in empty calories, sodium
Zaslavsky et al,[110] 2017 (US)	A priori	DASH score	Positive: vegetables (not potatoes and legumes), nuts and legumes, fruit and fruit juice, whole grains, low-fat dairy; negative: red and processed meat, sweetened beverages, sodium
		aMED,[d] MDS	Positive: vegetables, legumes, fruits, nuts, cereals, fish, MUFA/SFA; negative: red and processed meat, dairy products; neutral: alcohol
Zaslavsky et al,[111] 2018 (Sweden)	A priori	aMED	Positive: vegetables (not potatoes), legumes, fruits, nuts, whole grains, fish, MUFA/SFA; negative: red and processed meat; neutral: alcohol
Anderson et al,[112] 2011 (US)	A posteriori	Healthy foods[d]	Higher intake of low-fat dairy products, fruit, whole grains, poultry, fish and vegetables; lower consumption of meat, fried foods, sweets, high-energy drinks, and added fat
		High-fat dairy products[d]	Higher intake of foods such as ice cream, cheese, and 2% and whole milk and yogurt; lower intake of poultry, low-fat dairy products, rice, and pasta

© 2021 English LK et al. *JAMA Network Open.*

		Meat, fried foods, and alcohol[d]	NR: higher intake of meat, fried poultry, beer, liquor, rice, pasta, and mixed dishes, snacks, nuts, high-energy-density drinks, mayonnaise and salad dressing
		Breakfast cereal[d]	NR: higher intake of cold breakfast cereal, fiber/bran and other cold breakfast cereal; lower intake of dark yellow vegetables, refined grains, and nuts
		Refined grains[d]	NR: higher intake of processed meat; lower intake of liquor, whole grains, cold breakfast cereal, fiber/bran and other cold breakfast cereal
		Sweets and desserts[d]	Higher intake of doughnuts, cake, cookies, pudding, chocolate, and candy; lower intake of fruit, fish, other seafood, and dark green vegetables
Atkins et al,[113] 2016 (UK)	A posteriori	High-fat/low-fiber[d]	High in red meat, meat products, white bread, fried potato, and eggs
		Prudent[d]	High in poultry, fish, fruits, vegetables, legumes, pasta, rice, wholemeal bread, eggs, and olive oil
		High sugar[d]	High in biscuits, puddings, chocolates, sweets, sweet spreads, breakfast cereals
Bamia et al,[114] 2007 (Denmark, France, Germany, Greece, Italy, the Netherlands, Norway, Spain, Sweden, and UK)	A posteriori	Plant-based[d]	Higher plant foods such as vegetables and vegetable oils, fruits, pasta, rice, other grains and legumes; poor in potatoes, margarines, and nonalcoholic beverages
Brunner et al,[115] 2008 (UK)	A posteriori	Unhealthy[d]	Higher-than-average consumption of meat and sausages, white bread, fries, and full-cream milk; average consumption of wine and beer; very low consumption of fruit and vegetables
		Sweets[d]	Higher-than-average consumption of biscuits, cakes, meat, sausages and savory pies, white bread, full-cream milk, butter, and wine and beer; average intake of fruit and vegetables
		Mediterranean-like[d]	Higher-than-average consumption of wholemeal bread, fruits, vegetables, pasta and rice, and wine and beer; low intake of full-cream milk but high intake of butter; average consumption of white bread

© 2021 English LK et al. *JAMA Network Open.*

		Healthy[d]	Higher-than-average consumption of wholemeal bread, fruits and vegetables, and polyunsaturated margarine; average to low consumption of red meat, sweet foods, and wine and beer
Granic et al,[116] 2013 (Sweden)	A posteriori	Moderate intake and starch diet[d]	Medium intake of all foods: beef, pork, sausage, egg and egg dishes, fish and seafood, fruits and vegetables, potatoes, sweets, and milk; except for high intake of flour-based foods, pastries, and sandwiches
		Moderate intake with low flour-based food diet[d] [reference]	Moderate consumption of 8 food items: beef, pork, sausage, egg and egg dishes, fish and seafood, fruits and vegetables, potatoes, coffee cake and pastries, sweets, sandwich, and milk; minimal intake of flour-based dishes, low in refined starch
		Meat and starch diet[d]	Higher consumption of potatoes, milk, sandwiches, pork and sausage-based dishes
		Low meat intake diet[d]	Lower intake of 8 food groups, including meat-based, egg-based, and potato-based dishes
Hamer et al,[117] 2010 (UK)	A posteriori	Mediterranean[d]	High consumption of fruits and raw vegetables, oily fish, coffee, and wine
		Health aware[d]	High consumption of low-fat/high-fiber foods, such as boiled potatoes, green vegetables, and wholemeal bread
		Traditional[d]	High consumption of white bread, eggs, bacon, and ham
		Sweet and fat[d]	High consumption of butter, whole milk, preserves, cream, buns, cakes, puddings, and pastries
Heidemann et al,[118] 2008 (US)	A posteriori	Prudent[d]	High consumption of vegetables, fruit, legumes, fish, poultry, and whole grains
		Western[d]	High consumption of red meat, processed meat, refined grains, french fries, and sweets and desserts
Hoffmann et al,[119] 2005 (Germany)	A posteriori	PCA pattern 1	Higher in potatoes, vegetables, legumes, bread, all types of meat, eggs, sauces, and soups
		PCA pattern 2	Higher in vegetables, fruits, dairy products, other cereals, vegetable oils, nonalcoholic beverages; lower in alcoholic beverages other than wine
		RRR pattern 1	Higher in meat, butter, sauces, and eggs; lower in bread and fruits
		RRR pattern 2	Higher in legumes, poultry, fish, and margarine; lower in butter, sugar, and cakes

© 2021 English LK et al. *JAMA Network Open.*

Hsiao et al,[120] 2013 (US)	A posteriori	Sweets and dairy[d]	High consumption of baked goods, milk, sweetened coffee and tea, and dairy-based dessert food groups; lower intake of poultry
		Western[d]	High consumption of bread, eggs, fats, fried vegetables, miscellaneous (sauces, condiments, etc.), alcohol, and soft drinks; lower intake of milk and whole fruit
		Health conscious[d]	High consumption of pasta, noodles, rice, whole fruit, poultry, nuts, fish, and vegetables; lower intake of fried vegetables, processed meats, and soft drinks
Krieger et al,[121] 2018 (Switzerland)	A posteriori	Sausage and vegetables[d]	High consumption of sausages and cooked vegetables and overall low dietary variety
		Meat and salad[d]	High consumption of meat and salad and overall low dietary variety
		Fish[d]	High consumption of fish and absence of meat-based products
		Traditional[d]	High consumption of dairy products, eggs, chocolate, dark bread, and sausages with overall high dietary variety
		High-fiber foods[d]	High consumption of yogurt, salad, vegetables, fruits, and dark bread with overall high dietary variety
Martínez-González et al,[122] 2015 (Spain)	A posteriori	Western[d]	High consumption of high-fat processed meats and red meats, alcohol, refined grains, canned fish, whole-fat dairy products, sauces, eggs, processed meals, commercial bakery products, and chocolates; lower consumption of low-fat dairy products
		Mediterranean[d]	High consumption of vegetables, EVOO, walnuts, oily fish and canned fish, fruits, other nuts, whole-wheat bread, white fish, and low-fat dairy products; low consumption of refined grains and other olive oils different from EVOO
Masala et al,[123] 2007 (Italy)	A posteriori	Prudent[d]	High consumption of cooked vegetables, legumes, fish, and seed oil
		Pasta and meat[d]	High consumption of pasta and other grains, tomato sauce, red and processed meats, added animal fat, white bread and wine; low consumption of yogurt
		Olive oil and salad[d]	High consumption of olive oil, raw vegetables (tomatoes; leafy and root vegetables), soups, and white meat such as chicken and turkey
		Sweets and dairy[d]	High consumption of added sugar, cakes, ice cream, coffee, eggs, butter, milk, and cheese
	A posteriori	Factor 1[d]	High consumption of sugar, milk, meat, fruit, pastries, and cheese

© 2021 English LK et al. *JAMA Network Open.*

Menotti et al,[124] 2012 (Italy)		Factor 2[d]	High consumption of bread, cereals, vegetables, fish, potatoes, and oils
		Factor 3[d]	High consumption of eggs and alcoholic beverages
Menotti et al,[125] 2014 (Italy)	A posteriori	Factor 2[d]	High consumption of bread, cereals, vegetables, fish, potatoes, and oils; diet score 3 [reference]
Menotti et al,[126] 2016 (Italy)	A posteriori	Factor 2,[d] Q5 vs Q1 [reference]	High consumption of bread, cereals, vegetables, fish, potatoes, and oils; adherence divided into quintiles and arbitrarily named as follows:
		Non–Mediterranean diet[d] [reference]	Q1 of Factor 2
		Prudent diet[d]	Q2, Q3, and Q4 of Factor 2
		Mediterranean diet[d]	Q5 of Factor 2
Nanri et al,[127] 2017 (Japan)	A posteriori	Prudent[d]	High consumption of vegetables; fruit; soy products; potatoes; seaweed; mushrooms; and fish, including oily fish, seafood other than fish, and fish products
		Westernized[d]	High consumption of meat, including pork and beef; processed meat; bread; dairy products; coffee; black tea; soft drinks; dressing; sauce; and mayonnaise
		Traditional Japanese[d]	High consumption of salmon, salty fish, oily fish, seafood other than fish, and pickles
Odegaard et al,[128] 2014 (Singapore)	A posteriori	Vegetable-, fruit-, and soy-rich[d]	Predominantly consisting of vegetables, fruits, and soy-based items
		Dim sum– and meat-rich[d]	Prominent contributors were a variety of foods, predominantly dim sum, fresh and processed meats and seafood, noodle and rice dishes, sweetened foods, and deep-fried foods
Waijers et al,[129] 2006 (the Netherlands)	A posteriori	Mediterranean-like[d]	High consumption of pasta and rice, sauces, fish, and vegetables in combination with vegetable oils, wine, and other cereals; potatoes, bread, and margarine contributed negatively to this component
		Traditional Dutch dinner[d]	High consumption of meat, potatoes, vegetables, eggs, and alcoholic beverages; low consumption of dairy products, sweets, and pastries
		Healthy traditional[d]	High consumption of vegetables, fruits, dairy products, potatoes, legumes, and nonalcoholic beverages; low consumption of butter and alcoholic beverages

© 2021 English LK et al. *JAMA Network Open.*

Zazpe et al,[130] 2014 (Spain)	A posteriori	Western[d]	High consumption of red meat, processed meats, potatoes, processed meals, fast food, full-fat dairy products, sauces, commercial bakery products, eggs, sugar-sweetened sodas, refined grains, and sugary products; low consumption of low-fat dairy products
		Mediterranean[d]	High consumption of vegetables, fish and seafood, fruits, olive oil, low-fat dairy products, poultry, whole-wheat bread, nuts, juices, and legumes
		Alcoholic beverages[d]	High consumption of alcohol: wine, beer, and other alcoholic beverages
Zhao et al,[131] 2019 (Japan)	A posteriori	Meat-fat pattern[d]	High consumption of oils and fats, other cereals, meat, seasoning, potatoes, sugar, and noodles
		Healthy pattern[d]	High consumption of vegetables, fruits, mushrooms, algae, seafood, beans, and seasoning
		Dairy-bread pattern[d]	High consumption of dairy products and bread; low intake of rice
Mihrshahi et al,[132] 2017 (Australia)	Other: animal products	Vegetarian[d]	Never eats any beef, lamb, pork, chicken, turkey, duck, processed meat, fish, or seafood
		Semivegetarian[d]	Eats meat ≤1 wk
		Pescovegetarian[d]	Eats fish or seafood but not beef, lamb, pork, chicken, turkey, duck, or processed meat
		Nonvegetarian (some analyses)[d]	Combined semivegetarian, pescovegetarian, and regular meat eater
		Regular meat eater[d]	Consumes meat, including fish or seafood
Song et al,[133] 2016 (US)	Other: animal products	Animal protein[d]	Major sources included processed and unprocessed red meat, poultry, dairy products, fish, and egg
		Plant protein[d]	Major sources included bread, cereals, pasta, nuts, beans, and legumes
Key et al,[134] 2009 (UK)	Other: animal products	Meat eater[d]	Eats meat
		Fish eater[d]	Does not eat meat but eats fish
		Vegetarian[d]	Does not eat meat or fish but eats dairy products or eggs or both; also analyzed combined with vegan
		Vegan[d]	Eats no animal products
		Nonvegetarian[d]	Meat eaters and fish eaters combined
		Nonvegetarian[d]	Eats nonfish meats ≥1/mo; fish and all meats ≥1/wk

© 2021 English LK et al. *JAMA Network Open.*

Orlich et al,[135] 2013 (US and Canada)	Other: animal products	Semivegetarian[d]	Eats nonfish meats ≥1/mo; all meats combined 1/mo but <1/wk
		Pescovegetarian[d]	Eats fish ≥1/mo; all other meats <1/mo
		Lacto-ovo vegetarian[d]	Eats eggs and dairy ≥1/mo; fish and all other meats <1/mo
		Vegan[d]	Eats eggs and dairy, fish, and all other meats <1/mo
Chang-Claude et al,[136] 2005 (Germany)	Other: animal products	Vegetarian[d]	Vegetarian-combined vegan: avoids meat, fish, eggs, and dairy products; lacto-ovo vegetarian: avoids meat and fish but eats eggs and/or dairy products; nonvegetarian: occasionally or regularly eats meat and/or fish
		Lacto-ovo vegetarian[d]	
		Nonvegetarian[d]	
Héroux et al,[137] 2010 (US)	Other: RRR	Response variables: unfavorable total and high-density lipoprotein cholesterol, triglyceride, glucose, blood pressure, uric acid, white blood cell, and body mass index values	Higher in processed and red meat, white potato products, non–whole grains, and added fat; lower in noncitrus fruit
Meyer et al,[138] 2011 (Germany)	Other: RRR, partial least squares regression, principal components regression	Response variables: IL-6, IL-18, and C-reactive protein	Lower intake of meat and beer; higher intake of fresh and cooked vegetables, fresh fruit, wholemeal bread, cereals and muesli, curd, nuts, sweet bread spread, and tea
Schnabel et al,[139] 2019 (France)	Other	Ultraprocessed[d]	4th level of NOVA Food Classification System
Kim et al,[140] 2019 (US)	Other	Ultraprocessed[d]	4th level of NOVA Food Classification System by quartiles of intake in times/d:

© 2021 English LK et al. *JAMA Network Open.*

			• Q1: 0 to <2.6<br>• Q2: 2.6 to <3.8<br>• Q3: 3.8 to <5.2<br>• Q4: 5.2 to <29.8
Rico-Campà et al,[141] 2019 (Spain)	Other	Ultraprocessed[d]	4th level of NOVA Food Classification System in quarters: Q1: low; Q2: low-medium; Q3: medium-high; and Q4: high

Abbreviations: ADI, area deprivation index; AHEI, alternative healthy eating index; AICR, American Institute for Cancer Research; AIDI, anti-inflammatory diet index; aMED, alternate Mediterranean diet score; arMED, adapted relative Mediterranean diet score; DASH, Dietary Approaches to Stop Hypertension; DBS, dietary behavior score; DHA, docosahexaenoic acid; DHD, Dutch Healthy Diet; DHD-I, Dutch Healthy Diet index; DHNaFS, Dutch Healthy Nutrient and Food Score; DQI, Diet Quality Index; DQI-I, DQI-International; DQI-K, DQI for Koreans; DQI-SNR, DQI–Swedish Nutrition Recommendations; DST, dietary screening tool; DUNaFS, Dutch Undesirable Nutrient and Food Score; EPA, eicosapentaenoic acid; EVOO, extra virgin olive oil; HALE, Healthy Aging: a Longitudinal Study in Europe; HEI, Healthy Eating Index; HLI, healthy lifestyle index; HNFI, Healthy Nordic Food Index; hPDI, healthful plant-based diet index; IL, interleukin; MAI, Mediterranean Adequacy Index; MDS, Mediterranean diet score; MEDAS, Mediterranean Diet Adherence Screener; MedDietScore, Mediterranean-based diet score; MIND, Mediterranean–DASH Intervention for Neurodegenerative Delay; mMDS, modified MDS; MQHD, moderation-quantified healthy diet; MSDPS, Mediterranean-style dietary pattern score; MUFA, monounsaturated fatty acids; NR, not reported; NRFS, nonrecommended food score; PCA, principal component analysis; PDI, plant-based diet index; PUFA, polyunsaturated fatty acids; PyrMDS, Mediterranean Diet Pyramid Score; Q, quintile or quartile; RCT, randomized clinical trial; RFBS, recommended food and behavior score; RFS, recommended food score; rMED, relative Mediterranean diet score; RRR, reduced rank regression; SFA, saturated fatty acids; tMED, traditional Mediterranean diet score; UFA, unsaturated fatty acids; uPDI, unhealthful PDI; WCRF, World Cancer Research Fund.

[a]Adapted from the 2020 Dietary Guidelines Advisory Committee and Nutrition Evidence Systematic Review Team.[142]

[b]The original reference for the index/score is provided when available, after the name of the dietary pattern examined in the included article.

[c]Components from the dietary patterns examined by index or score analysis were scored as positive, negative, or neutral. Neutral items, such as alcoholic beverages, are typically scored as positive within a specified threshold (eg, 0.5-1.5 servings/d for women; 1.5-2.5 servings/d for men) or in moderate amounts (eg, 10-25 g/d) as reported by the included article.

[d]The name or label of the dietary pattern was assigned by the authors of the article.

© 2021 English LK et al. *JAMA Network Open.*

eTable 2. Results by All Included Articles for Dietary Pattern and All-Cause Mortality (ACM) Analyses[a,b]

Source	No.[c]	Intervention or exposure	Subgroups	Results	Unaccounted for key confounders[d]
Estruch et al,[1] 2018	7237	Control	NA	• Incident rate/1000 persons-years, 11.7 (95% CI, 9.6-14.0)<br>• Absolute 5 y ACM risk, 5.4% (95% CI, 4.4%-6.7%)<br>• ITT analyses, 1 [Reference]	NA
		Mediterranean diet + EVOO	NA	• Incident ACM rate/1000 persons-years,10.0 (95% CI, 8.2-11.9)<br>• Absolute 5 y ACM risk, 4.4% (95% CI, 3.6%- 5.4%)<br>• ITT analyses, HR: 0.90 (95% CI, 0.69-1.18)	NA
		Mediterranean diet + nuts	NA	• Incident ACM rate/1000 persons-year, 11.2 (95% CI, 9.3-13.4)<br>• Absolute 5 y ACM risk, 5.4% (95% CI, 4.4%- 6.6%)<br>• ITT analyses, HR: 1.12 (95% CI, 0.86-1.47)	NA
Abe et al,[2] 2020	14 764	Japanese Diet Index	NA	• Q1, 1 [Reference]<br>• Q2, HR: 0.92 (95% CI, 0.85-1.00)	NA

© 2021 English LK et al. *JAMA Network Open.*

				• Q3, HR: 0.91 (95% CI, 0.83-0.99)<br>• Q4, HR: 0.91 (95% CI, 0.83-0.99); *P* for trend = .03	
Akbaraly et al,[3] 2011	7319	AHEI 2010	NA	• B (SE): −0.01 (0.00); *P* < .001	NA
Al Rifai et al,[4] 2018	1601	MDS	NA	• Q4 vs Q1-Q3, HR: 0.84 (95% CI, 0.64- 1.11)<br>• Q4 vs Q1-Q3 (accelerated failure-time model): HR: 1.09 (95% CI, 0.95-1.26)	NA
Atkins et al,[5] 2014	3133	Elderly Dietary Index	NA	• Q1, 1 [Reference]<br>• Q2, HR: 0.85 (95% CI, 0.70-1.03)<br>• Q3, HR: 0.89 (95% CI, 0.72-1.10)<br>• Q4, HR: 0.75 (95% CI, 0.60-0.94); *P* for trend = .03	NA
Baden et al,[6] 2019	47 455	hPDI	Female participants	• Q1, HR: 1.09 (95% CI, 1.03-1.16)<br>• Q2, HR: 1.00 (95% CI, 0.95-1.07)<br>• Q3, 1 [Reference]<br>• Q4, HR: 0.97 (95% CI, 0.91-1.03)<br>• Q5, HR: 0.90 (95% CI, 0.85-0.96); *P* for trend <.001	NA
			Male participants	• Q1, HR: 1.10 (95% CI, 1.02-1.19)<br>• Q2, HR: 1.04 (95% CI, 0.96-1.12)	NA

© 2021 English LK et al. *JAMA Network Open.*

				• Q3, 1 [Reference]<br>• Q4, HR: 0.92 (95% CI, 0.85-1.00)<br>• Q5, HR: 0.90 (95% CI, 0.82-0.98); *P* for trend <.001	
			Female and male participants	• Q1, HR: 1.10 (95% CI, 1.05-1.15)<br>• Q3, 1 [Reference]<br>• Q5, HR: 0.90 (95% CI, 0.85-0.95); *P* for trend <.001	NA
				• 8 y and 16 y change in hPDI, risk: 16% (95% CI, 13%-18%)<br>• Q2, HR: 1.02 (95% CI, 0.97-1.07)<br>• Q3, 1 [Reference]<br>• Q4, HR: 0.95 (95% CI, 0.90-1.00)	
			Female participants	• Q1, HR: 0.91 (95% CI, 0.85-0.98); *P* for trend <.001<br>• Q2, HR: 0.97 (95% CI, 0.91-1.03)<br>• Q3, 1 [Reference]<br>• Q4, HR: 1.07 (95% CI, 1.01-1.14)<br>• Q5, HR: 1.14 (95% CI, 1.08-1.21)	NA
			Male participants	• Q1, HR: 0.96 (95% CI, 0.88-1.05)<br>• Q2, HR: 1.05 (95% CI, 0.97-1.13)<br>• Q3, 1 [Reference]	NA

© 2021 English LK et al. *JAMA Network Open.*

				• Q4, HR: 1.03 (95% CI, 0.95-1.12)<br>• Q5, HR: 1.09 (95% CI, 1.00-1.17); P for trend = .03	
			Female and male participants	• Q1, HR: 0.93 (95% CI, 0.88-0.98); *P* for trend <.001<br>• Q2, HR: 1.00 (95% CI, 0.951.05)<br>• Q3, 1 [Reference]<br>• Q4, HR: 1.06 (95% CI, 1.01-1.11)<br>• Q5, HR: 1.12 (95% CI, 1.07-1.18)	NA
		PDI	Female participants	• Q1, HR: 1.07 (95% CI, 1.01-1.14): *P* for trend <.001<br>• Q2, HR: 1.02 (95% CI, 0.96-1.09)<br>• Q3, 1 [Reference]<br>• Q4, HR: 0.96 (95% CI, 0.91-1.02)<br>• Q5, HR: 0.95 (95% CI, 0.89-1.01)	NA
			Male participants	• Q1, HR: 1.13 (95% CI, 1.04-1.23); *P* for trend <.001<br>• Q2, HR: 1.03 (95% CI, 0.95-1.12)<br>• Q3, 1 [Reference]<br>• Q4, HR: 0.93 (95% CI, 0.85-1.01)<br>• Q5, HR: 0.96 (95% CI, 0.88-1.04)	NA

© 2021 English LK et al. *JAMA Network Open.*

			Female and male participants	• Q1, HR: 1.09 (95% CI, 1.04-1.15): *P* for trend <.001<br>• Q2, HR: 1.03 (95% CI, 0.98-1.08)<br>• Q3, 1 [Reference]<br>• Q4, HR: 0.95 (95% CI, 0.91-1.00)<br>• Q5, HR: 0.95 (95% CI, 0.90-1.00)	NA
Behrens et al,[7] 2013	170 672	aMED	Female participants	• ≤Q3, 1 [Reference]<br>• ≥Q4, RR: 0.87 (95% CI, 0.82-0.91); PAR, 9 (95% CI, 6-12)	NA
			Male participants	• ≤Q3, 1 [Reference]<br>• ≥Q4, RR: 0.85 (95% CI, 0.82-0.88); PAR, 10 (8-12)	
			Female and male participants	• ≤Q3, 1 [Reference]<br>• ≥Q4, RR: 0.86 (95% CI, 0.83-0.88); PAR, 10 (8-11)	NA
Bellavia et al,[8] 2016	71 333	mMDS	NA	• ACM:<br>• Continuous, HR: 0.96 (95% CI, 0.95-0.97)<br>• Lowest mMDS, 0-2: 1 [Reference]<br>• Middle mMDS, 3-5, HR: 0.90 (95% CI, 0.86-0.95)	Race/ethnicity

© 2021 English LK et al. *JAMA Network Open.*

				• Highest mMDS, 6-8, HR: 0.81 (95% CI, 0.75-0.86)<br>• Categorical extremes, 0 vs 8, HR: 0.71 (95% CI, 0.65-0.79)	
Biesbroek et al,[9] 2017	35 031	DASH score	Female participants	• Continuous, HR: 0.96 (95% CI, 0.92- 0.99)<br>• T1, 1 [Reference]<br>• T2, HR: 0.94 (95% CI, 0.85-1.03)<br>• T3, HR: 0.94 (95% CI, 0.86-1.03)	Race/ethnicity
			Male participants	• Continuous, male, HR: 0.92 (95% CI, 0.86-0.99)<br>• T1, 1 [Reference]<br>• T2, HR: 1.04 (95% CI, 0.89-1.22)<br>• T3, HR: 0.87 (95% CI, 0.74-1.04); *P* for trend = .15	NA
		DHD15-index	Female participants	• Continuous, female, HR: 0.92 (95% CI, 0.88-0.96)<br>• T1, 1 [Reference]<br>• T2, HR: 0.86 (95% CI, 0.78-0.93)<br>• T3, HR: 0.85 (95% CI, 0.78-0.94); P for trend = .001	NA
			Male participants	• Continuous, male, HR: 0.88 (95% CI, 0.82-0.95)	NA

© 2021 English LK et al. *JAMA Network Open.*

				• T1, 1 [Reference]<br>• T2, HR: 1.04 (95% CI, 0.88-1.21); *P* for trend = .04<br>• T3, HR: 0.84 (95% CI, 0.69-0.98); *P* for trend = .04	
Bittoni et al,[10] 2015	8950	HEI	NA	• HEI<50 vs >80; HR: 1.58 (95% CI, 1.45-1.77); *P* < .001	Alcohol intake; physical activity
Bo et al,[11] 2016	1658	MDS	NA	• Per-unit increase, at low CVD risk, HR: 0.83 (95% CI, 0.72-0.96); *P* = .01<br>• Per-unit increase, at high CVD risk (≥10), HR: 1.02 (95% CI, 0.90-1.15); *P* = .81<br>• Per-unit increase, all, HR: 0.94 (95% CI, 0.85-1.03); *P* = .20<br>• Low, 1 [Reference]<br>• Medium, HR: 0.80 (95% CI, 0.60-1.06); *P* = .12<br>• High, HR: 0.85 (95% CI, 0.54-1.35); *P* = .50	Race/ethnicity
Boggs et al,[12] 2015	37 001	DASH score	NA	• Q1, 1 [Reference]<br>• Q2, HR: 0.86 (95% CI, 0.75-1.0)<br>• Q3, HR: 0.83 (95% CI, 0.71- 0.97)<br>• Q4, HR: 0.75 (95% CI, 0.63-0.89)	NA

© 2021 English LK et al. *JAMA Network Open.*

				• Q5, HR: 0.75 (95% CI, 0.63-0.89); *P* for trend <.001	
		Prudent[e]	NA	• Q1, 1 [Reference]<br>• Q2, HR: 1.05 (95% CI, 0.90-1.23)<br>• Q3, HR: 0.92 (95% CI, 0.78-1.08)<br>• Q4, HR: 0.99 (95% CI, 0.85-1.17)<br>• Q5 HR: 1.01 (95% CI, 0.86-1.2); *P* for trend = .98	NA
		Western[e]	NA	• Q1, 1 [Reference]<br>• Q2, HR: 1.10 (95% CI, 0.93-1.29)<br>• Q3, HR: 1.16 (95% CI, 0.99-1.37)<br>• Q4, HR: 1.18 (95% CI, 1.00-1.39)<br>• Q5, HR: 1.37 (95% CI, 1.17-1.6); P for trend <.001	NA
Bonaccio et al,[13] 2018	5200	MDS	Female participants	• Poor (0-3), 1 [Reference]<br>• Average (4-6), HR: 0.88 (95% CI, 0.69-1.12)<br>• High (7-9), HR: 0.71 (95% CI, 0.42-1.17)<br>• 1-unit increase, HR: 0.95 (95% CI, 0.88-1.02)<br>• 2-unit increase, HR: 0.90 (95% CI, 0.77-1.04)	Race/ethnicity NR

© 2021 English LK et al. *JAMA Network Open.*

			Male participants	• 1-unit increase, HR: 0.94 (95% CI, 0.89-0.99)<br>• 2-unit increase, HR: 0.88 (95% CI, 0.79-0.98)<br>• Poor (0-3), 1 [Reference]<br>• Average (4-6), HR: 0.83 (95% CI, 0.69-1.01)<br>• High (7-9), HR: 0.75 (95% CI, 0.56-1.01)	NA
			Female and male participants	• Poor (0-3), 1 [Reference]<br>• Average (4-6), HR: 0.87 (95% CI, 0.75-1.01)<br>• High (7-9), HR: 0.75 (95% CI, 0.58-0.97)<br>• 1-unit increase, HR: 0.94 (95% CI, 0.90-0.98)<br>• 2-unit increase, HR (95% CI): 0.89 (95% CI, 0.81-0.97)	NA
Bongard et al,[14] 2016	960	Programme National Nutrition Santé Guideline Score	NA	• Per 1 unit increase, RR (95% CI): 0.96 (95% CI, 0.83-1.12) *P* = .63	Race/ethnicity
Booth et al,[15] 2016	5709	MDS	NA	• Q1, 1 [Reference]<br>• Q2, HR: 0.95 (95% CI, 0.73-1.22)<br>• Q3, HR: 0.7 (95% CI, 0.52-0.94)	Physical activity; anthropometry

© 2021 English LK et al. *JAMA Network Open.*

				• Q4, HR: 0.61 (95% CI, 0.46-0.82); *P* for trend <.001	
Brown et al,[16] 2016	1487	HEI	NA	• Poor, HEI: <51, 1 [Reference]<br>• Fair, HEI: 51-80, HR: 0.74 (95% CI, 0.52-1.04)<br>• Good, HEI: >80, HR: 0.70 (95% CI, 0.47-1.04); *P* for trend = .08	SES; alcohol intake
Buckland et al,[17] 2011	40 622	arMED	NA	• Continuous, HR: 0.94 (95% CI, 0.90-0.97); *P* for trend <.001<br>• Low, 1 [Reference]<br>• Medium, HR: 0.88 (95% CI, 0.79-0.99)<br>• High, HR: 0.79 (95% CI, 0.69-0.91); *P* for trend = .001	Race/ethnicity
Cárdenas-Fuentes et al,[18] 2019	7356	MEDAS	NA	• T1, 1 [Reference]<br>• T2, HR: 0.56 (95% CI, 0.45-0.70)<br>• T3, HR: 0.47 (95% CI, 0.37-0.59; *P* for trend <.001	Race/ethnicity
Chan et al,[19] 2019	2802	DQI	Female participants	• T1, 1 [Reference]<br>• T2, HR: 0.74 (95% CI, 0.58-0.96)<br>• T3, HR: 0.77 (95% CI, 0.5-1.0); *P* for trend = .04	Race/ethnicity
			Male participants	• T1, 1 [Reference]	NA

© 2021 English LK et al. *JAMA Network Open.*

				• T2, HR: 0.93 (95% CI, 0.76-1.14)<br>• T3, HR: 0.9 (95% CI, 0.73-1.10); *P* for trend = .29	
		DASH score	Female participants	• High, 1 [Reference]<br>• Low, HR: 1.18 (95% CI, 0.95-1.45)	NA
			Male participants	• High, 1 [Reference]<br>• Low, HR: 1.11 (95% CI, 0.92-1.33)	NA
		MDS; Trichopoulou, 2003	Female participants	• 0-3, HR (95% CI): 1 [Reference]<br>• 4-5, HR: 0.97 (95% CI, 0.77-1.22)<br>• 6-9, HR: 0.89 (95% CI, 0.65-1.22); *P* for trend = .48	NA
			Male participants	• 0-3, 1 [Reference]<br>• 4-5, HR: 0.86 (95% CI, 0.71-1.03)<br>• 6-9, HR: 0.96 (95% CI, 0.75-1.22); *P* for trend = .48	NA
		Mediterranean–DASH Intervention for Neurodegenerative Delay diet	Female participants	• T1, 1 [Reference]<br>• T2, HR: 0.88 (95% CI, 0.69-1.11)<br>• T3, HR: 0.84 (95% CI, 0.63-1.12); *P* for trend = .20	NA
			Male participants	• T1, 1 [Reference]	NA

© 2021 English LK et al. *JAMA Network Open.*

				• T2, HR: 0.95 (95% CI, 0.78-1.14)<br>• T3, HR: 0.85 (95% CI, 0.67-1.07); *P* for trend = .17	
		Okinawan diet score	Female participants	• T1, 1 [Reference]<br>• T2, HR: 0.72 (95% CI, 0.56-0.93)<br>• T3, HR: 0.78 (95% CI, 0.61 1.00); *P* for trend = .046	NA
			Male participants	• T1, 1 [Reference]<br>• T2, HR: 0.81 (95% CI, 0.65-1.01)<br>• T3, HR: 0.95 (95% CI, 0.78-1.16); *P* for trend = .70	NA
		Vegetable-fruits[e]	Female participants	• T1, 1 [Reference]<br>• T2, HR: 1.04 (95% CI, 0.81-1.35)<br>• T3, HR: 1.04 (95% CI, 0.8-1.36); *P* for trend = .74	NA
			Male participants	• T1, 1 [Reference]<br>• T2, HR: 0.82 (95% CI, 0.67-1.00)<br>• T3, HR: 0.86 (95% CI, 0.7-1.05); *P* for trend = .12	NA
		Snacks-drinks-milk products[e]	Female participants	• T1, 1 [Reference]<br>• T2, HR: 1.25 (95% CI, 0.97-1.6)	NA

© 2021 English LK et al. *JAMA Network Open.*

				• T3, HR: 0.83 (95% CI, 0.62-1.11); *P* for trend = .25	
			Male participants	• T1, 1 [Reference]<br>• T2, HR: 0.82 (95% CI, 0.67-1.00)<br>• T3, HR: 0.98 (95% CI, 0.79-1.20); *P* for trend = .79	NA
		Meat-fish[e]	Female participants	• T1, 1 [Reference]<br>• T2, HR: 0.94 (95% CI, 0.73-1.22)<br>• T3, HR: 1.00 (95% CI, 0.77-1.3); P for trend = .99	NA
			Male participants	• T1, 1 [Reference]<br>• T2, HR: 0.93 (95% CI, 0.76-1.14)<br>• T3, HR: 0.87 (95% CI, 0.7-1.07); *P* for trend = .17	NA
Cheng et al,[20] 2018	35 221	mMDS	NA	• Q1, 1 [Reference]<br>• Q2, HR: 0.95 (95% CI, 0.91-0.99)<br>• Q3, HR: 0.93 (95% CI, 0.89-0.98)<br>• Q4, HR: 0.91 (95% CI, 0.87-0.96)<br>• Q5, HR: 0.85 (95% CI, 0.82-0.90); *P* for trend <.01	NA
		Evolutionary-concordance diet score	NA	• Q1, 1 [Reference]<br>• Q2, HR: 0.98 (95% CI, 0.94-1.03)	NA

© 2021 English LK et al. *JAMA Network Open.*

				• Q3, HR: 0.97 (95% CI, 0.92-1.01)<br>• Q4, HR: 0.96 (95% CI, 0.91-1.01)<br>• Q5, HR: 0.95 (95% CI, 0.91-1.00); *P* for trend = .04	
Chrysohoou et al,[21] 2016	673	MedDietScore	NA	• Energy intake/100 kcal (data NR), HR: 0.92 (95% CI, 0.86-1.00)<br>• MedDietScore adherence, *P* > .30	Race/ethnicity; SES; alcohol intake
Cuenca-García et al,[22] 2014	12 449	MDS	NA	• Q1, 1 [Reference]<br>• Q2, HR: 1.17 (95% CI, 0.83-1.66)<br>• Q3, HR: 1.21 (95% CI, 0.89-1.64)<br>• Q4, HR: 1.15 (95% CI, 0.81-1.65); *P* for trend = .68	SES; anthropometry
		Ideal Diet Index	NA	• Q1, 1 [Reference]<br>• Q2, HR: 1.09 (95% CI, 0.82-1.45)<br>• Q3, HR: 1.04 (95% CI, 0.77-1.41)<br>• Q4, HR: 0.96 (95% CI, 0.68-1.34); *P* for trend = .85	NA
		DQI	NA	• Q1, 1 [Reference]<br>• Q2, HR: 1.21 (95% CI, 0.88-0.65)<br>• Q3, HR: 1.03 (95% CI, 0.75-1.42)<br>• Q4, HR: 1.24 (95% CI, 0.90-1.74); *P* for trend = .39	NA

© 2021 English LK et al. *JAMA Network Open.*

Dai et al,[23] 2016	910	Moderation-quantified healthy diet	NA	• Overall Association, HR: 0.95 (95% CI, 0.91-1.00); *P* = .03<br>• Within Pair Association, HR (95% CI): 0.96 (95% CI, 0.90 to 1.03), *P* = .24<br>• Between Pair Association, HR (95% CI): 0.95 (95% CI, 0.89 to 1.003), *P* = .07	Physical activity
Drake et al,[24] 2013	17 126	DQI-SNR	Female participants	• Predefined cutoffs:<br>• Low, 1 [Reference]<br>• Medium, HR: 1.03 (95% CI, 0.87-1.21)<br>• High, HR: 0.93 (95% CI, 0.77-1.12); *P* for trend = .36	Race/ethnicity; SES
				• Median-based cutoffs:<br>• Low, 1 [Reference]<br>• Medium, HR: 0.99 (95% CI, 0.84-1.17)<br>• High, HR: 0.92 (95% CI, 0.74-1.13); *P* for trend = .32	
				• Quintile-based cutoffs:<br>• Low, 1 [Reference]<br>• Medium, HR: 0.86 (95% CI, 0.74-0.99)	

© 2021 English LK et al. *JAMA Network Open.*

				• High, HR: 0.86 (95% CI, 0.73-1.01); *P* for trend = .18	
			Male participants	• Predefined cutoffs:<br>• Low, 1 [Reference]<br>• Medium, HR: 0.90 (95% CI, 0.78-1.03)<br>• High, HR: 0.79 (95% CI, 0.66-0.95); *P* for trend = .001	NA
				• Median-based cutoffs,<br>• Low, 1 [Reference]<br>• Medium, HR: 0.90 (95% CI, 0.78-1.04)<br>• High, HR: 0.92 (95% CI, 0.77-1.11); *P* for trend = .07	
				• Quintile-based cutoffs:<br>• Low, 1 [Reference]<br>• Medium, HR: 0.91 (95% CI, 0.80-1.04)<br>• High, HR: 0.84 (95% CI, 0.73-0.97); *P* for trend = .02	
Ford et al,[25] 2011	16 958	HEI	NA	• Healthy diet vs unhealthy diet HEI, HR: 0.85 (95% CI, 0.75-0.96); *P* < .05	Anthropometry

© 2021 English LK et al. *JAMA Network Open.*

Ford et al,[26] 2012	8375	HEI	NA	• Healthy diet vs unhealthy diet HEI, HR: 0.74 (95% CI, 0.58-0.96)	Anthropometry
Ford et al,[27] 2014	2995	DST	NA	• Healthy, DST score: >75, 1 [Reference]<br>• Unhealthy, DST score: <60, HR: 1.34 (95% CI, 0.91-1.97); *P* = .14<br>• Borderline, DST score: 60-75, HR: 1.13 (95% CI, 0.76-1.68); *P* = .39	Race/ethnicity; SES; alcohol intake
Fresán et al,[28] 2019	16 866	Modified 2015 Dietary Guidelines for Americans Index	NA	• Q1, 1 [Reference]<br>• Q2, HR: 0.92 (95% CI, 0.61-1.39)<br>• Q3, HR: 0.89 (95% CI, 0.58-1.38)<br>• Q4, HR: 0.42 (95% CI, 0.25-0.70); *P* for trend <.001	NA
George et al,[29] 2014	63 805	AHEI-2010	NA	• Q1, 1 [Reference]<br>• Q2, HR (95% CI): 0.93 (95% CI, 0.86 to 1.01)<br>• Q3, HR (95% CI): 0.90 (95% CI, 0.83 to 0.98)<br>• Q4, HR (95% CI): 0.79 (95% CI, 0.72 to 0.86)<br>• Q5, HR (95% CI): 0.82 (95% CI, 0.76 to 0.90); *P* for trend <.001	NA
		aMED	NA	• Q1, 1 [Reference]	NA

© 2021 English LK et al. *JAMA Network Open.*

				• Q2, HR: 0.87 (95% CI, 0.80-0.94)<br>• Q3, HR: 0.84 (95% CI, 0.77-0.91)<br>• Q4, HR: 0.80 (95% CI, 0.73-0.87)<br>• Q5, HR: 0.74 (95% CI, 0.68-0.81); *P* for trend <.001	
		DASH score	NA	• Q1, 1 [Reference]<br>• Q2, HR: 0.91 (95% CI, 0.83-0.99)<br>• Q3, HR: 0.86 (95% CI, 0.80-0.93)<br>• Q4, HR: 0.86 (95% CI, 0.79-0.94)<br>• Q5, HR: 0.76 (95% CI, 0.70-0.83); *P* for trend <.001	NA
		HEI 2010	NA	• Q1, 1 [Reference]<br>• Q2, HR: 0.93 (95% CI, 0.86-1.01)<br>• Q3, HR: 0.82 (95% CI, 0.75-0.89)<br>• Q4, HR: 0.84 (95% CI, 0.77-0.92)<br>• Q5, HR: 0.76 (95% CI, 0.70-0.83); *P* for trend <.001	NA
Harmon et al,[30] 2015	156 804	aMED	Female participants	• Q1, 1 [Reference]<br>• Q2, HR: 0.90 (95% CI, 0.86-0.94)<br>• Q3, HR: 0.83 (95% CI, 0.79-0.87)<br>• Q4, HR: 0.84 (95% CI, 0.79-0.88)	Alcohol intake (AHEI 2010)

© 2021 English LK et al. *JAMA Network Open.*

				• Q5, HR: 0.78 (95% CI, 0.74-0.82); *P* for trend <.001	
			Male participants	• Q1, 1 [Reference]<br>• Q2, HR: 0.92 (95% CI, 0.88-0.97)<br>• Q3, HR: 0.86 (95% CI, 0.82-0.90)<br>• Q4, HR: 0.83 (95% CI, 0.79-0.87)<br>• Q5, HR: 0.76 (95% CI, 0.73-0.80); *P* for trend <.001	NA
		AHEI 2010	Female participants	• Q1, 1 [Reference]<br>• Q2, HR: 0.94 (95% CI, 0.90-0.99)<br>• Q3, HR: 0.88 (95% CI, 0.84-0.93)<br>• Q4, HR: 0.85 (95% CI, 0.81-0.90)<br>• Q5, HR: 0.78 (95% CI, 0.74-0.82); P for trend <.001	NA
			Male participants	• Q1, 1 [Reference]<br>• Q2, HR: 0.92 (95% CI, 0.88- 0.96)<br>• Q3, HR: 0.90 (95% CI, 0.86-0.94)<br>• Q4, HR: 0.88 (95% CI, 0.84-0.93)<br>• Q5, HR: 0.78 (95% CI, 0.74-0.82); P for trend <.001	NA
		DASH score	Female participants	• Q1, 1 [Reference]<br>• Q2, HR: 0.92 (95% CI, 0.88-0.97)	NA

© 2021 English LK et al. *JAMA Network Open.*

				• Q3, HR: 0.89 (95% CI, 0.84-0.94)<br>• Q4, HR: 0.83 (95% CI, 0.79-0.87)<br>• Q5, HR: 0.80 (95% CI, 0.75-0.84); P for trend <.0001	
			Male participants	• Q1, 1 [Reference]<br>• Q2, HR: 0.95 (95% CI, 0.91-1.00)<br>• Q3, HR: 0.91 (95% CI, 0.87-0.96)<br>• Q4, HR: 0.86 (95% CI, 0.82-0.90)<br>• Q5 HR: 0.81 (95% CI, 0.77-0.85); *P* for trend <.001	NA
		HEI 2010	Female participants	• Q1, 1 [Reference]<br>• Q2, HR: 0.91 (95% CI, 0.86-0.95)<br>• Q3, HR: 0.90 (95% CI, 0.86-0.95)<br>• Q4, HR: 0.80 (95% CI, 0.76-0.84)<br>• Q5, HR: 0.79 (95% CI, 0.75-0.83); P for trend <.001	NA
			Male participants	• Q1, 1 [Reference]<br>• Q2, HR: 0.89 (95% CI, 0.85-0.93)<br>• Q3, HR: 0.85 (95% CI, 0.81-0.89)<br>• Q4, HR: 0.82 (95% CI, 0.78-0.86)<br>• Q5, HR: 0.75 (95% CI, 0.71-0.79); P for trend <.001	NA

© 2021 English LK et al. *JAMA Network Open.*

Hashemian et al,[31] 2019	42 373	AHEI 2010	NA	• Q1, 1 [Reference]<br>• Q2, HR: 0.95 (95% CI, 0.88-1.05)<br>• Q3, HR: 0.98 (95% CI, 0.89-1.07)<br>• Q4, HR: 0.94 (95% CI, 0.86-1.03)<br>• Q5, HR: 0.88 (95% CI, 0.80-0.97); *P* for trend = .01	Race/ethnicity; alcohol intake
		aMED	NA	• Q1, 1 [Reference]<br>• Q2, HR: 0.97 (95% CI, 0.90-1.05)<br>• Q3, HR: 0.87 (95% CI, 0.79-0.95)<br>• Q4, HR: 0.87 (95% CI, 0.78-0.96)<br>• Q5, HR: 0.80 (95% CI, 0.70-0.91); P for trend <.001	NA
		DASH score	NA	• Q1, 1 [Reference]<br>• Q2, HR: 0.97 (95% CI, 0.89-1.05)<br>• Q3, HR: 0.90 (95% CI, 0.82-0.98)<br>• Q4, HR: 0.92 (95% CI, 0.84-1.01)<br>• Q5, HR: 0.77 (95% CI, 0.70-0.86); *P* for trend <.001	NA
		HEI 2015	NA	• Q1, 1 [Reference]<br>• Q2, HR: 0.96 (95% CI, 0.87-1.05)<br>• Q3, HR: 1.05 (95% CI, 0.96-1.15)	NA

© 2021 English LK et al. *JAMA Network Open.*

				• Q4, HR: 0.92 (95% CI, 0.84-1.01)<br>• Q5, HR: 0.92 (95% CI, 0.83-1.01); *P* for trend = .051	
		WCRF/AICR score	NA	• Q1, 1 [Reference]<br>• Q2, HR: 0.91 (95% CI, 0.81-1.01)<br>• Q3, HR: 0.87 (95% CI, 0.78-0.98)<br>• Q4, HR: 0.87 (95% CI, 0.77-0.98)<br>• Q5, HR: 0.79 (95% CI, 0.70-0.90); *P* for trend <.001	NA
Haveman-Nies et al,[32] 2002	1251	Adjusted Mediterranean Diet Score; low (MDS<4) vs high	Female participants	• HR: 1.26 (95% CI, 0.88-1.81)	Race/ethnicity
			Male participants	• HR: 1.25 (95% CI, 0.93-1.68)	NA
Hodge et al,[33] 2011	40 470	mMDS	Female participants	• HR: 0.94 (95% CI, 0.92-0.97)	Race/ethnicity; smoking status (men)
			Male participants	• HR: 0.96 (95% CI, 0.93-0.99)	NA
Hodge et al,[34] 2018	39 532	mMDS	NA	• 0-3, 1 [Reference]<br>• 4-6, HR: 0.91 (95% CI, 0.87-0.96)<br>• 7-9, HR: 0.86 (95% CI, 0.80-0.93)<br>• Linear, HR: 0.96 (95% CI, 0.95-0.98); *P* for trend <.001	Physical activity; anthropometry; smoking status
Hu et al,[35] 2020	12 413	AHEI 2010	NA	• Q1, 1 [Reference]<br>• Q2, HR: 0.95 (95% CI, 0.88-1.03)	Anthropometry

© 2021 English LK et al. *JAMA Network Open.*

				• Q3, HR: 0.92 (95% CI, 0.85-1.00)<br>• Q4, HR: 0.84 (95% CI, 0.78-0.92)<br>• Q5, HR: 0.80 (95% CI, 0.73-0.87); *P* for trend <.001	
		aMED	NA	• Q1, 1 [Reference]<br>• Q2, HR: 0.93 (95% CI, 0.86-1.01)<br>• Q3, HR: 0.93 (95% CI, 0.86-1.01)<br>• Q4, HR: 0.84 (95% CI, 0.77-0.91)<br>• Q5, HR: 0.76 (95% CI, 0.70-0.83); P for trend <.001	NA
		DASH	NA	• Q1, 1 [Reference]<br>• Q2, HR: 0.94 (95% CI, 0.87-1.02)<br>• Q3, HR: 0.96 (95% CI, 0.88-1.03)<br>• Q4, HR: 0.93 (95% CI, 0.85-1.02)<br>• Q5, HR: 0.88 (95% CI, 0.80-0.96); *P* for trend <.01	NA
		HEI 2015	NA	• Q1, 1 [Reference]<br>• Q2, HR: 0.90 (95% CI, 0.83-0.97)<br>• Q3, HR: 0.85 (95% CI, 0.78-0.92)<br>• Q4, HR: 0.87 (95% CI, 0.80-0.95)<br>• Q5, HR: 0.82 (95% CI, 0.75-0.89); *P* for trend <.001	NA

© 2021 English LK et al. *JAMA Network Open.*

Hulsegge et al,[36] 2016	5623	mMDS	NA	• Increased mMDS, Δ from <5 at baseline to ≥5 at follow-up, HR: 1.09 (95% CI, 0.73-1.63)<br>• Decreased mMDS, Δ ≥5 at baseline and <5 at follow-up, HR: 1.19 (95% CI, 0.72-1.96)	Race/ethnicity
Kaluza et al,[37] 2009	40 837	NRFS	NA	• Low, 1 [Reference]<br>• Medium, HR: 1.04 (95% CI, 0.96-1.14)<br>• High, HR: 1.21 (95% CI, 1.09-1.34); *P* for trend = .001	NA
		RFS	NA	• Low, 1 [Reference]<br>• Medium, HR: 0.92 (95% CI, 0.85-1.00)<br>• High, HR: 0.81 (95% CI, 0.71-0.91); *P* for trend = .001	NA
Kaluza et al,[38] 2019	68 273	AIDI	Female participants	• Q1, 1 [Reference]<br>• Q2, HR: 0.97 (95% CI, 0.91-1.03)<br>• Q3, HR: 0.93 (95% CI, 0.87-0.99)<br>• Q4, HR: 0.85 (95% CI, 0.80-0.91); *P* for trend <.001<br>• Per 1-point increase, HR: 0.96 (95% CI, 0.95-0.98)	Race/ethnicity

© 2021 English LK et al. *JAMA Network Open.*

				• Per 1-point increase and 20th survival, PD: 0.2 (95% CI, 0.1-0.3)	
			Male participants	• Q1, 1 [Reference]<br>• Q2, HR: 0.89 (95% CI, 0.85-0.94)<br>• Q3, HR: 0.86 (95% CI, 0.81-0.92)<br>• Q4, HR: 0.80 (95% CI, 0.75-0.86); *P* for trend <.001<br>• Per 1-point increase, HR: 0.95 (95% CI, 0.94-0.96)<br>• Per 1-point increase and survival, 20th PD: 0.2 (95% CI, 0.1-0.3)	NA
			Female and male participants	• Q1, 1 [Reference]<br>• Q2, HR: 0.93 (95% CI, 0.89-0.97)<br>• Q3, HR: 0.89 (95% CI, 0.85-0.93)<br>• Q4, HR: 0.82 (95% CI, 0.78-0.86); *P* for trend <.001<br>• Per 1-point increase, HR: 0.96 (95% CI, 0.95-0.97)<br>• Per 1-point increase and 20th survival, PD: 0.2 (95% CI, 0.2-0.3)	NA
Kant et al,[39] 2000	42 254	RFS	NA	• Q1, 1 [Reference]<br>• Q2, HR: 0.82 (95% CI, 0.73-0.92)<br>• Q3, HR: 0.71 (95% CI, 0.62-0.81)	Physical activity

© 2021 English LK et al. *JAMA Network Open.*

				• Q4, HR: 0.69 (95% CI, 0.61-0.78)<br>• χ^2-trend 35.64; *P* for trend <.001	
Kant et al,[40] 2004	10 084	RFBS [9-11, 12-14, ≥15 vs 0-8]	Female participants	• 0-8, 1 [Reference]<br>• 9-11, RR: 0.95 (95% CI, 0.76-1.18)<br>• 12-14, RR: 0.80 (95% CI, 0.64-1.02)<br>• 15, RR: 0.80 (95% CI, 0.61-1.04); *P* for trend = .04	Physical activity
			Male participants	• 0-8, 1 [Reference]<br>• 9-11, RR: 0.88 (95% CI, 0.72-1.08)<br>• 12-14, RR: 0.84 (95% CI, 0.68-1.03)<br>• 15, RR: 0.72 (95% CI, 0.56-0.92); *P* for trend = .001	NA
		Fruit, vegetable, whole grain[e]	Female participants	• Q1, 1 [Reference]<br>• Q2, RR: 0.89 (95% CI, 0.72-1.08)<br>• Q3, RR: 0.81 (95% CI, 0.64-1.02)<br>• Q4, RR: 0.87 (95% CI, 0.67-1.11); *P* for trend = .09	NA
			Male participants	• Q1, 1 [Reference]<br>• Q2, RR: 0.92 (95% CI, 0.74-1.13)	NA

© 2021 English LK et al. *JAMA Network Open.*

				• Q3, RR: 0.84 (95% CI, 0.68-1.06)<br>• Q4, RR: 0.74 (95% CI, 0.57-0.95); *P* for trend = .002	
		Ethnic[e]	Female participants	• NS, data NR	NA
			Male participants	• NS, data NR	NA
		Low-fat[e]	Female participants	• NS, data NR	NA
			Male participants	• NS, data NR	NA
		Cluster 1[e]	Female participants	• Cluster 1, 1 [Reference]	NA
		Cluster 2[e]		• Cluster 2, RR: 0.93 (95% CI, 0.75-1.16)	
		Cluster 3[e]		• Cluster 3, RR: 0.93 (95% CI, 0.74-1.17)	
		Cluster 4[e]		• Cluster 4, RR: 0.88 (95% CI, 0.72-1.09)	
			Male participants	• Cluster 1, 1 [Reference]<br>• Cluster 2, RR: 0.94 (95% CI, 0.76-1.16)<br>• Cluster 3, RR: 0.87 (95% CI, 0.71-1.07)<br>• Cluster 4, RR: 0.82 (95% CI, 0.66-1.01)	NA

© 2021 English LK et al. *JAMA Network Open.*

Kant et al,[41] 2009	350 886	DBS	Female participants	• Q1, 1 [Reference]<br>• Q2, RR: 0.90 (95% CI, 0.85-0.95)<br>• Q3, RR: 0.87 (95% CI, 0.82-0.93)<br>• Q4, RR: 0.80 (95% CI, 0.75-0.86)<br>• Q5, RR: 0.75 (95% CI, 0.70-0.80); *P* for trend <.001	NA
			Male participants	• Q1, 1 [Reference]<br>• Q2, RR: 0.90 (95% CI, 0.86-0.94)<br>• Q3, RR: 0.88 (95% CI, 0.85-0.92)<br>• Q4, RR: 0.83 (95% CI, 0.79-0.87)<br>• Q5, RR: 0.79 (95% CI, 0.75-0.83); *P* for trend <.001	NA
Kappeler et al,[42] 2013	17 611	HEI	Female participants	• HEI: <51, Poor, HR: 1.00<br>• HEI: 51-80, Needs Improvement, HR: 1.00 (95% CI, 0.73-1.36)<br>• HEI: >80, Good, HR: 0.88 (95% CI, 0.65-1.20); *P* for trend = .29	NA
			Male participants	• HEI: <51, Poor, 1 [Reference]<br>• HEI: 51-80, Needs Improvement, HR: 0.85 (95% CI, 0.70-1.04)<br>• HEI: >80, Good, HR: 0.70 (95% CI, 0.52-0.96); *P* for trend = .02	NA

© 2021 English LK et al. *JAMA Network Open.*

			Female and male participants	• HEI: <51, Poor, 1 [Reference]<br>• HEI: 51-80, Needs Improvement, HR: 0.90 (95% CI, 0.75-1.08)<br>• HEI: >80, Good, HR: 0.77 (95% CI, 0.63-0.94); *P* for trend = .01	NA
Kim et al,[43] 2013	12 538	Healthy diet score	NA	• <2 components, 1 [Reference]<br>• ≥2 components, HR: 0.81 (95% CI, 0.57-1.14)	NA
Kim et al,[44] 2018	11 879	hPDI	Female participants	• ≥Median, HR: 0.94 (95% CI, 0.88-0.99)<br>• <Median, HR: 1.09 (95% CI, 0.98-1.19)	NA
			Male participants	• <Median, HR: 1.01 (95% CI, 0.92-1.10)<br>• ≥Median, HR: 0.95 (95% CI, 0.89-1.01)	NA
			Female and male participants	• ≥Median, HR: 0.95 (95% CI, 0.91-0.98)<br>• <Median, HR: 1.04 (95% CI, 0.97-1.12)	NA
		uPDI	Female participants	• HR: 1.01 (95% CI, 0.98-0.05)	NA
			Male participants	• HR: 1.01 (95% CI, 0.98-1.06)	NA

© 2021 English LK et al. *JAMA Network Open.*

			Female and male participants	• HR: 1.00 (95% CI, 0.98-1.04)	NA
		PDI	Female participants	• HR: 0.98 (95% CI, 0.95-1.00)	NA
			Male participants	• HR: 1.04 (95% CI, 0.99-1.07)	NA
			Female and male participants	• HR: 1.01 (95% CI, 0.98-1.03)	NA
Kim et al,[45] 2019	12 168	hPDI	NA	• Q1, 1 [Reference]<br>• Q2, HR: 0.99 (95% CI, 0.91-1.07)<br>• Q3, HR: 0.99 (95% CI, 0.91-1.08)<br>• Q4, HR: 0.93 (95% CI, 0.85-1.02)<br>• Q5, HR: 0.91 (95% CI, 0.83-1.00); *P* for trend = .03	NA
		uPDI	NA	• Q1, 1 [Reference]<br>• Q2, HR: 1.04 (95% CI, 0.96-1.12)<br>• Q3, HR: 0.97 (95% CI, 0.89-1.05)<br>• Q4, HR: 1.01 (95% CI, 0.93-1.10)<br>• Q5, HR: 1.02 (95% CI, 0.94-1.11); *P* for trend = .67	NA
		Provegetarian food pattern	NA	• Q1, 1 [Reference]<br>• Q2, HR: 0.92 (95% CI, 0.85-0.99)<br>• Q3, HR: 0.89 (95% CI, 0.82-0.97)	NA

© 2021 English LK et al. *JAMA Network Open.*

				• Q4, HR: 0.84 (95% CI, 0.77-0.91)<br>• Q5, HR: 0.82 (95% CI, 0.76-0.89); *P* for trend <.001	
		PDI	NA	• Q1, 1 [Reference]<br>• Q2, HR: 0.89 (95% CI, 0.83-0.97)<br>• Q3, HR: 0.82 (95% CI, 0.76-0.89)<br>• Q4, HR: 0.82 (95% CI, 0.75-0.89)<br>• Q5, HR: 0.76 (95% CI, 0.69-0.83); *P* for trend <.001	NA
Knoops et al,[46] 2004	2339	mMDS	NA	• HR: 0.77 (95% CI, 0.68-0.88)	Race/ethnicity
Knoops et al,[47] 2006	3117	mMDS	NA	• HR: 0.82 (95% CI, 0.75-0.91); *P* < .05<br>• Removing alcohol, HR: 0.78 (95% CI, 0.71-0.87); *P* < .05	Race/ethnicity
		MAI	NA	• HR: 0.83 (95% CI, 0.75-0.92); *P* < .05<br>• Removing alcohol, HR: 0.87 (95% CI, 0.79-0.97); *P* < .05	NA
Kurotani et al,[48] 2016	79 594	Japanese Food Guide score	NA	• Q1, 1 [Reference]<br>• Q2, HR: 0.92 (95% CI, 0.87-0.97)<br>• Q3, HR: 0.88 (95% CI, 0.83-0.93)	Race/ethnicity

© 2021 English LK et al. *JAMA Network Open.*

				• Q4, HR: 0.85 (95% CI, 0.7-0.91); *P* for trend <.001<br>• Per 10-point increment, HR: 0.93 (95% CI, 0.91-0.95)	
		Modified Japanese Food Guide score	NA	• Q1, 1 [Reference]<br>• Q2, HR: 0.93 (95% CI, 0.89-0.98)<br>• Q3, HR: 0.84 (95% CI, 0.79-0.89)<br>• Q4, HR: 0.82 (95% CI, 0.77-0.88); *P* for trend <.001<br>• Continuous by 10-point increment, HR: 0.93 (95% CI, 0.91-0.95)	NA
Kurotani et al,[33] 2019	61 267	Japanese Food Guide, according to Japanese ADI tertiles	<Median	• T1 ADI, 1 [Reference]<br>• T2 ADI, HR: 1.17 (95% CI, 1.08-1.27)<br>• T3 ADI, HR: 1.19 (95% CI, 1.08-1.32)<br>• Across ADI tertiles, *P* for trend = .03	Alcohol intake
			≥Median	• T1 ADI, HR: 1.09 (95% CI, 0.99-1.19)<br>• T2 ADI, HR: 1.01 (95% CI, 0.93-1.10)<br>• T3 ADI, HR: 1.05 (95% CI, 0.96-1.16)	NA

© 2021 English LK et al. *JAMA Network Open.*

				• Across ADI tertiles, *P* for trend = .92	
Lagiou et al,[50] 2006	42 237	MDS	NA	• Per 2-unit increase, HR: 0.93 (95% CI, 0.83-1.03); *P* for trend = .18<br>• Low (0-3), 1 [Reference]<br>• Middle (4-5), HR: 0.93 (95% CI, 0.78-1.13)<br>• High (6-9), HR: 0.85 (95% CI, 0.67-1.08)	Race/ethnicity; alcohol intake NR
Lasheras et al,[51] 2000	161	mMDS	NA	• <80 y, n = 74, HR: 0.69 (95% CI, 0.43-0.93); *P* = .03<br>• ≥80 y, n = 87, HR: 1.24 (95% CI, 0.60-2.53); *P* = .55	Race/ethnicity; SES: institutionalize d
Lassale et al,[52] 2016	451 256	DASH score	NA	• Q1, 1 [Reference]<br>• Q2, HR: 0.90 (95% CI, 0.87-0.94)<br>• Q3, HR: 0.85 (95% CI, 0.81-0.89)<br>• Q4, HR: 0.82 (95% CI, 0.78-0.86)<br>• Q5, HR: 0.92 (95% CI, 0.90-0.93); *P* for trend <.001	NA
		MDS	NA	• Q1, 1 [Reference]<br>• Q2, HR: 0.90 (95% CI, 0.86-0.94)<br>• Q3, HR: 0.84 (95% CI, 0.81-0.88)<br>• Q4, HR: 0.79 (95% CI, 0.76-0.83)	NA

© 2021 English LK et al. *JAMA Network Open.*

				• Q5, HR: 0.91 (95% CI, 0.90-0.93); *P* for trend <.001	
		Mediterranean-style dietary pattern score	NA	• Q1, 1 [Reference]<br>• Q2, HR: 0.92 (95% CI, 0.89-0.96)<br>• Q3, HR: 0.88 (95% CI, 0.84-0.92)<br>• Q4, HR: 0.80 (95% CI, 0.76-0.84)<br>• Q5, HR: 0.92 (95% CI, 0.90-0.93); *P* for trend <.001	NA
		Relative Mediterranean diet score	NA	• Q1, 1 [Reference]<br>• Q2, HR: 0.87 (95% CI, 0.83-0.91)<br>• Q3, HR: 0.81 (95% CI, 0.77-0.84)<br>• Q4, HR: 0.77 (95% CI, 0.73-0.81)<br>• Q5, HR: 0.89 (95% CI, 0.88-0.91); *P* for trend <.001	NA
		DQI-I	NA	• Q1, 1 [Reference]<br>• Q2, HR: 0.89 (95% CI, 0.85-0.93)<br>• Q3, HR: 0.81 (95% CI, 0.77-0.85)<br>• Q4, HR: 0.75 (95% CI, 0.72-0.79)<br>• Q5, HR: 0.90 (95% CI, 0.88-0.91); *P* for trend <.001	NA
		HEI 2010	NA	• Q1, 1 [Reference]<br>• Q2, HR: 0.89 (95% CI, 0.85-0.93)	NA

© 2021 English LK et al. *JAMA Network Open.*

				• Q3, HR: 0.84 (95% CI, 0.80-0.88)<br>• Q4, HR: 0.82 (95% CI, 0.78-0.86)<br>• Q5, HR: 0.91 (95% CI, 0.90-0.93); *P* for trend <.001	
		HNFI	NA	• Q1, 1 [Reference]<br>• Q2, HR: 0.94 (95% CI, 0.90-0.98)<br>• Q3, HR: 0.87 (95% CI, 0.83-0.91)<br>• Q4, HR: 0.83 (95% CI, 0.79-0.87)<br>• Q5, HR: 0.93 (95% CI, 0.92-0.95); *P* for trend <.001	NA
		HLI-Diet	NA	• Q1, 1 [Reference]<br>• Q2, HR: 0.91 (95% CI, 0.88-0.96)<br>• Q3, HR: 0.86 (95% CI, 0.83-0.90)<br>• Q4, HR: 0.83 (95% CI, 0.79-0.87)<br>• Q5, HR: 0.93 (95% CI, 0.92-0.95); *P* for trend <.001	NA
Lim et al,[53] 2018	134 541	DQI-K	NA	• Higher diet quality, 0-4, 1 [Reference]<br>• Poorer diet quality, 5-9, HR: 1.23 (95% CI, 1.06-1.43)<br>• Per-unit increase, HR: 1.06 (95% CI, 1.02-1.11)	Physical activity

© 2021 English LK et al. *JAMA Network Open.*

Limongi et al,[54] 2017	2665	Mediterranean score	NA	• At ~ 4 y follow-up<br>• T1, 1 [Reference]<br>• T2, data NR<br>• T3, HR: 0.62 (95% CI, 0.42-0.92); *P* = .03	Race/ethnicity; physical activity
				• At ~ 8 y follow-up:<br>• T1, 1 [Reference]<br>• T2, HR: 0.72 (95% CI, 0.54-0.97)<br>• T3, HR: 0.66 (95% CI, 0.49-0.90); *P* < .01	
Liu et al,[55] 2019	1990	DST	NA	• Low, 1 [Reference]<br>• Moderate, HR: 0.93 (95% CI, 0.81-1.07)<br>• High, HR: 0.76 (95% CI, 0.59-0.97); *P* for trend = .04	SES; alcohol intake; physical activity
Loprinzi et al,[56] 2018	1369	AHEI 2005	NA	• Per-unit increase, HR: 0.97 (95% CI, 0.96-0.99); *P* = .004<br>• Meeting dietary guidelines vs not, HR: 0.60 (95% CI, 0.38-0.97); *P* = .03	NA
Mai et al,[57] 2005	42 254	RFS	NA	• Q1, 1 [Reference]<br>• Q2, HR: 0.87 (95% CI, 0.80-0.95)	NA

© 2021 English LK et al. *JAMA Network Open.*

				• Q3, HR: 0.78 (95% CI, 0.71-0.86)<br>• Q4, HR: 0.80 (95% CI, 0.73-0.88); *P* for trend <.001	
Martínez-Gómez et al,[58] 2013	3465	Healthy diet score	NA	• <Median, 1 [Reference]<br>• ≥Median, HR: 0.79 (95% CI, 0.79-0.89)	NA
Martínez-González et al,[59] 2012	15 535	MDS	Female and male participants	• Low, 1 [Reference]<br>• Moderate, HR: 0.58 (95% CI, 0.34-0.99)<br>• High, HR: 0.38 (95% CI, 0.21-0.70)<br>• Per-unit increase, HR: 0.72 (95% CI, 0.58-0.91); *P* for trend = .006	Race/ethnicity
			Female participants	• HR: 0.83 (95% CI, 0.53-1.29); P = .41	
Martínez-González et al,[60] 2014	7216	Provegetarian food pattern	NA	• Very low, <30, 1 [Reference]<br>• Low, 30-34, HR: 0.71 (95% CI, 0.50-1.02)<br>• Moderate, 35-39, HR: 0.68 (95% CI, 0.48-0.96)<br>• High, 40+, HR: 0.59 (95% CI, 0.40-0.88); *P* for trend = .03<br>• Yearly updated, low 30-34, RR: 0.76 (95% CI, 0.53-1.10)	Race/ethnicity; anthropometry

© 2021 English LK et al. *JAMA Network Open.*

				• Yearly updated, moderate 35-39, RR: 0.79 (95% CI, 0.55-1.13)<br>• Yearly updated, high 40+, RR: 0.59 (95% CI, 0.39-0.89); *P* for trend = .03	
				• Q1, <33, 1 [Reference]<br>• Q2, 33-35, HR: 0.98 (95% CI, 0.72-1.32)<br>• Q3, 36-37, HR: 0.81 (95% CI, 0.57-1.14)<br>• Q4, 38-40, HR: 0.70 (95% CI, 0.49-0.99)<br>• Q5: >40, HR: 0.66 (95% CI, 0.46-0.96); *P* for trend = .006	
McCullough et al,[61] 2011	111 966	Healthy diet score	Female participants	• <3 score, 1 [Reference]<br>• 3-5 score, RR: 0.91 (95% CI, 0.85-0.98)<br>• +6 score, RR: 0.85 (95% CI, 0.79-0.90); *P* for trend <.001	NA
			Male participants	• <3 score, 1 [Reference]<br>• 3-5 score, RR: 0.90 (95% CI, 0.86-0.95)<br>• +6 score, RR: 0.89 (95% CI, 0.84-0.93); *P* for trend <.001	NA

© 2021 English LK et al. *JAMA Network Open.*

McNaughton et al,[62] 2012	972	MDS	NA	• Q1, 1 [Reference]<br>• Q2, HR: 1.04 (95% CI, 0.85-1.27)<br>• Q3, HR: 0.77 (95% CI, 0.61-0.97)<br>• Q4, HR: 0.78 (95% CI, 0.62-0.98); *P* for trend = .006	Race/ethnicity
		RFS	NA	• RFS score<br>• Q1, 1 [Reference]<br>• Q2, HR: 0.90 (95% CI, 0.74-1.10)<br>• Q3, HR: 0.76 (95% CI, 0.61-0.96)<br>• Q4, HR: 0.67 (95% CI, 0.52-0.86); *P* for trend = .001	NA
				• RFS median<br>• Q1, 1 [Reference]<br>• Q2, HR: 0.78 (95% CI, 0.64-0.94)<br>• Q3, HR: 0.85 (95% CI, 0.68-1.07)<br>• Q4, HR: 0.63 (95% CI, 0.48-0.83); *P* for trend = .003	
		Healthy diet score	NA	• Q1, 1 [Reference]<br>• Q2, HR: 1.10 (95% CI, 0.90-1.35)<br>• Q3, HR: 0.98 (95% CI, 0.79-1.22)<br>• Q4, HR: 0.99 (95% CI, 0.79-1.24); *P* for trend = .80	NA

© 2021 English LK et al. *JAMA Network Open.*

Menotti et al,[124] 2012	1139	MAI	NA	• 20 y follow-up, HR: 0.74 (95% CI, 0.55-0.99)<br>• 40 y follow-up, HR: 0.79 (95% CI, 0.64-0.97)	Race/ethnicity; SES
Menotti et al,[64] 2017	12 696	MAI	NA	• Death rates over 50 y follow-up, *r* = −0.62; *P* < .05<br>• Death rates over 50 y Follow-up, using 25 y rates, *r* = 0.98; *P* value NR<br>• Death rates over 50 y follow-up, using 45 y rates, *r* = .99; *P* value NR	Race/ethnicity; alcohol intake; physical activity; anthropometry ; smoking status
Michels and Wolk,[65] 2002	59 038	NRFS	NA	• Q1, 1 [Reference]<br>• Q2, HR: 1.00 (95% CI, 0.90-1.11)<br>• Q3, HR: 0.98 (95% CI, 0.88-1.09)<br>• Q4, HR: 0.98 (95% CI, 0.87-1.11)<br>• Q5, HR: 1.07 (95% CI, 0.88-1.31); *P* for trend = .92	Race/ethnicity; physical activity; smoking status
		RFS	NA	• Q1, 1 [Reference]<br>• Q2, HR: 0.79 (95% CI, 0.70-0.88)<br>• Q3, HR: 0.71 (95% CI, 0.63-0.80)<br>• Q4, HR: 0.64 (95% CI, 0.57-0.72)<br>• Q5, HR: 0.58 (95% CI, 0.50-0.68); *P* for trend <.001	NA

© 2021 English LK et al. *JAMA Network Open.*

Mitrou et al,[66] 2007	380 296	MDS	Female participants	• HR: 0.84 (95% CI, 0.79-0.89)	NA
			Male participants	• HR: 0.79 (95% CI, 0.76-0.82)	NA
		Traditional Mediterranean diet score	Female participants	• 0-3, 1 [Reference]<br>• 4-5, HR: 0.89 (95% CI, 0.85-0.93)<br>• 6-9, HR: 0.80 (95% CI, 0.75-0.85); *P* for trend <.001	NA
			Male participants	• 0-3, 1 [Reference]<br>• 4-5, HR: 0.91 (95% CI, 0.88-0.94)<br>• 6-9, HR: 0.79 (95% CI, 0.76-0.83); *P* for trend <.001	NA
Mokhtari et al,[67] 2019	48 633	DASH score	Female participants	• DS 9-20, 1 [Reference]<br>• DS 21-25, HR: 0.92 (95% CI, 0.84-1.02)<br>• DS 26-30, HR: 0.86 (95% CI, 0.77- 0.97)<br>• DS 31-39, HR: 0.90 (95% CI, 0.75-0.99); *P* for trend = .03	Alcohol intake
			Male participants	• DS 9-20, 1 [Reference]<br>• DS 21-25, HR: 0.94 (95% CI, 0.86-1.02)<br>• DS 26-30, HR: 0.87 (95% CI, 0.79-0.96)	NA

© 2021 English LK et al. *JAMA Network Open.*

				• DS 31-39, HR: 0.82 (95% CI, 0.68-0.98); *P* for trend = .003	
			Female and male participants	• DS 9-20, 1 [Reference]<br>• DS 21-25, HR: 0.94 (95% CI, 0.88-1.00)<br>• DS 26-30, HR: 0.87 (95% CI, 0.81-0.94)<br>• DS 31-39, HR: 0.86 (95% CI, 0.75-0.98); *P* for trend <.001	NA
Muller et al,[68] 2016	264 906	WCRF/AICR score (diet only)	NA	• Unhealthy, 1 [Reference]<br>• Moderately unhealthy, HR: 0.88 (95% CI, 0.83-0.93)<br>• Moderately healthy, HR: 0.81 (95% CI, 0.76-0.87)<br>• Healthy, HR: 0.87 (95% CI, 0.72-0.83); *P* value NR	Race/ethnicity; SES
Mursu et al,[69] 2013	29 634	AHEI 2010	NA	• Q1, 1 [Reference]<br>• Q2, HR: 0.98 (95% CI, 0.92-1.03)<br>• Q3, HR: 0.90 (95% CI, 0.85-0.95)<br>• Q4, HR: 0.82 (95% CI, 0.77-0.87); *P* for trend <.001<br>• Per-SD increase, HR: 0.92 (95% CI, 0.91-0.94); *P* value NR	NA

© 2021 English LK et al. *JAMA Network Open.*

		A priori diet quality score	NA	• Q1, 1 [Reference]<br>• Q2, HR: 0.93 (95% CI, 0.88-0.98)<br>• Q3, HR: 0.87 (95% CI, 0.82-0.92)<br>• Q4, HR: 0.80 (95% CI, 0.76-0.85); *P* for trend <.001<br>• Per-SD increase, HR: 0.92 (95% CI, 0.90-0.94); *P* value NR	NA
Nakamura et al,[70] 2009	9086	Reduced-salt Japanese diet score	NA	• Score 0-2, 1 [Reference]<br>• Score 3, HR: 0.92 (95% CI, 0.83-1.04)<br>• Score 4-7, HR: 0.78 (95% CI, 0.70-0.88); *P* for trend <.001	SES; alcohol intake; physical activity
Neelakantan et al,[71] 2018	57 078	aMED	NA	• Q1, 1 [Reference]<br>• Q2, HR: 0.96 (95% CI, 0.92-1.01)<br>• Q3, HR: 0.93 (95% CI, 0.89-0.98)<br>• Q4, HR: 0.88 (95% CI, 0.83-0.92)<br>• Q5, HR: 0.80 (95% CI, 0.76-0.85); *P* for trend <.001	NA
		DASH score	NA	• Q1, 1 [Reference]<br>• Q2, HR: 0.91 (95% CI, 0.86-0.96)<br>• Q3, HR: 0.87 (95% CI, 0.82-0.92)<br>• Q4, HR: 0.85 (95% CI, 0.80-0.89)	NA

© 2021 English LK et al. *JAMA Network Open.*

				• Q5, HR: 0.80 (95% CI, 0.75-0.84); *P* for trend <.001	
		aHEI	NA	• Q1, 1 [Reference]<br>• Q2, HR: 0.93 (95% CI, 0.88-0.98)<br>• Q3, HR: 0.89 (95% CI, 0.85-0.93)<br>• Q4, HR: 0.86 (95% CI, 0.82-0.90)<br>• Q5, HR: 0.82 (95% CI, 0.78-0.86); *P* for trend <.001	NA
Nilsson et al,[72] 2012	77 319	Sami diet score	Female participants	• HR: 1.03 (95% CI, 0.99-1.07); P = .13	Race/ethnicity
			Male participants	• HR: 1.04 (95% CI, 1.01-1.07); P = .02	NA
Oba et al,[73] 2009	29 079	Japanese Food Guide Spinning Top score	Female participants	• Q1, 1 [Reference]<br>• Q2, HR: 0.87 (95% CI, 0.73-1.05)<br>• Q3, HR: 0.86 (95% CI, 0.72-1.04)<br>• Q4, HR: 0.78 (95% CI, 0.65-0.94); *P* for trend = .01	NA
			Male participants	• Q1, 1 [Reference]<br>• Q2, HR: 0.90 (95% CI, 0.76-1.06)<br>• Q3, HR: 0.87 (95% CI, 0.73-1.02)<br>• Q4, HR: 1.01 (95% CI, 0.86-1.19); *P* for trend = .91	NA

© 2021 English LK et al. *JAMA Network Open.*

Okada et al,[74] 2018	58 767	Japan food score	Female participants	• Score 0-2, 1 [Reference]<br>• Score 3, HR: 0.92 (95% CI, 0.82-1.03)<br>• Score 4, HR: 0.99 (95% CI, 0.89-1.09)<br>• Score 5, HR: 0.85 (95% CI, 0.77-0.94)<br>• Score 6-7, HR: 0.82 (95% CI, 0.75-0.90); *P* for trend <.001	NA
			Male participants	• Score 0-2, 1 [Reference]<br>• Score 3, HR: 0.96 (95% CI, 0.88-1.04)<br>• Score 4, HR: 0.92 (95% CI, 0.84-1.00)<br>• Score 5, HR: 0.95 (95% CI, 0.88-1.03)<br>• Score 6-7, HR: 0.93 (95% CI, 0.86-1.01); *P* for trend = .07	NA
Olsen et al,[75] 2011	50 290	HNFI	Female participants	• per 1-point, RR: 0.96 (95% CI, 0.92-1.00); *P* for trend = .03<br>• 0, 1 [Reference]<br>• 1, RR: 0.96 (95% CI, 0.75-1.23)<br>• 2, RR: 0.87 (95% CI, 0.68-1.10)<br>• 3, RR: 0.81 (95% CI, 0.63-1.04)	Race/ethnicity

© 2021 English LK et al. *JAMA Network Open.*

				• 4, RR: 0.81 (95% CI, 0.62-1.05)<br>• 5, RR: 0.84 (95% CI, 0.63-1.12)<br>• 6, RR: 0.75 (95% CI, 0.49-1.15)	
			Male participants	• per 1-point, RR: 0.96 (95% CI, 0.92- 0.99); *P* for trend = .005<br>• 0, 1 [Reference]<br>• 1, RR: 0.76 (95% CI, 0.61-0.94)<br>• 2, RR: 0.69 (95% CI, 0.55-0.85)<br>• 3, RR: 0.68 (95% CI, 0.55-0.85)<br>• 4, RR: 0.64 (95% CI, 0.51-0.81)<br>• 5, RR: 0.67 (95% CI, 0.52-0.85)<br>• 6, RR: 0.64 (95% CI, 0.46-0.89)	NA
Osler et al,[76] 2001	5872	Healthy food index[e]	Female participants	• per SD, HR: 0.96 (95% CI, 0.85-1.09)<br>• 0 point, 1 [Reference]<br>• 1 point, HR: 0.80 (95% CI, 0.53-1.20)<br>• 2 points, HR: 0.71 (95% CI, 0.46-1.07)<br>• 3 + 4 points, HR: 0.82 (95% CI, 0.54-1.25)	Age; race/ethnicity
			Male participants	• per SD, HR: 0.86 (95% CI, 0.86-1.05)	NA

© 2021 English LK et al. *JAMA Network Open.*

				• 0 point, 1 [Reference]<br>• 1 point, HR: 0.73 (95% CI, 0.56-0.98)<br>• 2 points, HR: 0.78 (95% CI, 0.59-1.02)<br>• 3 + 4 points, HR: 0.82 (95% CI, 0.58-1.14)	
		Prudent[e]	Female participants	• per SD, HR: 0.74 (95% CI, 0.64-0.85)<br>• Q1, 1 [Reference]<br>• Q2, HR: 0.69 (95% CI, 0.50-0.96)<br>• Q3, HR: 0.57 (95% CI, 0.40-0.82)<br>• Q4, HR: 0.46 (95% CI, 0.30-0.72)	NA
			Male participants	• per SD, HR: 0.84 (95% CI, 0.75-0.93)<br>• Q1, 1 [Reference]<br>• Q2, HR: 0.87 (95% CI, 0.68-1.11)<br>• Q3, HR: 0.71 (95% CI, 0.53-0.96)<br>• Q4, HR: 0.70 (95% CI, 0.49-1.00)	NA
		Western[e]	Female participants	• per SD, HR: 0.91 (95% CI, 0.80-1.03)<br>• Q1, 1 [Reference]<br>• Q2, HR: 0.93 (95% CI, 0.67-1.29)	NA

© 2021 English LK et al. *JAMA Network Open.*

				• Q3, HR: 0.65 (95% CI, 0.44-0.94)<br>• Q4, HR: 0.87 (95% CI, 0.59-1.29)	
			Male participants	• per SD, HR: 1.01 (95% CI, 0.90-1.12)<br>• Q1, 1 [Reference]<br>• Q2, HR: 0.81 (95% CI, 0.60-1.09)<br>• Q3, HR: 0.97 (95% CI, 0.73-1.29)<br>• Q4, HR: 0.92 (95% CI, 0.69-1.23)	NA
Panizza et al,[77] 2018	156 804	HEI 2015	Female participants	• Q1, 1 [Reference]<br>• Q2, HR: 0.92 (95% CI, 0.89-0.96)<br>• Q3, HR: 0.87 (95% CI, 0.84-0.91)<br>• Q4, HR: 0.82 (95% CI, 0.79-0.86)<br>• Q5, HR: 0.79 (95% CI, 0.76-0.82); $P < .05$	NA
			Male participants	• Q1, 1 [Reference]<br>• Q2, HR: 0.93 (95% CI, 0.90-0.97)<br>• Q3, HR: 0.89 (95% CI, 0.85-0.92)<br>• Q4, HR: 0.85 (95% CI, 0.81-0.88)<br>• Q5, HR: 0.79 (95% CI, 0.76-0.82); $P < .05$	NA
Park, Steck, Fung, et al,[78] 2016	1739	MedDietScore	Metabolically healthy obesity	• T1, 1 [Reference]	NA

© 2021 English LK et al. *JAMA Network Open.*

				• T2, HR: 0.35 (95% CI, 0.19-0.64)<br>• T3, HR: 0.44 (95% CI, 0.26-0.75); *P* for trend <.001<br>• per 5-pt increase, HR: 0.59 (95% CI, 0.37-0.94)	
			Metabolically unhealthy obesity	• T1, 1 [Reference]<br>• T2, HR: 0.74 (95% CI, 0.58-0.95)<br>• T3, HR: 0.92 (95% CI, 0.48-1.76); *P* for trend = .66<br>• per 5-pt increase, HR: 0.96 (95% CI, 0.78-1.17)	NA
Park, Fung, Steck, et al,[79] 2016	2103	HEI	Metabolically healthy normal weight phenotype	• T1, 1 [Reference]<br>• T2, HR: 0.64 (95% CI, 0.39-1.05)<br>• T3, HR: 0.68 (95% CI, 0.44-1.05); *P* for trend = .09<br>• per unit increase, HR: 0.83 (95% CI, 0.70-1.00)	NA
			Metabolically obese normal weight phenotype	• T1, 1 [Reference]<br>• T2, HR: 0.59 (95% CI, 0.44-0.79)<br>• T3, HR: 0.54 (95% CI, 0.39-0.75); *P* for trend <.001<br>• Per-unit increase, HR: 0.78 (95% CI, 0.68-0.90)	NA

© 2021 English LK et al. *JAMA Network Open.*

Prinelli et al,[80] 2015	974	MedDietScore		• Low, 1 [Reference]<br>• Medium, HR: 0.79 (95% CI, 0.43-1.12)<br>• High, HR: 0.62 (95% CI, 0.43-0.89); *P* for trend = .01<br>• Per-unit increase, HR: 0.95 (95% CI, 0.92-0.98)	Race/ethnicity
		MDS		• Tertiles, [Reference] NR, HR: 0.69 (95% CI, 0.46-1.03); *P* for trend = .07	NA
Reedy et al,[81] 2014	424 662	AHEI 2010	Female participants	• Q1, 1 [Reference]<br>• Q2, HR: 0.91 (95% CI, 0.88-0.94)<br>• Q3, HR: 0.85 (95% CI, 0.83-0.88)<br>• Q4, HR: 0.85 (95% CI, 0.82-0.88)<br>• Q5, HR: 0.76 (95% CI, 0.74-0.79); *P* for trend <.05	NA
			Male participants	• Q1, 1 [Reference]<br>• Q2, HR: 0.91 (95% CI, 0.89-0.93)<br>• Q3, HR: 0.88 (95% CI, 0.86-0.91)<br>• Q4, HR: 0.83 (95% CI, 0.81-0.86)<br>• Q5, HR: 0.76 (95% CI, 0.76-0.80); *P* for trend <.05	NA

© 2021 English LK et al. *JAMA Network Open.*

		aMED	Female participants	• Q1, 1 [Reference]<br>• Q2, HR: 0.94 (95% CI, 0.90-0.97)<br>• Q3, HR: 0.89 (95% CI, 0.86-0.92)<br>• Q4, HR: 0.83 (95% CI, 0.80-0.86)<br>• Q5, HR: 0.76 (95% CI, 0.73-0.79); *P* for trend <.05	NA
			Male participants	• Q1, 1 [Reference]<br>• Q2, HR: 0.92 (95% CI, 0.90-0.94)<br>• Q3, HR: 0.88 (95% CI, 0.85-0.90)<br>• Q4, HR: 0.83 (95% CI, 0.81-0.85)<br>• Q5, HR: 0.77 (95% CI, 0.75-0.79); *P* for trend <.05	NA
		DASH score	Female participants	• Q1, 1 [Reference]<br>• Q2, HR: 0.93 (95% CI, 0.90-0.96)<br>• Q3, HR: 0.87 (95% CI, 0.84-0.89)<br>• Q4, HR: 0.82 (95% CI, 0.79-0.85)<br>• Q5, HR: 0.78 (95% CI, 0.75-0.81); *P* for trend <.05	NA
			Male participants	• Q1, 1 [Reference]<br>• Q2, HR: 0.95 (95% CI, 0.92-0.97)<br>• Q3, HR: 0.90 (95% CI, 0.88-0.93)	NA

© 2021 English LK et al. *JAMA Network Open.*

				• Q4, HR: 0.87 (95% CI, 0.85-0.90)<br>• Q5, HR: 0.83 (95% CI, 0.80-0.85); *P* for trend <.05	
		HEI 2010	Female participants	• Q1, 1 [Reference]<br>• Q2, HR: 0.88 (95% CI, 0.85-0.91)<br>• Q3, HR: 0.88 (95% CI, 0.85-0.91)<br>• Q4, HR: 0.82 (95% CI, 0.79-0.85)<br>• Q5, HR: 0.77 (95% CI, 0.74-0.80); *P* for trend <.05	NA
			Male participants	• Q1, 1 [Reference]<br>• Q2, HR: 0.91 (95% CI, 0.88-0.93)<br>• Q3, HR: 0.86 (95% CI, 0.83-0.88)<br>• Q4, HR: 0.83 (95% CI, 0.81-0.85)<br>• Q5, HR: 0.78 (95% CI, 0.76-0.80); *P* for trend <.05	NA
Roswall et al,[82] 2015	44 961	HNFI	NA	• Per-unit increase and MRR, MRR: 0.94 (95% CI, 0.91-0.97); *P* < .001<br>• low 0-1, 1 [Reference]<br>• middle 2-3, MRR: 0.88 (95% CI, 0.79-0.99)<br>• high 4-6, MRR: 0.82 (95% CI, 0.71-0.93)	Race/ethnicity

© 2021 English LK et al. *JAMA Network Open.*

Seymour et al,[83] 2003	115 833	DQI	Female participants	• High, 1 [Reference]<br>• Medium-high, RR: 1.09 (95% CI, 0.87-1.38)<br>• Medium, RR: 1.15 (95% CI, 0.91-1.45)<br>• Medium-low, RR: 1.31 (95% CI, 1.04-1.65)<br>• Low, RR: 1.23 (95% CI, 0.84-1.81); *P* for trend = .02	Anthropometry
			Male participants	• High, 1 [Reference]<br>• Medium-high, RR: 1.06 (95% CI, 0.85-1.31)<br>• Medium, RR: 1.08 (95% CI, 0.88-1.33)<br>• Medium-low, RR: 1.17 (95% CI, 0.96-1.44)<br>• Low, RR: 1.19 (95% CI, 0.94-1.49); *P* for trend = .04	NA
Shah et al,[84] 2018	11 376	MDS	NA	• MDS, HR: 0.99 (95% CI, 0.94-1.04)	Race/ethnicity; SES; alcohol intake
		DASH score	NA	• DASH score, HR: 0.94 (95% CI, 0.89-0.99)	NA
Shahar et al,[85] 2009	285	HEI	NA	• HEI: <51, Poor, 1 [Reference]	Physical activity

© 2021 English LK et al. *JAMA Network Open.*

				• HEI: 51-80, Fair, HR: 1.52 (95% CI, 0.7-3.5)<br>• HEI: ≥80, Good, HR: 1.9 (95% CI, 0.7-5.2); *P* for trend = .26	
Shivappa et al,[86] 2017	7627	AHEI 2010	NA	• HR: 0.82 (95% CI, 0.76-0.88); *P* < .001	NA
Shvetsov et al,[87] 2016	193 527	aMED	NA	• aMED (Q5 vs Q1), HR: 0.77 (95% CI, 0.74-0.80)<br>• aMED-e (Q5 vs Q1), HR: 0.79 (95% CI, 0.76-0.82)	NA
Sijtsma et al,[88] 2015	826	DHNaFS	Male participants, with CVD	• T1, 1 [Reference]<br>• T2, HR: 0.58 (95% CI, 0.39-0.86)<br>• T3, HR: 0.67 (95% CI, 0.45-0.99); *P* for trend = .11	Anthropometr y
			Male participants, non-CVD	• ACM:<br>• T1, 1 [Reference]<br>• T2, HR: 1.04 (95% CI, 0.84-1.29)<br>• T3, HR: 0.97 (95% CI, 0.76-1.23); *P* for trend = .82	NA
		DUNaFS	Male participants, with CVD	• T1, 1 [Reference]<br>• T2, HR: 0.98 (95% CI, 0.67-1.42)<br>• T3, HR: 0.79 (95% CI, 0.50-1.24); *P* for trend = .53	NA

© 2021 English LK et al. *JAMA Network Open.*

			Male participants, non-CVD	• T1, 1 [Reference]<br>• T2, HR: 0.81 (95% CI, 0.65-1.00)<br>• T3, HR: 0.86 (95% CI, 0.67-1.10); *P* for trend = .16	NA
Sjögren et al,[89] 2010	924	mMDS	NA	• Per-SD increase, HR: 0.83 (95% CI, 0.70-0.99)<br>• Low 0-2, 1 [Reference]<br>• Medium 3-5, HR: 0.73 (95% CI, 0.52-1.00)<br>• High 6-8, HR: 0.56 (95% CI, 0.33-0.96); *P* for trend = .02	Race/ethnicity
Sotos-Prieto et al,[90] 2017	73 739	aMED	NA	• Q1, HR: 1.06 (95% CI, 0.99-1.13)<br>• Q2, HR: 0.97 (95% CI, 0.91-1.04)<br>• Q3, 1 [Reference]<br>• Q4, HR: 0.93 (95% CI, 0.87-0.98)<br>• Q5, HR: 0.84 (95% CI, 0.78-0.91); *P* for trend <.001	NA
		DASH score	NA	• Q1, HR: 1.06 (95% CI, 1.00-1.12)<br>• Q2, HR: 1.01 (95% CI, 0.94-1.07)<br>• Q3, 1 [Reference]<br>• Q4, HR: 0.93 (95% CI, 0.87-1.00)	NA

© 2021 English LK et al. *JAMA Network Open.*

				• Q5, HR: 0.89 (95% CI, 0.84-0.95); *P* for trend <.001	
		aHEI	NA	• Q1, HR: 1.12 (95% CI, 1.05-1.19)<br>• Q2, HR: 1.06 (95% CI, 1.00-1.13)<br>• Q3, 1 [Reference]<br>• Q4, HR: 0.94 (95% CI, 0.88-1.01)<br>• Q5, HR: 0.91 (95% CI, 0.85-0.97); *P* for trend <.001	NA
Stefler et al,[91] 2017	19 333	mMDS	NA	• Per-SD increase HR: 0.93 (95% CI, 0.88-0.98); *P* for trend = .01<br>• Low 0-7, 1 [Reference]<br>• Moderate 8-10, HR: 0.85 (95% CI, 0.75-0.90)<br>• High 11-17, HR: 0.85 (95% CI, 0.73-1.00); *P* for trend = .03<br>• per 1000 person-years, Low 12.2, Moderate 9.0, High 7.3; *P* < .001	Race/ethnicity; anthropometry
		mMDS	NA	• Per-SD increase, HR: 0.95 (95% CI, 0.90, 1.01); *P* = .11<br>• Low 0-3, 1 [Reference]<br>• Moderate 4-5, HR: 0.90 (95% CI, 0.79-1.02)<br>• High 6-9, HR: 0.88 (95% CI, 0.76-1.03)	NA

© 2021 English LK et al. *JAMA Network Open.*

Struijk et al,[92] 2014	33 066	mMDS	NA	• Per-SD increase, DALY: −0.13 (95% CI, −0.20 to −0.06)<br>• 0-3, 1 [Reference]<br>• 4-5, DALY: −0.16 (95% CI, −0.32 to −0.01)<br>• 6-9, DALY: −0.34 (95% CI, −0.52 to −0.16); *P* for trend = .01	Race/ethnicity
		DHD	NA	• Per-SD increase, DALY: −0.05 (95% CI, −0.11 to 0.01)<br>• T1, [Reference]<br>• T2, DALY: 0.07 (95% CI, −0.09 to 0.23)<br>• T3, DALY: −0.08 (95% CI, −0.25 to −0.09); *P* for trend = .31	NA
		Prudent	NA	• Prudent v. Western Ref, DALY: −0.16 (95% CI, −0.29 to −0.02)<br>• Per-SD increase, DALY: −0.05 (95% CI, −0.14 to −0.04)<br>• T1, 1 [Reference]<br>• T2, DALY: −0.06 (95% CI, −0.22 to −0.09)<br>• T3, DALY: −0.1 (95% CI, −0.34 to −0.1); *P* for trend = .23	NA

		Western	NA	• Prudent v. Western Ref, DALY: −0.16 (95% CI, −0.29 to −0.02)<br>• Per-SD increase, DALY: −0.16 (95% CI, −0.24 to −0.08)<br>• T1, 1 [Reference]<br>• T2, DALY: −0.13 (95% CI, −0.30 to −0.01)<br>• T3, DALY: −0.34 (95% CI, −0.52 to −0.16); *P* for trend <.01	NA
Thorpe et al,[93] 2013	2029	HEI	NA	• 25-44 y, HR: 0.49 (95% CI, 0.14-1.76)<br>• 45-64 y, HR: 1.40 (95% CI, 0.44-4.45)<br>• >65 y, HR: 1.22 (95% CI, 0.48-3.14)	Alcohol intake; physical activity; anthropometry; smoking status
Tognon et al,[94] 2011	1037	Refined MDS	NA	• Continuous, HR: 0.93 (95% CI, 0.89-0.98)<br>• Categorical, highest 4 levels vs others, HR: 0.82 (95% CI, 0.67-0.99)	Race/ethnicity
		aMED, mMDS/HALE, mMDS	NA	• Continuous, HR: 0.97 (95% CI, 0.92-1.02)<br>• Categorical, highest 4 levels vs others, HR: 0.94 (95% CI, 0.79-1.11)	NA

© 2021 English LK et al. *JAMA Network Open.*

Tognon et al,[95] 2012	77 151	MDS	Female participants	• Per-unit, HR: 0.96 (95% CI, 0.92-1.00)<br>• BMI<30, HR: 0.95 (95% CI, 0.91-0.99)<br>• BMI≥30, HR: 0.95 (95% CI, 0.87-1.05)	Race/ethnicity
			Male participants	• Per-unit, HR: 0.96 (95% CI, 0.93-0.99)<br>• BMI<30, HR: 0.95 (95% CI, 0.91-0.98)<br>• BMI≥30, HR: 1.03 (95% CI, 0.95-1.12)	NA
			Female and male participants	• Per-unit, HR: 0.96 (95% CI, 0.93-0.98)<br>• BMI<30, HR: 0.95 (95% CI, 0.92-0.97)<br>• BMI≥30, HR: 0.99 (95% CI, 0.93-1.06)	NA
Tognon et al,[96] 2014	1849	mMDS	NA	• Score 1, HR: 0.95 (95% CI, 0.91-1.00)<br>• Score 2, HR: 0.94 (95% CI, 0.88-0.99)<br>• Score 3, HR: 0.93 (95% CI, 0.87-0.98)	Race/ethnicity

© 2021 English LK et al. *JAMA Network Open.*

Tong et al,[97] 2016	23 902	Literature-based MDS	NA	• HR: 0.97 (95% CI, 0.94-0.99)	Race/ethnicity
		mMDS	NA	• HR: 0.96 (95% CI, 0.93-0.98)	NA
		PyrMDS	NA	• HR: 0.93 (95% CI, 0.93-0.98)<br>• Top 5% PyrMDS, n = 23 902, ACM incidence, 138.4/10 000 person-years, 7.5% cases preventable, PAF: 5.4% (95% CI, 1.3-9.5)<br>• Top 5% PyrMDS at high risk, n = 15 767, incidence, 191.3/10000 person-years, 10.9% cases preventable, PAF: 5.7% (95% CI, 1.6-9.8)<br>• Top 30% PyrMDS, ACM incidence, 138.4/10 000 person-years, 5.2% cases preventable, PAF: 3.8% (95% CI, 0.8-6.8)	NA
		Tertiles of the MDS	NA	• HR: 0.97 (95% CI, 0.94-0.99)	NA
Trichopoulou et al,[98] 2003	22 043	MDS	NA	• Per 2-pt increase, HR: 0.75 (95% CI, 0.64-0.87); P < .001	Race/ethnicity
Trichopoulou et al,[99] 2005	74 607	mMDS	NA	• Per 2-pt increase, HR: 0.93 (95% CI, 0.88-0.99); *P* = .09<br>• Low 0-3, 1 [Reference]	Race/ethnicity

© 2021 English LK et al. *JAMA Network Open.*

				• Middle 4-5, HR: 0.93 (95% CI, 0.87-1.01); *P* for heterogeneity = .74<br>• Highest 6-9, HR: 0.91 (95% CI, 0.82-1.02); *P* for heterogeneity = .38	
Trichopoulou et al,[100] 2009	23 349	MDS	NA	• Per 2-pt increase, HR: 0.86 (95% CI, 0.80-0.93); *P* < .001	Race/ethnicity
van Dam et al,[101] 2008	77 782	AHEI 2010	NA	• Q1, 1 [Reference]<br>• Q2, RR: 0.85 (95% CI, 0.79-0.90)<br>• Q3, RR: 0.80 (95% CI, 0.75-0.85)<br>• Q4, RR: 0.76 (95% CI, 0.71-0.81)<br>• Q5, RR: 0.65 (95% CI, 0.61-0.70)<br>• Q1, Q2, Q3 vs Q4 or Q5, RR: 1.25 (95% CI, 1.19-1.30); PAR: 12.9% (95% CI, 9.6-16.2)	Race/ethnicity; SES
van den Brandt,[102] 2011	120 852	aMED	Female participants	• Per 2-point increase, HR: 0.84 (0.79-0.91); *P* < .001<br>• 0-3, 1 [Reference]<br>• 4-5, HR: 0.80 (95% CI, 0.69-0.93)<br>• 6-9, HR: 0.69 (95% CI, 0.58-0.82)	Race/ethnicity
			Male participants	• Per 2-point increase, HR: 0.94 (95% CI, 0.87-1.02); *P* = .13	NA

© 2021 English LK et al. *JAMA Network Open.*

				• 0-3, 1 [Reference]<br>• 4-5, HR: 0.90 (95% CI, 0.77-1.06)<br>• 6-9, HR: 0.89 (95% CI, 0.74-1.07)	
van Lee et al,[103] 2016	3593	DHD-I	NA	• Per 10-point increment, HR: 0.94 (95% CI, 0.90-0.98)<br>• Q1, 1 [Reference]<br>• Q2, HR: 0.94 (95% CI, 0.82-1.06)<br>• Q3, HR: 0.93 (95% CI, 0.82-1.06)<br>• Q4, HR: 0.81 (95% CI, 0.71-0.93); *P* for trend = .006	Race/ethnicity; anthropometry
Voortman et al,[104] 2017	9701	Dutch dietary guidelines score	NA	• Continuous, HR: 0.97 (95% CI, 0.95-0.98)<br>• Q1, 1 [Reference]<br>• Q2, HR: 0.95 (95% CI, 0.86-1.04)<br>• Q3, HR: 0.93 (95% CI, 0.85-1.02)<br>• Q4, HR: 0.88 (95% CI, 0.80-0.97)<br>• Q5, HR: 0.86 (95% CI, 0.78-0.95); *P* for trend <.001	Race/ethnicity
Vormund et al,[105] 2015	17 861	Classic MDS	Female participants	• HR: 1.00 (95% CI, 0.97-1.04)	Race/ethnicity; physical activity
			Male participants	• HR: 0.96 (95% CI, 0.93-0.98)	NA

© 2021 English LK et al. *JAMA Network Open.*

			Female and male participants	• HR: 0.97 (95% CI, 0.95-1.00)	NA
		aMED, mMDS	Female participants	• HR: 0.96 (95% CI, 0.94-0.98)	NA
			Male participants	• HR: 0.98 (95% CI, 0.95-1.02)	NA
			Female and male participants	• HR: 0.94 (95% CI, 0.92-0.97)	NA
		mMDS	Female participants	• 4-6, HR: 0.90 (95% CI, 0.80-1.02)<br>• 6-9, HR: 0.92 (95% CI, 0.80-1.05)	NA
			Male participants	• 4-6, HR: 0.83 (95% CI, 0.74-0.92)<br>• 6-9, HR: 0.83 (95% CI, 0.73-0.94)	NA
			Female and male participants	• 4-6, HR: 0.86 (95% CI, 0.79-0.93)<br>• 6-9, HR: 0.86 (95% CI, 0.78-0.94)	NA
Wahlqvist et al,[106] 2005	636	MDS	NA	• Reduced death risk of 13% (1%-24%); HR: 0.87 (95% CI, 0.76-0.99)	Physical activity; anthropometry; smoking status
Warensjö Lemming et al,[107] 2018	38 428	aMED	NA	• Per-unit increase, HR: 0.94 (95% CI, 0.92-0.95)<br>• Low, 1 [Reference]<br>• Medium, HR: 0.87 (95% CI, 0.82-0.91)	Age; race/ethnicity; alcohol intake

© 2021 English LK et al. *JAMA Network Open.*

				• High, HR: 0.76 (95% CI, 0.82-0.90)<br>• Per-category, HR: 0.87 (95% CI, 0.8-0.90)	
		HNFI	NA	• Per-unit increase, HR: 1.00 (95% CI, 0.99-1.02)<br>• Low, 1 [Reference]<br>• Medium, HR: 0.96 (95% CI, 0.91-1.00)<br>• High, HR: 0.98 (95% CI, 0.91-1.06)<br>• Per category, HR: 0.98 (95% CI, 0.95-1.02)	NA
Whalen et al,[108] 2017	21423	MedDietScore	NA	• Q1, 1 [Reference]<br>• Q2, HR: 0.90 (95% CI, 0.80-1.02)<br>• Q3, HR: 0.82 (95% CI, 0.72-0.92)<br>• Q4, HR: 0.79 (95% CI, 0.69-0.90)<br>• Q5, HR: 0.64 (95% CI, 0.55-0.74)	Alcohol intake
		Paleolithic diet score	NA	• Q1, 1 [Reference]<br>• Q2, HR: 0.95 (95% CI, 0.84-1.08)<br>• Q3, HR: 0.94 (95% CI, 0.83-1.07)<br>• Q4, HR: 0.87 (95% CI, 0.77-0.99)<br>• Q5, HR: 0.77 (95% CI, 0.67-0.89)	NA

© 2021 English LK et al. *JAMA Network Open.*

Yu et al,[109] 2015	77 572	HEI 2010	NA	• Q1, 1 [Reference]<br>• Q2, HR: 0.99 (95% CI, 0.92-1.06)<br>• Q3, HR: 0.95 (95% CI, 0.89-1.03)<br>• Q4, HR: 0.93 (95% CI, 0.86-1.00)<br>• Q5, HR: 0.80 (95% CI, 0.73-0.86); *P* for trend <.001	NA
Zaslavsky et al,[110] 2017	10 431	DASH score	NA	• Q1, 1 [Reference]<br>• Q2, HR: 0.97 (95% CI, 0.88-1.07)<br>• Q3, HR: 0.95 (95% CI, 0.86-1.05)<br>• Q4, HR: 0.88 (95% CI, 0.79-0.98); *P* for trend = .02	Alcohol intake
		aMED	NA	• Q1, 1 [Reference]<br>• Q2, HR: 0.98 (95% CI, 0.89-1.08)<br>• Q3, HR: 0.91 (95% CI, 0.81-1.03)<br>• Q4, HR: 0.86 (95% CI, 0.76-0.97); *P* for trend = .006	NA
Zaslavsky et al,[111] 2018	10 431	aMED	NA	• Per-unit increase, HR (95% CI): 0.96 (0.943 to 0.985), *P* = .001	NA
Anderson et al,[112] 2011	2582	Healthy foods[e]	NA	• 1 [Reference]	Anthropometr y
		High-fat dairy products[e]	NA	• RR: 1.40 (95% CI, 1.04-1.88)	NA
		Meat, fried foods, and alcohol[e]	NA	• RR: 1.21 (95% CI, 0.92-1.60)	NA

© 2021 English LK et al. *JAMA Network Open.*

		Breakfast cereal[e]	NA	• RR: 1.16 (95% CI, 0.86-1.56)	NA
		Refined grains[e]	NA	• RR: 1.08 (95% CI, 0.80-1.45)	NA
		Sweets and desserts[e]	NA	• RR: 1.37 (95% CI, 1.02-1.86)	NA
Atkins et al,[113] 2016	3226	High-fat/low-fiber[e]	NA	• Q1, 1 [Reference]<br>• Q2, HR: 1.1 (95% CI, 0.88-1.38)<br>• Q3, HR: 1.11 (95% CI, 0.88-1.39)<br>• Q4, HR: 1.44 (95% CI, 1.13-1.84); *P* for trend = .007<br>• Rate/1000 person-years, Q1: 22.65; Q2: 24.62; Q3: 30.59; and Q4: 35.69	NA
		Prudent[e]	NA	• Q1, 1 [Reference]<br>• Q2, HR: 0.77 (95% CI, 0.63-0.95)<br>• Q3, HR: 0.93 (95% CI, 0.75-1.14)<br>• Q4, HR: 0.83 (95% CI, 0.66-1.04); *P* for trend = .28<br>• Rate/1000 person-years, Q1: 36.66; Q2: 26.64; Q3: 25.15; and Q4: 24.97	NA
		High sugar[e]	NA	• Q1, 1 [Reference]<br>• Q2, HR: 1.06 (95% CI, 0.85-1.31)<br>• Q3, HR: 0.91 (95% CI, 0.72-1.15)	NA

© 2021 English LK et al. *JAMA Network Open.*

				• Q4, HR: 1.0 (95% CI, 0.77-1.29); *P* for trend = .71<br>• Rate/1000 person-years, Q1: 27.32; Q2: 28; Q3: 26.56; and Q4: 31.18	
Bamia et al,[114] 2007	74 607	Plant-based[e]	NA	• Per SD, HR (95% CI): 0.86, (0.77 to 0.95), *P* = .06<br>• T1, [Ref]<br>• T2, HR (95% CI): 0.90 (0.84 to 0.98): *P* = .502;<br>• T3, HR: 0.89 (95% CI, 0.79-0.99); *P* = .12	Race/ethnicity
Brunner et al,[115] 2008	7731	Unhealthy[e]	NA	• 1 [Reference]	NA
		Sweet[e]	NA	• HR: 0.90 (95% CI, 0.63-1.27); P = .55	NA
		Mediterranean-like[e]	NA	• HR: 0.81 (95% CI, 0.57-1.15); P = .23	NA
		Healthy[e]	NA	• HR: 0.95 (95% CI, 0.74-1.22); P = .69	NA
Granic et al,[116] 2013	12 830	Moderate intake and starch diet[e]	NA	• ACM≥20+ y past baseline, HR: 1.09 (95% CI, 1.02-1.17); *P* = .01<br>• ACM at follow-up 10+ y past baseline, HR: 1.05 (95% CI, 0.99-1.12); *P* = .13	Race/ethnicity

© 2021 English LK et al. *JAMA Network Open.*

		Moderate intake with low flour-based food diet[e] [reference]	NA	• 1 [Reference]	
		Meat and starch diet[e]	NA	• ACM≥20+ y past baseline, HR: 1.07 (95% CI, 1.00-1.14); *P* = .054<br>• ACM 10+ y past baseline, HR: 1.04 (95% CI, 0.98-1.11); *P* = .20	NA
		Low meat intake diet[e]	NA	• ACM 10+ y past baseline, HR: 1.02 (95% CI, 0.96-1.08); *P* = .47<br>• ACM 20+ y past baseline, HR: 1.03 (95% CI, 0.97-1.10); *P* = .39	NA
Hamer et al,[117] 2010	1017	Mediterranean[e]	NA	• T1, 1 [Reference]<br>• T2, HR: 0.81 (95% CI, 0.67-0.97)<br>• T3, HR: 0.82 (95% CI, 0.68-1.00); *P* for trend = .04	Race/ethnicity
		Health aware[e]	NA	• T1, 1 [Reference]<br>• T2, HR: 1.04 (95% CI, 0.86-1.25)<br>• T3, HR: 0.93 (95% CI, 0.76-1.13); *P* for trend = .53	NA
		Traditional[e]	NA	• T1, 1 [Reference]<br>• T2, HR: 0.94 (95% CI, 0.78-1.15)<br>• T3, HR: 1.15 (95% CI, 0.94-1.40); *P* for trend = .14	NA

© 2021 English LK et al. *JAMA Network Open.*

		Sweet and fat[e]	NA	• T1, 1 [Reference]<br>• T2, HR: 1.02 (95% CI, 0.84-1.24)<br>• T3, HR: 0.93 (95% CI, 0.75-1.15); P for trend = .62	NA
Heidemann et al,[118] 2008	72 113	Prudent[e]	NA	• Q1, 1 [Reference]<br>• Q2, RR: 0.85 (95% CI, 0.78-0.92)<br>• Q3, RR: 0.84 (95% CI, 0.78-0.91)<br>• Q4, RR: 0.81 (95% CI, 0.74-0.88)<br>• Q5, RR: 0.83 (95% CI, 0.76-0.90); P for trend <.001	Race/ethnicity; alcohol intake
		Western[e]	NA	• Q1, 1 [Reference]<br>• Q2, RR: 1.00 (95% CI, 0.92-1.08)<br>• Q3, RR: 1.10 (95% CI, 1.02-1.20)<br>• Q4, RR: 1.16 (95% CI, 1.06-1.26)<br>• Q5, RR: 1.21 (95% CI, 1.21-1.32); P for trend <.001	NA
Hoffmann et al,[119] 2005	9356	PCA Pattern 1	NA	• Per-SD increase, RR: 1.10 (95% CI, 0.96-1.28)<br>• Q1, 1 [Reference]<br>• Q2, RR: 0.82 (95% CI, 0.57-1.22)<br>• Q3, RR: 1.00 (95% CI, 0.70-1.45)<br>• Q4, RR: 1.03 (95% CI, 0.70-1.51)	Race/ethnicity

© 2021 English LK et al. *JAMA Network Open.*

				• Q5, RR: 1.06 (95% CI, 0.68-1.65); P for trend = .50	
		PCA Pattern 2	NA	• Per-SD increase, RR: 0.99 (95% CI, 0.89-1.10)<br>• Q1, 1 [Reference]<br>• Q2, RR: 0.91 (95% CI, 0.68-1.22)<br>• Q3, RR: 0.90 (95% CI, 0.66-1.23)<br>• Q4, RR: 1.10 (95% CI, 0.81-1.51)<br>• Q5, RR: 0.80 (95% CI, 0.55-1.15); P for trend = .61	NA
		RRR Pattern 1	NA	• Per-SD increase, RR: 1.20 (95% CI, 1.09-1.31)<br>• Q1, 1 [Reference]<br>• Q2, RR: 1.10 (95% CI, 0.70-1.46)<br>• Q3, RR: 0.96 (95% CI, 0.66-1.38)<br>• Q4, RR: 1.32 (95% CI, 0.95-1.85)<br>• Q5, RR: 1.61 (95% CI, 1.17-2.21); P for trend = <.001	NA
		RRR Pattern 2	NA	• Per-SD increase, RR: 0.96 (95% CI, 0.87-1.06)<br>• Q1, 1 [Reference]<br>• Q2, RR: 0.87 (95% CI, 0.63-1.21)<br>• Q3, RR: 0.81 (95% CI, 0.57-1.13)	NA

				• Q4, RR:1.07 (95% CI, 0.78-1.48)<br>• Q5, RR: 0.96 (95% CI, 0.70-1.33); P for trend = .74	
Hsiao et al,[120] 2013	446	Sweets and dairy[e]	NA	• OR: 1.02 (95% CI, 0.64-1.63)	Race/ethnicity; physical activity (n = 179 missing data)
		Western[e]	NA	• OR: 0.95 (95% CI, 0.55-1.63)	
		Health conscious[e]	NA	• 1 [Reference]	
Krieger et al,[121] 2018	15 936	Sausage and vegetables[e]	Female participants	• 1 [Reference]	NA
			Male participants	• 1 [Reference]	NA
			Female and male participants	• 1 [Reference]	NA
		Meat and salad[e]	Female participants	• HR: 0.93 (95% CI, 0.80-1.08)	NA
			Male participants	• HR: 0.95 (95% CI, 0.85-1.07)	NA
			Female and male participants	• HR: 0.94 (95% CI, 0.86-1.03)	NA
		Fish[e]	Female participants	• HR: 0.98 (95% CI, 0.83-1.15)	NA
			Male participants	• HR: 0.82 (95% CI, 0.71-0.96)	NA

© 2021 English LK et al. *JAMA Network Open.*

			Female and male participants	• HR: 0.87 (95% CI, 0.78-0.97)	NA
		Traditional[e]	Female participants	• HR: 1.02 (95% CI, 0.87-1.19)	NA
			Male participants	• HR: 0.81 (95% CI, 0.71-0.93)	NA
			Female and male participants	• HR: 0.89 (95% CI, 0.80-0.98)	NA
		High-fiber foods[e]	Female participants	• HR: 0.91 (95% CI, 0.79-1.05)	NA
			Male participants	• HR: 0.94 (95% CI, 0.83-1.08)	NA
			Female and male participants	• HR: 0.92 (95% CI, 0.84-1.02)	NA
Martínez-González et al,[122] 2015	7216	Western[e]	NA	• Q1, 1 [Reference]<br>• Q2, HR: 0.93 (95% CI, 0.66-1.3)<br>• Q3, HR: 1.05 (95% CI, 0.75-1.46)<br>• Q4, HR: 1.04 (95% CI, 0.74-1.47); P for trend = .65	Race/ethnicity
		Mediterranean[e]	NA	• Q1, 1 [Reference]<br>• Q2, HR: 0.82 (95% CI, 0.62-1.10)<br>• Q3, HR: 0.74 (95% CI, 0.54-0.99)<br>• Q4, HR: 0.53 (95% CI, 0.38-0.75); P for trend <.001	NA

© 2021 English LK et al. *JAMA Network Open.*

Masala et al,[123] 2007	5611	Prudent[e]	NA	• Q1, 1 [Reference]<br>• Q2, HR: 0.99 (95% CI, 0.63-1.54)<br>• Q3, HR: 0.93 (95% CI, 0.58-1.51)<br>• Q4, HR: 0.47 (95% CI, 0.47-1.53); P for trend = .59	Race/ethnicity
		Pasta and meat[e]	NA	• Q1, 1 [Reference]<br>• Q2, HR: 1.07 (95% CI, 0.67-1.70)<br>• Q3, HR: 0.99 (95% CI, 0.59-1.64)<br>• Q4, HR: 1.37 (95% CI, 0.80-2.34); P for trend = .34	NA
		Olive oil and salad[e]	NA	• Q1, 1 [Reference]<br>• Q2, HR: 0.78 (95% CI, 0.50-1.21)<br>• Q3, HR: 0.76 (95% CI, 0.48-1.20)<br>• Q4, HR: 0.50 (95% CI, 0.29-0.86); P for trend = .02	NA
		Sweets and dairy[e]	NA	• Q1, 1 [Reference]<br>• Q2, HR: 0.90 (95% CI, 0.56-1.45)<br>• Q3, HR: 0.87 (95% CI, 0.52-1.45)<br>• Q4, HR: 0.85 (95% CI, 0.85-2.54); P for trend = .25	NA
Menotti et al,[124] 2012	1221	Factor 1[e]	NA	• HR: 1.00 (95% CI, 0.94-1.06)	Age; race/ethnicity; SES; physical

© 2021 English LK et al. *JAMA Network Open.*

					activity; smoking status
		Factor 2[e]	NA	• HR: 0.89 (95% CI, 0.83-0.96); P value NR	
		Factor 3[e]	NA	• HR: 0.93 (95% CI, 0.97-1.00)	
Menotti et al,[125] 2014	1564	Factor 2[e]	NA	• 20 y follow-up, diet score 1, HR: 1.42 (95% CI, 1.18-1.71)<br>• 40 y follow-up, diet score 1, HR: 1.31 (95% CI, 1.15-1.50)<br>• 20 y follow-up, diet score 2, HR: 0.99 (95% CI, 0.81-1.21)<br>• 40 y follow-up, diet score 2, HR: 0.98 (95% CI, 0.86-1.11)	Race/ethnicity; SES; physical activity; anthropometry; smoking status
Menotti et al,[110] 2016	1712	Factor 2,[e] Q5 vs Q1 [reference: Q1]	NA	• HR: 0.87; P < .05	Race/ethnicity; SES; physical activity; anthropometry; smoking status
		Non–Mediterranean diet[e] [reference: Q1]	NA	• 1 [Reference]	
		Prudent [Q2, Q3, and Q4]	NA	• Life-years gained, HR: 2.76 (95% CI, 1.48-4.04)	NA
		Mediterranean diet[e] [reference: Q1]	NA	• Life-years gained, HR: 4.36 (95% CI, 2.79-5.92)	NA

© 2021 English LK et al. *JAMA Network Open.*

Nanri et al,[127] 2017	81 720	Prudent[e]	NA	• Q1, 1 [Reference]<br>• Q2, HR: 0.89 (95% CI, 0.84-0.94)<br>• Q3, HR: 0.81 (95% CI, 0.77-0.85)<br>• Q4, HR: 0.82 (95% CI, 0.77-0.86); P for trend <.001	SES
		Westernized[e]	NA	• Q1, 1 [Reference]<br>• Q2, HR: 0.93 (95% CI, 0.89-0.98)<br>• Q3, HR: 0.88 (95% CI, 0.84-0.93)<br>• Q4, HR: 0.91 (95% CI, 0.85-0.96); P for trend <.001	NA
		Traditional Japanese[e]	NA	• Q1, 1 [Reference]<br>• Q2, HR: 0.94 (95% CI, 0.89-1.0)<br>• Q3, HR: 0.93 (95% CI, 0.87-0.99)<br>• Q4, HR: 0.97 (95% CI, 0.91-1.03); P for trend = .49	NA
Odegaard et al,[128] 2014	52 584	Vegetable-, fruit-, and soy-rich[e]	NA	• Q1, 1 [Reference]<br>• Q2, HR: 0.90 (95% CI, 0.84-0.94)<br>• Q3, HR: 0.79 (95% CI, 0.74-0.84)<br>• Q4, HR: 0.80 (95% CI, 0.75-0.85)<br>• Q5, HR: 0.75 (95% CI, 0.70-0.80); P for trend <.001	NA

© 2021 English LK et al. *JAMA Network Open.*

		Dim sum– and meat- rich[e]	NA	• Q1, 1 [Reference]<br>• Q2, HR: 0.98 (95% CI, 0.92-1.04)<br>• Q3, HR: 1.01 (95% CI, 0.95-1.08)<br>• Q4, HR: 1.06 (95% CI, 0.99-1.13)<br>• Q5, HR: 1.14 (95% CI, 1.06-1.23); P for trend <.001	NA
Waijers et al,[129] 2006	5427	Mediterranean-like[e]	NA	• HR: 1.25 (95% CI, 0.52-0.95)<br>• T1, 1 [Reference]<br>• T2, HR: 0.91<br>• T3, HR: 0.84	NA
		Traditional Dutch dinner[e]	NA	• T1, 1 [Reference]<br>• T2, HR: 1.00<br>• T3, HR: 1.25	NA
		Healthy traditional[e]	NA	• T1, 1 [Reference]<br>• T2, HR: 0.81	NA
Zazpe et al,[130] 2014	16 008	Western[e]	NA	• T1, 1 [Reference]<br>• T2, HR: 0.94 (95% CI, 0.61-1.44)<br>• T3, HR: 0.79 (95% CI, 0.45-1.38); P for trend = .40	Race/ethnicity (all Spanish)
		Mediterranean[e]	NA	• T1, 1 [Reference]	NA

© 2021 English LK et al. *JAMA Network Open.*

				• T2, HR: 0.72 (95% CI, 0.48-1.08)<br>• T3, HR: 0.53 (95% CI, 0.34-0.84); P for trend = .01	
		Alcoholic beverages[e]	NA	• T1, 1 [Reference]<br>• T2, HR: 0.99 (95% CI, 0.64-1.56)<br>• T3, HR: 0.78 (95% CI, 0.48-1.27); P for trend = .27	NA
Zhao et al,[131] 2019	2949	Meat-fat pattern[e]	NA	• T1, 1 [Reference]<br>• T2, HR: 1.21 (95% CI, 0.86-1.69)<br>• T3, HR: 1.25 (95% CI, 0.84-1.88); P for trend = .27	NA
		Healthy pattern[e]	NA	• T1, 1 [Reference]<br>• T2, HR: 0.64 (95% CI, 0.47-0.88); P for trend = .051<br>• T3, HR: 0.74 (95% CI, 0.53-1.02)	NA
		Dairy-bread pattern[e]	NA	• T1, 1 [Reference]<br>• T2, HR: 0.95 (95% CI, 0.69-1.30)<br>• T3, HR: 1.34 (95% CI, 0.98-1.83); P for trend = .08	NA
Mihrshahi et al,[132] 2017	243 096	Vegetarian[e]	NA	• Vegetarian vs nonvegetarian, HR: 1.16 (95% CI, 0.93-1.45)	NA

© 2021 English LK et al. *JAMA Network Open.*

				• Vegetarian vs regular meat eater, HR: 1.16 (95% CI, 0.93-1.45)<br>• P for overall effect of diet category = .10	
		Semivegetarian[e]	NA	• HR: 1.12 (95% CI, 0.96-1.31)	NA
		Pescovegetarian[e]	NA	• HR: 0.79 (95% CI, 0.59-1.06)	NA
		Regular meat eater[e]	NA	• 1 [Reference]	NA
Song et al,[133] 2016	131 342	Animal protein[e]	NA	• ≤10%, 1 [Reference]<br>• >10% to ≤12%, HR: 1.01 (95% CI, 0.97-1.05)<br>• >12% to ≤15%, HR: 1.03 (95% CI, 0.99-1.07)<br>• >15% to ≤18%, HR: 1.03 (95% CI, 0.98-1.07)<br>• >18%, HR: 1.03 (95% CI, 0.98-1.08)<br>• Per-10% increment, HR: 1.02 (95% CI, 0.98-1.05); P for trend = .33	Race/ethnicity; SES
		Plant protein[e]	NA	• ≤3%, 1 [Reference]<br>• >3% to ≤4%, HR: 0.97 (95% CI, 0.94-1.01)	NA

© 2021 English LK et al. *JAMA Network Open.*

				• >4% to ≤5%, HR: 0.95 (95% CI, 0.91-0.99)<br>• >5% to ≤6%, HR: 0.91 (95% CI, 0.86-0.96)<br>• >6%, HR: 0.89 (95% CI, 0.84-0.96)<br>• Per-3% increment, HR: 0.90 (95% CI, 0.86-0.95); P < .001	
Key et al,[134] 2009	47 254	• Meat eater[e]<br>• Fish eater[e]<br>• Vegetarian, including vegan[e]<br>• Vegan[e]<br>• Nonvegetarian, including meate eater and fish eater	NA	• Full sample, SMR:<br>• Nonvegetarian, SMR: 52 (50-54)<br>• Vegetarian, SMR: 52 (48-56)	Race/ethnicity; SES; physical activity; anthropometry
				• In those without previous disease,<br>• Nonvegetarian, 1 [Reference]<br>• Vegetarian, DRR: 1.05 (95% CI, 0.93-1.19)<br>• Heterogeneity P = .44	
				• Meat eater, 1 [Reference]<br>• Fish eater, DRR: 0.89 (95% CI, 0.75-1.05)<br>• Vegetarian or vegan, DRR: 1.03 (95% CI, 0.9-1.16)	
				• Heterogeneity P = .28	

© 2021 English LK et al. *JAMA Network Open.*

Orlich et al,[135] 2013	73 308	• Nonvegetarian[e]<br>• Semivegetarian[e]<br>• Pescovegetarian[e]<br>• Lacto-ovo vegetarian[e]<br>• Vegan[e]	Female participants	• Nonvegetarian, HR, 1 [Reference]<br>• Vegan, HR: 0.97 (95% CI, 0.78-1.20)<br>• Lacto-ovo vegetarian, HR: 0.94 (95% CI, 0.83-1.07)<br>• Pescovegetarian, HR: 0.88 (95% CI, 0.72-1.07)<br>• Semivegetarian, HR: 0.92 (95% CI, 0.70-1.22)<br>• Vegetarian, HR: 0.93 (95% CI, 0.82-1.05)	NA
		• Nonvegetarian[e]<br>• Semivegetarian[e]<br>• Pescovegetarian[e]<br>• Lacto-ovo vegetarian[e]	Male participants	• Nonvegetarian, 1 [Reference]<br>• Vegan, HR: 0.72 (95% CI, 0.5-0.92)<br>• Lacto-ovo vegetarian, HR: 0.86 (95% CI, 0.74-1.01)<br>• Pescovegetarian, HR: 0.73 (95% CI, 0.57-0.93)<br>• Semivegetarian, HR: 0.93 (95% CI, 0.68-1.26)<br>• Vegetarian, HR: 0.82 (95% CI, 0.72-0.94)	NA

© 2021 English LK et al. *JAMA Network Open.*

		• Vegan[e]			
		• Nonvegetarian[e]<br>• Semivegetarian[e]<br>• Pescovegetarian[e]<br>• Lacto-ovo vegetarian[e]<br>• Vegan[e]	Female and male participants	• Nonvegetarian, 1 [Reference]<br>• Vegan, HR: 0.85 (95% CI, 0.73-1.01)<br>• Lacto-ovo vegetarian, HR: 0.91 (95% CI, 0.82-1.00)<br>• Pescovegetarian, HR: 0.81 (95% CI, 0.69-0.94)<br>• Semivegetarian, HR: 0.92 (95% CI, 0.75-1.13)<br>• Vegetarian, HR: 0.88 (95% CI, 0.80-0.97)	NA
Chang-Claude et al,[136] 2005	1724	• Vegetarian[e]<br>• Lacto-ovo vegetarian[e]<br>• Nonvegetarian[e]	NA	• Vegetarian, SMR: 62 (56-69)<br>• Nonvegetarian, SMR: 52 (44-61)	NA
				• Nonvegetarian, 1 [Reference]<br>• Vegetarian, RR: 1.10 (95% CI, 0.89-1.36)	
Héroux et al,[137] 2010	13 621	RRR	NA	• Q1, 1 [Reference]<br>• Q2, HR: 1.05 (95% CI, 0.80-1.37)	Anthropometry; SES

© 2021 English LK et al. *JAMA Network Open.*

				• Q3, HR: 1.03 (95% CI, 0.78-1.36)<br>• Q4, HR: 0.96 (95% CI, 0.70-1.31)<br>• Q5, HR: 1.18 (95% CI, 0.86-1.64)	
Meyer et al,[138] 2011	981	RRR	NA	• RRR, HR: 1.16 (95% CI, 1.0-1.33); P = .046	Race/ethnicity
		PLS	NA	• PLS, HR: 1.18 (95% CI, 1.02-1.37); P = .03	
		PCR	NA	• PCR, HR: 1.16 (95% CI, 1.00-1.35); P = .054	
Schnabel et al,[139] 2019	44 551	Ultraprocessed[e]	NA	• Per-10% increment, HR: 1.14 (95% CI, 1.0-1.27); P for trend = .008	Race/ethnicity
Kim et al,[140] 2019	11 898	Ultraprocessed[e]	NA	• Q1, 1 [Reference]<br>• Q2, HR: 0.99 (95% CI, 0.83-1.18)<br>• Q3, HR: 1.06 (95% CI, 0.87-1.30)<br>• Q4, HR: 1.30 (95% CI, 1.08-1.57); P for trend <.001	NA
Rico-Campà et al,[141] 2019	19 899	Ultraprocessed[e]	NA	• Low, 1 [Reference]<br>• High, HR: 1.62 (95% CI, 1.13-2.33): P for trend = .005<br>• Medium-low, HR: 1.06 (95% CI, 0.76-1.48)	Race/ethnicity (all Spanish)

© 2021 English LK et al. *JAMA Network Open.*

				• Medium-high, HR: 1.38 (95% CI, 0.99-1.92)	

Abbreviations: ACM, all-cause mortality; ADI, area deprivation index; AHEI, alternative healthy eating index; AICR, American Institute for Cancer Research; AIDI, anti-inflammatory diet index; aMED, alternate Mediterranean diet score; aMED-e, energy-standardized aMED; arMED, adapted relative Mediterranean diet score; BMI, body mass index; CVD, cardiovascular disease; DALY, disability-adjusted life years; DASH, Dietary Approaches to Stop Hypertension; DBS, dietary behavior score; DHD, Dutch Healthy Diet; DHD-I, Dutch Healthy Diet index; DHNaFS, Dutch Healthy Nutrient and Food Score; DQI, Diet Quality Index; DQI-I, DQI-International; DQI-K, DQI for Koreans; DQI-SNR, DQI–Swedish Nutrition Recommendations; DRR, death rate ratio; DS, DASH score; DST, dietary screening tool; DUNaFS, Dutch Undesirable Nutrient and Food Score; HALE, Healthy Aging: a Longitudinal Study in Europe; HEI, Healthy Eating Index; HLI, healthy lifestyle index; HNFI, Healthy Nordic Food Index; hPDI, healthful plant-based diet index; HR, hazard ratio; ITT, intention to treat; MAI, Mediterranean Adequacy Index; MDS, Mediterranean diet score; MEDAS, Mediterranean Diet Adherence Screener; MedDietScore, Mediterranean-based diet score; mMDS, modified MDS; MRR, mortality rate ratio; NA, not applicable or not available; NR, not reported; NRFS, nonrecommended food score; OR, odds ratio; PAF, population attributable fraction; PAR, population attributable risk; PCA, principal component analysis; PCR, principal components regression; PD, percentile difference; PDI, plant-based diet index; PLS, partial least squares regression; PyrMDS, Mediterranean Diet Pyramid Score; Q, quintile or quartile; *R*, correlation coefficient; RFBS, recommended food and behavior score; RFS, recommended food score; RR, relative risk or risk ratio; RRR, reduced rank regression; SES, socioeconomic status; SMR, standardized mortality ratio; uPDI, unhealthful PDI; WCRF, World Cancer Research Fund.

[b]Adapted from the 2020 Dietary Guidelines Advisory Committee and Nutrition Evidence Systematic Review Team.[142]

[c]Analytic sample size.

[d]Key confounders are identified in Figure 1. This table lists any key confounders that were not accounted for either by design or by analysis to further illustrate that most studies accounted for most key confounders. All key confounders or other factors considered that were adjusted for in each study are included in the tables from the complete review that is available online.[142]

[e]The name or label of the dietary pattern was assigned by the authors of the article.

© 2021 English LK et al. *JAMA Network Open.*

eReferences

1. Estruch R, Ros E, Salas-Salvadó J, et al; PREDIMED Study Investigators. Primary prevention of cardiovascular disease with a Mediterranean diet supplemented with extra-virgin olive oil or nuts. *N Engl J Med*. 2018;378(25):e34.
2. Abe S, Zhang S, Tomata Y, Tsuduki T, Sugawara Y, Tsuji I. Japanese diet and survival time: the Ohsaki cohort 1994 study. *Clin Nutr*. 2020;39(1):298-303.
3. Akbaraly TN, Ferrie JE, Berr C, et al. Alternative Healthy Eating Index and mortality over 18 y of follow-up: results from the Whitehall II cohort. *Am J Clin Nutr*. 2011;94(1):247-253.
4. Al Rifai M, Greenland P, Blaha MJ, et al. Factors of health in the protection against death and cardiovascular disease among adults with subclinical atherosclerosis. *Am Heart J*. 2018;198:180-188.
5. Atkins JL, Whincup PH, Morris RW, Lennon LT, Papacosta O, Wannamethee SG. High diet quality is associated with a lower risk of cardiovascular disease and all-cause mortality in older men. *J Nutr*. 2014;144(5):673-680.
6. Baden MY, Liu G, Satija A, et al. Changes in plant-based diet quality and total and cause-specific mortality. *Circulation*. 2019;140(12):979-991.
7. Behrens G, Fischer B, Kohler S, Park Y, Hollenbeck AR, Leitzmann MF. Healthy lifestyle behaviors and decreased risk of mortality in a large prospective study of U.S. women and men. *Eur J Epidemiol*. 2013;28(5):361-372.
8. Bellavia A, Tektonidis TG, Orsini N, Wolk A, Larsson SC. Quantifying the benefits of Mediterranean diet in terms of survival. *Eur J Epidemiol*. 2016;31(5):527-530.
9. Biesbroek S, Verschuren WMM, Boer JMA, et al. Does a better adherence to dietary guidelines reduce mortality risk and environmental impact in the Dutch sub-cohort of the European Prospective Investigation into Cancer and Nutrition? *Br J Nutr*. 2017;118(1):69-80.
10. Bittoni MA, Wexler R, Spees CK, Clinton SK, Taylor CA. Lack of private health insurance is associated with higher mortality from cancer and other chronic diseases, poor diet quality, and inflammatory biomarkers in the United States. *Prev Med*. 2015;81:420-426.

© 2021 English LK et al. *JAMA Network Open.*

11. Bo S, Ponzo V, Goitre I, et al. Predictive role of the Mediterranean diet on mortality in individuals at low cardiovascular risk: a 12-year follow-up population-based cohort study. *J Transl Med*. 2016;14:91.
12. Boggs DA, Ban Y, Palmer JR, Rosenberg L. Higher diet quality is inversely associated with mortality in African-American women. *J Nutr*. 2015;145(3):547-554.
13. Bonaccio M, Di Castelnuovo A, Costanzo S, et al. Mediterranean diet and mortality in the elderly: a prospective cohort study and a meta-analysis. *Br J Nutr*. 2018;120(8):841-854.
14. Bongard V, Arveiler D, Dallongeville J, et al. Food groups associated with a reduced risk of 15-year all-cause death. *Eur J Clin Nutr*. 2016;70(6):715-722.
15. Booth JN III, Colantonio LD, Howard G, et al. Healthy lifestyle factors and incident heart disease and mortality in candidates for primary prevention with statin therapy. *Int J Cardiol*. 2016;207:196-202.
16. Brown JC, Harhay MO, Harhay MN. Physical activity, diet quality, and mortality among community-dwelling prefrail and frail older adults. *J Nutr Gerontol Geriatr*. 2016;35(4):253-266.
17. Buckland G, Agudo A, Travier N, et al. Adherence to the Mediterranean diet reduces mortality in the Spanish cohort of the European Prospective Investigation into Cancer and Nutrition (EPIC-Spain). *Br J Nutr*. 2011;106(10):1581-1591.
18. Cárdenas-Fuentes G, Subirana I, Martinez-Gonzalez MA, et al. Multiple approaches to associations of physical activity and adherence to the Mediterranean diet with all-cause mortality in older adults: the Prevención con Dieta Mediterránea study. *Eur J Nutr*. 2019;58(4):1569-1578.
19. Chan RSM, Yu BWM, Leung J, et al. How dietary patterns are related to inflammaging and mortality in community-dwelling older Chinese adults in Hong Kong - a prospective analysis. *J Nutr Health Aging*. 2019;23(2):181-194.
20. Cheng E, Um CY, Prizment A, Lazovich D, Bostick RM. Associations of evolutionary-concordance diet, Mediterranean diet and evolutionary-concordance lifestyle pattern scores with all-cause and cause-specific mortality. *Br J Nutr*. 2018;1-10.

© 2021 English LK et al. *JAMA Network Open.*

21. Chrysohoou C, Pitsavos C, Lazaros G, Skoumas J, Tousoulis D, Stefanadis C; Ikaria Study Investigators. Determinants of all-cause mortality and incidence of cardiovascular disease (2009 to 2013) in older adults: the Ikaria Study of the Blue Zones. *Angiology*. 2016;67(6):541-548.
22. Cuenca-García M, Artero EG, Sui X, Lee DC, Hebert JR, Blair SN. Dietary indices, cardiovascular risk factors and mortality in middle-aged adults: findings from the Aerobics Center Longitudinal Study. *Ann Epidemiol*. 2014;24(4):297-303.e2.
23. Dai J, Krasnow RE, Reed T. Midlife moderation-quantified healthy diet and 40-year mortality risk from CHD: the prospective National Heart, Lung, and Blood Institute Twin Study. *Br J Nutr*. 2016;116(2):326-334.
24. Drake I, Gullberg B, Sonestedt E, et al. Scoring models of a diet quality index and the predictive capability of mortality in a population-based cohort of Swedish men and women. *Public Health Nutr*. 2013;16(3):468-478.
25. Ford ES, Zhao G, Tsai J, Li C. Low-risk lifestyle behaviors and all-cause mortality: findings from the National Health and Nutrition Examination Survey III Mortality Study. *Am J Public Health*. 2011;101(10):1922-1929.
26. Ford ES, Bergmann MM, Boeing H, Li C, Capewell S. Healthy lifestyle behaviors and all-cause mortality among adults in the United States. *Prev Med*. 2012;55(1):23-27.
27. Ford DW, Hartman TJ, Still C, et al. Body mass index, poor diet quality, and health-related quality of life are associated with mortality in rural older adults. *J Nutr Gerontol Geriatr*. 2014;33(1):23-34.
28. Fresán U, Sabaté J, Martínez-Gonzalez MA, Segovia-Siapco G, de la Fuente-Arrillaga C, Bes-Rastrollo M. Adherence to the 2015 Dietary Guidelines for Americans and mortality risk in a Mediterranean cohort: the SUN project. *Prev Med*. 2019;118:317-324.
29. George SM, Ballard-Barbash R, Manson JE, et al. Comparing indices of diet quality with chronic disease mortality risk in postmenopausal women in the Women's Health Initiative Observational Study: evidence to inform national dietary guidance. *Am J Epidemiol*. 2014;180(6):616-625.
30. Harmon BE, Boushey CJ, Shvetsov YB, et al. Associations of key diet-quality indexes with mortality in the multiethnic

© 2021 English LK et al. *JAMA Network Open.*

cohort: the Dietary Patterns Methods Project. *Am J Clin Nutr*. 2015;101(3):587-597.
31. Hashemian M, Farvid MS, Poustchi H, et al. The application of six dietary scores to a Middle Eastern population: a comparative analysis of mortality in a prospective study. *Eur J Epidemiol*. 2019;34(4):371-382.
32. Haveman-Nies A, de Groot L, Burema J, Cruz JA, Osler M, van Staveren WA; SENECA Investigators. Dietary quality and lifestyle factors in relation to 10-year mortality in older Europeans: the SENECA study. *Am J Epidemiol*. 2002;156(10):962-968.</jrn>
33. Hodge AM, English DR, Itsiopoulos C, O'Dea K, Giles GG. Does a Mediterranean diet reduce the mortality risk associated with diabetes: evidence from the Melbourne collaborative cohort study. *Nutr Metab Cardiovasc Dis*. 2011;21(9):733-739.
34. Hodge AM, Bassett JK, Dugué PA, et al. Dietary inflammatory index or Mediterranean diet score as risk factors for total and cardiovascular mortality. *Nutr Metab Cardiovasc Dis*. 2018;28(5):461-469.
35. Hu EA, Steffen LM, Coresh J, Appel LJ, Rebholz CM. Adherence to the Healthy Eating Index-2015 and other dietary patterns may reduce risk of cardiovascular disease, cardiovascular mortality, and all-cause mortality. *J Nutr*. 2020;150(2):312-321.
36. Hulsegge G, Looman M, Smit HA, Daviglus ML, van der Schouw YT, Verschuren WM. Lifestyle changes in young adulthood and middle age and risk of cardiovascular disease and all-cause mortality: the Doetinchem cohort study. *J Am Heart Assoc*. 2016;5(1):e002432.
37. Kaluza J, Håkansson N, Brzozowska A, Wolk A. Diet quality and mortality: a population-based prospective study of men. *Eur J Clin Nutr*. 2009;63(4):451-457.
38. Kaluza J, Håkansson N, Harris HR, Orsini N, Michaëlsson K, Wolk A. Influence of anti-inflammatory diet and smoking on mortality and survival in men and women: two prospective cohort studies. *J Intern Med*. 2019;285(1):75-91.
39. Kant AK, Schatzkin A, Graubard BI, Schairer C. A prospective study of diet quality and mortality in women. *JAMA*. 2000;283(16):2109-2115.

© 2021 English LK et al. *JAMA Network Open.*

40. Kant AK, Graubard BI, Schatzkin A. Dietary patterns predict mortality in a national cohort: the National Health Interview Surveys, 1987 and 1992. *J Nutr*. 2004;134(7):1793-1799.
41. Kant AK, Leitzmann MF, Park Y, Hollenbeck A, Schatzkin A. Patterns of recommended dietary behaviors predict subsequent risk of mortality in a large cohort of men and women in the United States. *J Nutr*. 2009;139(7):1374-1380.
42. Kappeler R, Eichholzer M, Rohrmann S. Meat consumption and diet quality and mortality in NHANES III. *Eur J Clin Nutr*. 2013;67(6):598-606.
43. Kim JY, Ko YJ, Rhee CW, et al. Cardiovascular health metrics and all-cause and cardiovascular disease mortality among middle-aged men in Korea: the Seoul male cohort study. *J Prev Med Public Health*. 2013;46(6):319-328.
44. Kim H, Caulfield LE, Rebholz CM. Healthy plant-based diets are associated with lower risk of all-cause mortality in US adults. *J Nutr*. 2018;148(4):624-631.
45. Kim H, Caulfield LE, Garcia-Larsen V, Steffen LM, Coresh J, Rebholz CM. Plant-based diets are associated with a lower risk of incident cardiovascular disease, cardiovascular disease mortality, and all-cause mortality in a general population of middle-aged adults. *J Am Heart Assoc*. 2019;8(16):e012865.
46. Knoops KT, de Groot LC, Kromhout D, et al. Mediterranean diet, lifestyle factors, and 10-year mortality in elderly European men and women: the HALE project. *JAMA*. 2004;292(12):1433-1439.
47. Knoops KT, Groot de LC, Fidanza F, Alberti-Fidanza A, Kromhout D, van Staveren WA. Comparison of three different dietary scores in relation to 10-year mortality in elderly European subjects: the HALE project. *Eur J Clin Nutr*. 2006;60(6):746-755.
48. Kurotani K, Akter S, Kashino I, et al; Japan Public Health Center based Prospective Study Group. Quality of diet and mortality among Japanese men and women: Japan Public Health Center based prospective study. *BMJ*. 2016;352:i1209.
49. Kurotani K, Honjo K, Nakaya T, et al; Japan Public Health Center-based Prospective Study Group. Diet quality affects the association between census-based neighborhood

© 2021 English LK et al. *JAMA Network Open.*

deprivation and all-cause mortality in Japanese men and women: the Japan Public Health Center-Based Prospective Study. *Nutrients*. 2019;11(9):E2194.
50. Lagiou P, Trichopoulos D, Sandin S, et al. Mediterranean dietary pattern and mortality among young women: a cohort study in Sweden. *Br J Nutr*. 2006;96(2):384-392.
51. Lasheras C, Fernandez S, Patterson AM. Mediterranean diet and age with respect to overall survival in institutionalized, nonsmoking elderly people. *Am J Clin Nutr*. 2000;71(4):987-992.
52. Lassale C, Gunter MJ, Romaguera D, et al. Diet quality scores and prediction of all-cause, cardiovascular and cancer mortality in a Pan-European cohort study. *PLoS One*. 2016;11(7):e0159025.
53. Lim J, Lee Y, Shin S, et al. An association between Diet Quality Index for Koreans (DQI-K) and total mortality in Health Examinees Gem (HEXA-G) study. *Nutr Res Pract*. 2018;12(3):258-264.
54. Limongi F, Noale M, Gesmundo A, Crepaldi G, Maggi S. Adherence to the Mediterranean diet and all-cause mortality risk in an elderly Italian population: data from the ILSA Study. *J Nutr Health Aging*. 2017;21(5):505-513.
55. Liu YH, Gao X, Mitchell DC, Wood GC, Still CD, Jensen GL. Diet quality is associated with mortality in adults aged 80 years and older: a prospective study. *J Am Geriatr Soc*. 2019;67(10):2180-2185.
56. Loprinzi PD, Addoh O, Mann JR. Association between dietary behavior and mortality among American adults with mobility limitations. *Disabil Health J*. 2018;11(1):126-129.
57. Mai V, Kant AK, Flood A, Lacey JV Jr, Schairer C, Schatzkin A. Diet quality and subsequent cancer incidence and mortality in a prospective cohort of women. *Int J Epidemiol*. 2005;34(1):54-60.
58. Martínez-Gómez D, Guallar-Castillón P, León-Muñoz LM, López-García E, Rodríguez-Artalejo F. Combined impact of traditional and non-traditional health behaviors on mortality: a national prospective cohort study in Spanish older adults. *BMC Med*. 2013;11:47.
59. Martínez-González MA, Guillén-Grima F, De Irala J, et al. The Mediterranean diet is associated with a reduction in

© 2021 English LK et al. *JAMA Network Open.*

premature mortality among middle-aged adults. *J Nutr*. 2012;142(9):1672-1678.
60. Martínez-González MA, Sánchez-Tainta A, Corella D, et al; PREDIMED Group. A provegetarian food pattern and reduction in total mortality in the Prevención con Dieta Mediterránea (PREDIMED) study. *Am J Clin Nutr*. 2014;100(suppl 1):320S-328S.
61. McCullough ML, Patel AV, Kushi LH, et al. Following cancer prevention guidelines reduces risk of cancer, cardiovascular disease, and all-cause mortality. *Cancer Epidemiol Biomarkers Prev*. 2011;20(6):1089-1097.
62. McNaughton SA, Bates CJ, Mishra GD. Diet quality is associated with all-cause mortality in adults aged 65 years and older. *J Nutr*. 2012;142(2):320-325.
63. Menotti A, Alberti-Fidanza A, Fidanza F, Lanti M, Fruttini D. Factor analysis in the identification of dietary patterns and their predictive role in morbid and fatal events. *Public Health Nutr*. 2012;15(7):1232-1239.
64. Menotti A, Kromhout D, Puddu PE, et al. Baseline fatty acids, food groups, a diet score and 50-year all-cause mortality rates: an ecological analysis of the Seven Countries Study. *Ann Med*. 2017;49(8):718-727.
65. Michels KB, Wolk A. A prospective study of variety of healthy foods and mortality in women. *Int J Epidemiol*. 2002;31(4):847-854.
66. Mitrou PN, Kipnis V, Thiébaut AC, et al. Mediterranean dietary pattern and prediction of all-cause mortality in a US population: results from the NIH-AARP Diet and Health Study. *Arch Intern Med*. 2007;167(22):2461-2468.
67. Mokhtari Z, Sharafkhah M, Poustchi H, et al. Adherence to the Dietary Approaches to Stop Hypertension (DASH) diet and risk of total and cause-specific mortality: results from the Golestan cohort study. *Int J Epidemiol*. 2019;48(6):1824-1838.
68. Muller DC, Murphy N, Johansson M, et al. Modifiable causes of premature death in middle-age in Western Europe: results from the EPIC cohort study. *BMC Med*. 2016;14:87.
69. Mursu J, Steffen LM, Meyer KA, Duprez D, Jacobs DR Jr. Diet quality indexes and mortality in postmenopausal women: the Iowa Women's Health Study. *Am J Clin Nutr*. 2013;98(2):444-453.

© 2021 English LK et al. *JAMA Network Open.*

70. Nakamura Y, Ueshima H, Okamura T, et al; National Integrated Project for Prospective Observation of Non-Communicable Diseases and its Trends in the Aged, 1980 Research Group. A Japanese diet and 19-year mortality: national integrated project for prospective observation of non-communicable diseases and its trends in the aged, 1980. *Br J Nutr*. 2009;101(11):1696-1705.
71. Neelakantan N, Koh WP, Yuan JM, van Dam RM. Diet-quality indexes are associated with a lower risk of cardiovascular, respiratory, and all-cause mortality among Chinese adults. *J Nutr*. 2018;148(8):1323-1332.
72. Nilsson LM, Winkvist A, Brustad M, et al. A traditional Sami diet score as a determinant of mortality in a general northern Swedish population. *Int J Circumpolar Health*. 2012;71(0):1-12.
73. Oba S, Nagata C, Nakamura K, et al. Diet based on the Japanese Food Guide Spinning Top and subsequent mortality among men and women in a general Japanese population. *J Am Diet Assoc*. 2009;109(9):1540-1547.</jrn>
74. Okada E, Nakamura K, Ukawa S, et al. The Japanese food score and risk of all-cause, CVD and cancer mortality: the Japan collaborative cohort study. *Br J Nutr*. 2018;120(4):464-471.
75. Olsen A, Egeberg R, Halkjær J, Christensen J, Overvad K, Tjønneland A. Healthy aspects of the Nordic diet are related to lower total mortality. *J Nutr*. 2011;141(4):639-644.
76. Osler M, Heitmann BL, Høidrup S, Jørgensen LM, Schroll M. Food intake patterns, self rated health and mortality in Danish men and women: a prospective observational study. *J Epidemiol Community Health*. 2001;55(6):399-403.
77. Panizza CE, Shvetsov YB, Harmon BE, et al. Testing the predictive validity of the Healthy Eating Index-2015 in the multiethnic cohort: is the score associated with a reduced risk of all-cause and cause-specific mortality? *Nutrients*. 2018;10(4):E452.
78. Park YM, Steck SE, Fung TT, et al. Mediterranean diet and mortality risk in metabolically healthy obese and metabolically unhealthy obese phenotypes. *Int J Obes (Lond)*. 2016;40(10):1541-1549.

© 2021 English LK et al. *JAMA Network Open.*

79. Park YM, Fung TT, Steck SE, et al. Diet quality and mortality risk in metabolically obese normal-weight adults. *Mayo Clin Proc*. 2016;91(10):1372-1383.
80. Prinelli F, Yannakoulia M, Anastasiou CA, et al. Mediterranean diet and other lifestyle factors in relation to 20-year all-cause mortality: a cohort study in an Italian population. *Br J Nutr*. 2015;113(6):1003-1011.
81. Reedy J, Krebs-Smith SM, Miller PE, et al. Higher diet quality is associated with decreased risk of all-cause, cardiovascular disease, and cancer mortality among older adults. *J Nutr*. 2014;144(6):881-889.
82. Roswall N, Sandin S, Löf M, et al. Adherence to the Healthy Nordic Food Index and total and cause-specific mortality among Swedish women. *Eur J Epidemiol*. 2015;30(6):509-517.
83. Seymour JD, Calle EE, Flagg EW, Coates RJ, Ford ES, Thun MJ; American Cancer Society. Diet quality index as a predictor of short-term mortality in the American Cancer Society Cancer Prevention Study II nutrition cohort. *Am J Epidemiol*. 2003;157(11):980-988.
84. Shah NS, Leonard D, Finley CE, et al. Dietary patterns and long-term survival: a retrospective study of healthy primary care patients. *Am J Med*. 2018;131(1):48-55.
85. Shahar DR, Yu B, Houston DK, et al; Health, Aging and Body Composition Study. Dietary factors in relation to daily activity energy expenditure and mortality among older adults. *J Nutr Health Aging*. 2009;13(5):414-420.
86. Shivappa N, Hebert JR, Kivimaki M, Akbaraly T. Alternative Healthy Eating Index 2010, Dietary Inflammatory Index and risk of mortality: results from the Whitehall II cohort study and meta-analysis of previous Dietary Inflammatory Index and mortality studies. *Br J Nutr*. 2017;118(3):210-221.
87. Shvetsov YB, Harmon BE, Ettienne R, et al. The influence of energy standardisation on the alternate Mediterranean diet score and its association with mortality in the multiethnic cohort. *Br J Nutr*. 2016;116(9):1592-1601.
88. Sijtsma FP, Soedamah-Muthu SS, de Hoon SE, Jacobs DR Jr, Kromhout D. Healthy eating and survival among elderly men with and without cardiovascular-metabolic diseases. *Nutr Metab Cardiovasc Dis*. 2015;25(12):1117-1124.

© 2021 English LK et al. *JAMA Network Open.*

89. Sjögren P, Becker W, Warensjö E, et al. Mediterranean and carbohydrate-restricted diets and mortality among elderly men: a cohort study in Sweden. *Am J Clin Nutr*. 2010;92(4):967-974.
90. Sotos-Prieto M, Bhupathiraju SN, Mattei J, et al. Association of changes in diet quality with total and cause-specific mortality. *N Engl J Med*. 2017;377(2):143-153.
91. Stefler D, Malyutina S, Kubinova R, et al. Mediterranean diet score and total and cardiovascular mortality in Eastern Europe: the HAPIEE Study. *Eur J Nutr*. 2017;56(1):421-429.
92. Struijk EA, Beulens JW, May AM, et al. Dietary patterns in relation to disease burden expressed in disability-adjusted life years. *Am J Clin Nutr*. 2014;100(4):1158-1165.
93. Thorpe RJ Jr, Wilson-Frederick SM, Bowie JV, et al. Health behaviors and all-cause mortality in African American men. *Am J Mens Health*. 2013;7(4 suppl):8S-18S.
94. Tognon G, Rothenberg E, Eiben G, Sundh V, Winkvist A, Lissner L. Does the Mediterranean diet predict longevity in the elderly? A Swedish perspective. *Age (Dordr)*. 2011;33(3):439-450.
95. Tognon G, Nilsson LM, Lissner L, et al. The Mediterranean diet score and mortality are inversely associated in adults living in the subarctic region. *J Nutr*. 2012;142(8):1547-1553.
96. Tognon G, Lissner L, Sæbye D, Walker KZ, Heitmann BL. The Mediterranean diet in relation to mortality and CVD: a Danish cohort study. *Br J Nutr*. 2014;111(1):151-159.
97. Tong TY, Wareham NJ, Khaw KT, Imamura F, Forouhi NG. Prospective association of the Mediterranean diet with cardiovascular disease incidence and mortality and its population impact in a non-Mediterranean population: the EPIC-Norfolk Study. *BMC Med*. 2016;14(1):135.
98. Trichopoulou A, Costacou T, Bamia C, Trichopoulos D. Adherence to a Mediterranean diet and survival in a Greek population. *N Engl J Med*. 2003;348(26):2599-2608.
99. Trichopoulou A, Orfanos P, Norat T, et al. Modified Mediterranean diet and survival: EPIC-elderly prospective cohort study. *BMJ*. 2005;330(7498):991.

© 2021 English LK et al. *JAMA Network Open.*

100. Trichopoulou A, Bamia C, Trichopoulos D. Anatomy of health effects of Mediterranean diet: Greek EPIC prospective cohort study. *BMJ*. 2009;338:b2337.
101. van Dam RM, Li T, Spiegelman D, Franco OH, Hu FB. Combined impact of lifestyle factors on mortality: prospective cohort study in US women. *BMJ*. 2008;337:a1440.
102. van den Brandt PA. The impact of a Mediterranean diet and healthy lifestyle on premature mortality in men and women. *Am J Clin Nutr*. 2011;94(3):913-920.
103. van Lee L, Geelen A, Kiefte-de Jong JC, et al. Adherence to the Dutch dietary guidelines is inversely associated with 20-year mortality in a large prospective cohort study. *Eur J Clin Nutr*. 2016;70(2):262-268.
104. Voortman T, Kiefte-de Jong JC, Ikram MA, et al. Adherence to the 2015 Dutch dietary guidelines and risk of non-communicable diseases and mortality in the Rotterdam study. *Eur J Epidemiol*. 2017;32(11):993-1005.
105. Vormund K, Braun J, Rohrmann S, Bopp M, Ballmer P, Faeh D. Mediterranean diet and mortality in Switzerland: an alpine paradox? *Eur J Nutr*. 2015;54(1):139-148.
106. Wahlqvist ML, Darmadi-Blackberry I, Kouris-Blazos A, et al. Does diet matter for survival in long-lived cultures? Asia Pac J Clin Nutr. 2005;14(1):2-6.
107. Warensjö Lemming E, Byberg L, Wolk A, Michaëlsson K. A comparison between two healthy diet scores, the modified Mediterranean diet score and the Healthy Nordic Food Index, in relation to all-cause and cause-specific mortality. *Br J Nutr*. 2018;119(7):836-846.
108. Whalen KA, Judd S, McCullough ML, Flanders WD, Hartman TJ, Bostick RM. Paleolithic and Mediterranean diet pattern scores are inversely associated with all-cause and cause-specific mortality in adults. *J Nutr*. 2017;147(4):612-620.
109. Yu D, Sonderman J, Buchowski MS, et al. Healthy eating and risks of total and cause-specific death among low-income populations of African-Americans and other adults in the southeastern United States: a prospective cohort study. *PLoS Med*. 2015;12(5):e1001830.
110. Zaslavsky O, Zelber-Sagi S, Hebert JR, et al. Biomarker-calibrated nutrient intake and healthy diet index associations with mortality risks among older and frail

© 2021 English LK et al. *JAMA Network Open.*

women from the Women's Health Initiative. *Am J Clin Nutr*. 2017;105(6):1399-1407.
111. Zaslavsky O, Zelber-Sagi S, Shikany JM, et al. Anatomy of the Mediterranean diet and mortality among older women with frailty. *J Nutr Gerontol Geriatr*. 2018;37(3-4):269-281.
112. Anderson AL, Harris TB, Tylavsky FA, et al; Health ABC Study. Dietary patterns and survival of older adults. *J Am Diet Assoc*. 2011;111(1):84-91.
113. Atkins JL, Whincup PH, Morris RW, Lennon LT, Papacosta O, Wannamethee SG. Dietary patterns and the risk of CVD and all-cause mortality in older British men. *Br J Nutr*. 2016;116(7):1246-1255.
114. Bamia C, Trichopoulos D, Ferrari P, et al. Dietary patterns and survival of older Europeans: the EPIC-Elderly Study (European Prospective Investigation into Cancer and Nutrition). *Public Health Nutr*. 2007;10(6):590-598.
115. Brunner EJ, Mosdøl A, Witte DR, et al. Dietary patterns and 15-y risks of major coronary events, diabetes, and mortality. *Am J Clin Nutr*. 2008;87(5):1414-1421.
116. Granic A, Andel R, Dahl AK, Gatz M, Pedersen NL. Midlife dietary patterns and mortality in the population-based study of Swedish twins. *J Epidemiol Community Health*. 2013;67(7):578-586.
117. Hamer M, McNaughton SA, Bates CJ, Mishra GD. Dietary patterns, assessed from a weighed food record, and survival among elderly participants from the United Kingdom. *Eur J Clin Nutr*. 2010;64(8):853-861.
118. Heidemann C, Schulze MB, Franco OH, van Dam RM, Mantzoros CS, Hu FB. Dietary patterns and risk of mortality from cardiovascular disease, cancer, and all causes in a prospective cohort of women. *Circulation*. 2008;118(3):230-237.
119. Hoffmann K, Boeing H, Boffetta P, et al. Comparison of two statistical approaches to predict all-cause mortality by dietary patterns in German elderly subjects. *Br J Nutr*. 2005;93(5):709-716.
120. Hsiao PY, Mitchell DC, Coffman DL, et al. Dietary patterns and relationship to obesity-related health outcomes and mortality in adults 75 years of age or greater. *J Nutr Health Aging*. 2013;17(6):566-572.

© 2021 English LK et al. *JAMA Network Open.*

121. Krieger JP, Cabaset S, Pestoni G, Rohrmann S, Faeh D; Swiss National Cohort Study Group. Dietary patterns are associated with cardiovascular and cancer mortality among Swiss adults in a census-linked cohort. *Nutrients*. 2018;10(3):E313.
122. Martínez-González MA, Zazpe I, Razquin C, et al; PREDIMED GROUP. Empirically-derived food patterns and the risk of total mortality and cardiovascular events in the PREDIMED study. *Clin Nutr*. 2015;34(5):859-867.
123. Masala G, Ceroti M, Pala V, et al. A dietary pattern rich in olive oil and raw vegetables is associated with lower mortality in Italian elderly subjects. *Br J Nutr*. 2007;98(2):406-415.
124. Menotti A, Alberti-Fidanza A, Fidanza F. The association of the Mediterranean Adequacy Index with fatal coronary events in an Italian middle-aged male population followed for 40 years. *Nutr Metab Cardiovasc Dis*. 2012;22(4):369-375.
125. Menotti A, Puddu PE, Lanti M, Maiani G, Catasta G, Fidanza AA. Lifestyle habits and mortality from all and specific causes of death: 40-year follow-up in the Italian Rural Areas of the Seven Countries Study. *J Nutr Health Aging*. 2014;18(3):314-321.
126. Menotti A, Puddu PE, Maiani G, Catasta G. Cardiovascular and other causes of death as a function of lifestyle habits in a quasi extinct middle-aged male population. A 50-year follow-up study. *Int J Cardiol*. 2016;210:173-178.
127. Nanri A, Mizoue T, Shimazu T, et al; Japan Public Health Center-Based Prospective Study Group. Dietary patterns and all-cause, cancer, and cardiovascular disease mortality in Japanese men and women: the Japan public health center-based prospective study. *PLoS One*. 2017;12(4):e0174848.
128. Odegaard AO, Koh WP, Yuan JM, Gross MD, Pereira MA. Dietary patterns and mortality in a Chinese population. *Am J Clin Nutr*. 2014;100(3):877-883.
129. Waijers PM, Ocké MC, van Rossum CT, et al. Dietary patterns and survival in older Dutch women. *Am J Clin Nutr*. 2006;83(5):1170-1176.
130. Zazpe I, Sánchez-Tainta A, Toledo E, Sánchez-Villegas A, Martínez-González MA. Dietary patterns and total mortality

© 2021 English LK et al. *JAMA Network Open.*

in a Mediterranean cohort: the SUN project. *J Acad Nutr Diet*. 2014;114(1):37-47.
131. Zhao W, Ukawa S, Okada E, et al. The associations of dietary patterns with all-cause mortality and other lifestyle factors in the elderly: an age-specific prospective cohort study. *Clin Nutr*. 2019;38(1):288-296.
132. Mihrshahi S, Ding D, Gale J, Allman-Farinelli M, Banks E, Bauman AE. Vegetarian diet and all-cause mortality: evidence from a large population-based Australian cohort - the 45 and Up Study. *Prev Med*. 2017;97:1-7.
133. Song M, Fung TT, Hu FB, et al. Association of animal and plant protein intake with all-cause and cause-specific mortality. *JAMA Intern Med*. 2016;176(10):1453-1463.
134. Key TJ, Appleby PN, Spencer EA, Travis RC, Roddam AW, Allen NE. Mortality in British vegetarians: results from the European Prospective Investigation into Cancer and Nutrition (EPIC-Oxford). *Am J Clin Nutr*. 2009;89(5):1613S-1619S.
135. Orlich MJ, Singh PN, Sabaté J, et al. Vegetarian dietary patterns and mortality in Adventist Health Study 2. *JAMA Intern Med*. 2013;173(13):1230-1238.
136. Chang-Claude J, Hermann S, Eilber U, Steindorf K. Lifestyle determinants and mortality in German vegetarians and health-conscious persons: results of a 21-year follow-up. *Cancer Epidemiol Biomarkers Prev*. 2005;14(4):963-968.
137. Héroux M, Janssen I, Lam M, et al. Dietary patterns and the risk of mortality: impact of cardiorespiratory fitness. *Int J Epidemiol*. 2010;39(1):197-209.
138. Meyer J, Döring A, Herder C, Roden M, Koenig W, Thorand B. Dietary patterns, subclinical inflammation, incident coronary heart disease and mortality in middle-aged men from the MONICA/KORA Augsburg cohort study. *Eur J Clin Nutr*. 2011;65(7):800-807.
139. Schnabel L, Kesse-Guyot E, Allès B, et al. Association between ultraprocessed food consumption and risk of mortality among middle-aged adults in France. *JAMA Intern Med*. 2019;179(4):490-498.
140. Kim H, Hu EA, Rebholz CM. Ultra-processed food intake and mortality in the USA: results from the Third National Health and Nutrition Examination Survey (NHANES III, 1988-1994). *Public Health Nutr*. 2019;22(10):1777-1785.

© 2021 English LK et al. *JAMA Network Open.*

141. Rico-Campà A, Martínez-González MA, Alvarez-Alvarez I, et al. Association between consumption of ultra-processed foods and all cause mortality: SUN prospective cohort study. *BMJ*. 2019;365:1949.
142. US Department of Agriculture. Nutrition Evidence Systematic Review. Systematic review question: what is the relationship between dietary patterns consumed and all-cause mortality? Conclusion statement. Accessed July 27, 2021. https://nesr.usda.gov/2020-dietary-guidelines-advisory-committee-systematic-reviews

© 2021 English LK et al. *JAMA Network Open.*
